# Age-Dependent Changes of Thinking about Verbs

**DOI:** 10.3389/fnbeh.2017.00040

**Published:** 2017-03-14

**Authors:** Carolina Bonivento, Barbara Tomasino, Marco Garzitto, Sara Piccin, Franco Fabbro, Paolo Brambilla

**Affiliations:** ^1^Department of Experimental Clinical Medicine, University of UdineUdine, Italy; ^2^IRCCS “E. Medea” Scientific Institute, Polo del Friuli Venezia GiuliaUdine, Italy; ^3^Department of Human Sciences, University of UdineUdine, Italy; ^4^Department of Neurosciences and Mental Health, Fondazione IRCCS Ca' Granda Ospedale Maggiore Policlinico, University of MilanMilan, Italy; ^5^Department of Psychiatry and Behavioral Sciences, University of Texas Health Science Center at HoustonHouston, TX, USA

**Keywords:** emotional valence, arousal, imageability, age of acquisition, action concepts, emotion concepts, verbs

## Abstract

We investigated the knowledge of emotional and motor verbs in children and adolescents from three age ranges (8–11, 12–15, 16–19 years). Participants estimated the verbs familiarity, age of acquisition, valence, arousal, imageability, and motor- and emotion-relatedness. Participants were familiar with the verbs in our dataset. The younger (8–11) attributed an emotional character to the verbs less frequently than the middle (12–15) and the older (16–19) groups. In the 8–11 group males rated the verbs as emotion-related less frequently than females. Results indicate that processing verbal concepts as emotion-related develops gradually, and after 12–15 is rather stable. The age of acquisition (AoA) develops late: the older (16–19) had a higher awareness in reporting that they learnt the verbs earlier as compared to the estimations made by the younger (8–11 and 12–15). AoA positively correlated with attribution of emotion relatedness meaning that emotion-related verbs were learned later. Arousal was comparable across ages. Also it increased when attributing motor relatedness to verbs and decreased when attributing emotion relatedness. Reporting the verbs' affective valence (happy vs. unhappy) changes with age: younger (8–11) judged the verbs generally more “happy” than both the older groups. Instead the middle and the older group did not show differences. Happiness increased when processing the verbs as motor related and decreased when processing the verbs as emotion related. Age affected imageability: the younger (8–11) considered the verbs easier to be imagined than the two older groups, suggesting that at this age vividness estimation is still rough, while after 12–15 is stable as the 12–15 and 15–19 group did not differ. Imageability predicted arousal, AoA, emotion- and motor-relatedness indicating that this index influences the way verbs are processed. Imageability was positively correlated to emotion relatedness, indicating that such verbs were harder to be imagined, and negatively to motor relatedness. Imageablity positively correlated with valence meaning that verbs receiving positive valence were also those that were hard to be imagined, and negatively correlated with arousal, meaning that verbs that were harder to be imagined elicited low physiological activation. Our results give an insight in the development of emotional and motor-related verbs representations.

## Introduction

Understanding emotions and knowing the right words to label emotional states are crucial factors in in humans' social interaction. An important aspect of human interactions is to adequately express emotions. Besides behaviors and facial expressions, a capacity that is unique to humans is to use verbal language for externalizing affective states. The knowledge of verbal labels for emotions is reported to appear early during language acquisition, with children using affective words within the second year of age (Bretherton and Beeghly, 1982; Izard and Harris, 1995; Denham, 1998; see also Baron-Cohen et al., 2010). At first, children use emotional terms for describing their own internal states (20–14 moths) and later with reference to others feelings (3–3.5 years of age; Reilly et al., 1990). Longitudinal studies collected maternal reports on the use of emotional vocabulary in children in their second and third years of age revealing that the use of affective adjectives emerges as early as 20 months of age and rapidly increases through the third year (Bretherton et al., 1981; Bretherton and Beeghly, 1982). Ridgeway et al. (1985) extended the investigation to older children assessing the ability to understand and use 125 emotional terms of a sample of 270 toddlers and preschoolers children (from 18 to 71 months of age). The authors used the data collected to compile a catalog with norms for each age range to be used in studies on emotional development, although their study left out children at school age. A study on the emotional words comprehension in school aged children and adolescents (range 4–16 years) was carried out more recently by Baron-Cohen et al. (2010). This last study found that the emotional lexicon almost doubles every 2 years between 4 and 11 year while it does not increase significantly from 12 to 16.

The majority of the studies collected empirical evidences on emotional language development but did not propose theories on how it is acquired with respect to non-emotional terms. A proposal about emotional words' learning mechanism come from Moseley et al. (2012) who linked emotional words learning to bodily actions (see also Kiefer and Pulvermüller, 2012). However, these authors only advanced a hypothesis indirectly drawn from their fMRI experiments on adults where they found activation in the sensorimotor cortex during a task involving emotional words (Moseley et al., 2012). Moseley et al. (2012) interpreted the results in terms of emotional words, that convey the knowledge of abstract concepts, having a sensorimotor representation and argued that, being represented in such a format they have an embodied representation. Other studies do not found sensorimotor activation for emotional words and suggest that the context in which verbs are processed influence the activation pattern, see for example our previous work on adults participants (e.g., Papeo et al., 2012; Tomasino et al., 2013, 2014), in patients studies (e.g., **?**) or from a theoretical point of view (e.g., Tomasino and Rumiati, 2013). For instance, Papeo et al. (2012) found that motor areas were recruited during silent reading of abstract verbs. However, this recruitment occurred only when the task was preceded by a motor-imagery but not by a visual-imagery task (Papeo et al., 2012), thus suggesting that it depended on the context (i.e., the environment with the contingent stimuli it contains).

Similarly, in developmental studies some authors argue that it is the context that influences the way the linguistic semantic contents are acquired (Barak et al., 2012). From empirical observation, the verbs describing emotional internal states, seem to show a developmental delay with respect to other categories of verbs, but also with respect to affective words (non-verbs; see Barak et al., 2012). They proposed that children must first master the syntactic structures implied in sentences in which the mental state verbs typically occur. Only after this is achieved, the presence of those semantic structures may act as a cue directing the attention to the internal state content, triggering the proper processing and, eventually, the understanding of mental state verbs (see Barak et al., 2012). It takes time for this ability to appear through the exposure and learning (i.e., a semantic representation of internal states must be built and established in semantic long-term memory before it can intervene to cue toward mental state contents in a dynamic scene).

Other studies however showed that very young children (28 months) were able to label their own emotions. Moreover, children of just 3–3.5 years of age could label emotions of characters in stories (Reilly et al., 1990). These children used words (i.e., static labels) to denote very basic emotions and in contexts where there were not overt dynamic actions capturing their attention. Also, labeling simple emotions of fictional characters in a story may be a much easier than produce sentences referring to actual emotions (using mental state verbs) in everyday life and real context.

At the best of our knowledge there are no studies that established a timeline of mental state verbs acquisition through childhood and adolescence. Some works focused on development of emotional words (e.g., Baron-Cohen et al., 2010). Words referring to feelings and affective states were widely used in studies investigating the modulation of emotions on cognitive processes, mainly focusing on attention and memory processes or executive functions, on healthy individuals and in mental illness, in populations of both children and adults (see Bellani et al., 2012 for a review). For instance emotional norms for more than 600 English words had been developed by Bradley and Lang (1999) and provided a set of verbal material to be used in studies on emotions on adults populations. Such emotional verbal stimuli had been also used in studies on the emotional bias on cognition in psychiatric patients, such as anxious, depressed, or bipolar patients, as well as in healthy volunteers, adults and children/adolescents (see Bellani et al., 2012 for a review).

However, in such studies verbs instead were left behind, although they appear to follow an independent (at least partially) developmental timeline (see Barak et al., 2012). In addition, verbs are more suitable to trigger emotional imagery that requires imagining feeling an emotion (such as to love or to suffer) than emotional words (i.e., static labels). Emotion verbs can be powerful tools for investigating the processes of emotional development, or deviance in psychopathology. Thus, it is worth to deepen our knowledge of verbs conveying inner states, understand their developmental trajectory, possibly extending the investigation to adults and elder samples of people.

The aim of the present work is to attempt to figure out the way of processing an exhaustive set of mental state verbs, as compared to non-emotional action verbs. We expected that since mental state verbs are complex concepts, different age class will change the way these concepts are processed. In addition we were interested in measuring the additional effects played by imageability, arousal, and valence on the way children and adolescents mentally represent mental state verbs.

## Methods

### Participants

Five hundred eighty-eight children and adolescents were recruited in primary, secondary and high schools from two regions in the northeast and west-central Italy. The participants were divided into three groups by age range i.e., 8–11 (*n* = 284; *n* = 140 female); 12–15 (*n* = 162 *n* = 82 female); 16–19 (*n* = 142 *n* = 72 female) and were asked to answer to an anonymous survey^1^.

The participants were recruited in state schools that in Italy gather students from almost all social backgrounds and so are supposed to be representative of the general population of Italian children and adolescents. Students having learning or language deficits, mental retardation, physical disability such as blindness or deafness or other neurological and psychiatric conditions were excluded from the sample. The presence of one or more exclusion criteria was established on the basis of reports from the teachers and the school staff.

The study was approved by the Ethical Committee of the IRCCS “E. Medea.” Bosisio Parini (Lc).

The Principals of the schools involved, together with the Teachers and Parents' Committees, gave the consent to conduct the study, in compliance with the laws regulating the procedures for caring out anonymous surveys.

### Stimuli

Verbs describing inner affective states were uses as stimuli. As many emotions may also require the involvement of the whole body, or even an actual act (e.g., to suicide), we decided to include in the databases verbs being emotional or motor to different degrees in order to have materials suitable to directly compare emotional and motor imagery.

An initial list of *N* = *140* Italian verbs were selected by a group composed by a linguist, a clinical psychologist, and a psychiatrist. Only those items on which a unanimous agreement was achieved by all the three members of the group as to their relation to emotional states (*N* = 75) or to bodily actions (*N* = 65) were selected^2^. To reduce variability, and following to previous studies (e.g., see Tomasino et al., 2007 for details) the action-related verb list included verbs describing actions involving hand movements only. In addition, the motor-and the emotion-related verbs lists were matched for including 20 and 28 reflexive verbs respectively, since some emotional verbs list existed only in their reflexive form.

Motor- and emotional-relatedness of the verbs was additionally judged by seven independent judges who were blind to the aim of the study and who ignored the a-priori classification of the verbs, who indicated for each verb: (i) whether it was related to an emotion or feeling (Yes or No); and (ii) whether it involved a movement of the body (Yes or No). The seven judges were aware that for each verb they could say “Yes” to both questions. Fleiss' kappa indicating the degree of agreement between the judges in their ratings was performed. The Fleiss' kappa for each verb and the mean values of familiarity, emotion relatedness, motor relatedness, valence, arousal, imageability, and AOA are reported in Supplementary Tables 1–7 in Supplementary Materials.

### Task

Due to obvious time constraints while testing children, it was not possible to administer all the 140 verbs to each participant. For this reason, the 140 verbs were divided into 4 lists of 23 verbs and 2 lists of 24 verbs and used as the items of six different questionnaires (one questionnaire for each verbs list). Each participant answered to only one of the six questionnaires. Participants were asked to judge each verb and the ratings were than used to calculate indexes concerning the following dimensions:

- Familiarity—i.e., participants had to state whether they know the verb [Yes (1) or Not (0)] forming the index “percentage of know verbs.”
- Motor-relatedness—i.e., participants judged whether the verb involved the movement of a body part [Yes (1) or Not (0)] forming the index “percentage of verbs judged as involving an action or bodily movement.”
- Emotion-relatedness—i.e., participants judged whether the verb involved a feeling or an emotional state [Yes (1) or Not (0)], forming the index “percentage of verbs judged as involving an emotion or feeling.”
- Age of acquisition (AOA)—i.e., participants had to indicate at what age they reckon they learned the meaning of the verb (years; e.g., 3 years and ½).
- Arousal—i.e., participants indicated on a scale from 1 to 9 on the Self Assessment Manikin (SAM; Lang, 1980) how much arousal was triggered by the action or feeling or state described by the verb (score 1–9, 1 = very calm; 9 = very aroused).
- Emotional valence—participants had to indicate on a scale from 1 to 9 on the SAM how much happy make them feel the action or feeling or state described by the verb (score 1–9, 1 = very happy; 9 = very unhappy).
- Imageability—participants had to indicate on a scale from 1 to 11, presented in a form of a thermometer, how easy was to imagine the action or feeling or state described by the verb (score 1–11, 1 = very easy; 11 = very difficult).

The ratings given by the participants were used to calculate the indexes associated to each verb as well as to run the preliminary comparisons to test for differences due to age and/or gender.

### Statistical analysis

Due to practical contingencies that could not be overcome, different participants answered to different questionnaires containing a different sub-group of verbs. For this reasons the verbs were entered as the subject of the analyses. However, as the emotion and motor relatedness judgments were measured on a nominal scale (“YES”–“NO”) the more proper way to treat the scores was entering them in a contingency table, with fields defined by age-range and gender, and analyze the judgment frequency through a Chi2. For instance, within the age range 8–11 the verb “to love” was known by 37 children (i.e., 37 measurements) while the verb “to lie” that was in another list was answered by 40 children (i.e., 40 measurements). In all we obtained the measurements for 12,197 verbs across the 3 age ranges (5,738 in the age range 8–11; 3,400 in the age range 12–15; 3,059 in the age range 16–19). A part for the index “Familiarity” corresponding to the proportion of participants knowing a given verb on the total number of subjects, the frequencies or the means of other indexes (i.e., familiarity, emotion relatedness, motor relatedness, age of acquisition, arousal, emotional valence, and imageability) were calculated on the total number of participants answering to and knowing that verb. As an example in the 8–11 group the verb “to love” was both known and judged by 37 children out of which 5 rated this verb as “Motor.” Thus, for the verb “to love” the index “Motor” (% of “Yes” answers) was 5/37 = 0.14 or 14%. For the same verb 36/37 children judged the verb as involving an emotion or feeling, thus the index “Emotion” was 97%. Also those indexes expressed in the form of means and *SD*s (i.e., AOA, Arousal, Emotion valence, imageability) were calculated on the number of respondents for a given verb (e.g., 37 in the case of the verb “to love” in the age range 8–11). In total, across the 3 age ranges, the subjects stated to know and correctly filled the entire questionnaire for 11,749 verbs (5,494 in the age range 8–11; 3,342 in the age range 12–15; 2,913 in the age range 16–19).

#### Analysis of familiarity, emotion relatedness, and motor relatedness ratings

The indexes having “yes = 1” or “no = 0” answers (i.e., Familiarity, Emotion relatedness, and Motor relatedness) were analyzed through the Chi2 test comparing pairs of groups of subjects clustered together according to age range, gender or both age and gender (Table 1). The significance level was corrected for multiple comparisons and was set at α = 0.004^3^.

**Table 1:** **Statistical values obtained from the Chi2 tests that compared the frequencies of participants “emotional or motor judgments”**.

	**Contrasts**	***Emotional relatedness***	***Motor relatedness***
AGE	8–11 vs. 12–15 vs. 16–19	**χ_(1)_ = 86.04, *p* < 0.001**	**χ_(1)_ = 14.07, *p* < 0.001**
GENDER	FEMALES vs. MALE	**χ_(1)_ = 10.8, *p* < 0.001**	χ_(1)_ = 1.34, *p* > 0.1
AGE	8–11 vs. 12–15	**χ_(1)_ = 41.41, *p* < 0.001**	**χ_(1)_ = 10.56, *p* < 0.001**
	8–11 vs. 16–19	**χ_(1)_ = 73.81, *p* < 0.001**	**χ_(1)_ = 8.69, *p* < 0.005**
	12–15 vs. 16–19	*χ_(1)_ = 4.81, p < 0.05*	χ_(1)_ = 0.02, *p* > 0.1
AGE by GENDER (within age ranges)	f 8–11 vs. m 8–11	**χ_(1)_ = 17.49, *p* < 0.001**	χ_(1)_ = 1.98, *p* > 0.1
	f 12–15 vs. m 12–15	χ_(1)_ = 0.29, *p* > 0.1	χ_(1)_ = 0.11, *p* > 0.1
	f 16–19 vs. m 16–19	χ_(1)_ = 1.09, *p* > 0.1	χ_(1)_ = 0.00, *p* > 0.1

*The analyses investigated the effect of age (AGE—ALL 8–11 vs. ALL 12–15 vs. ALL 16–19) and gender (GENDER—FEMALES vs. MALE). Then, we looked at differences between pairs of age ranges (AGE—8–11 vs. 12–15; 8–11 vs. 16–19; 12–15 vs. 16–19) and within individuals of different gender within each age range (AGE by GENDER (within age ranges)—f 8–11 vs. m 8–11; f 12–15 vs. m 12–15, f 16–19 vs. m 16–19). Bold numbers indicate significant results. Italic numbers indicate results that reached the uncorrected significance level (α = 0.05)*.

#### Analysis of AOA, arousal, emotion valence, imageability ratings

The indexes having values' range (AOA, Arousal, Emotion valence, Imageability) were analyzed through a univariate ANOVA with age (8–11, 12–15, and 16–19) and gender (females, males) as between subject factors and the emotional and motor ratings as covariates (**Table 3**). The significance level was corrected for multiple comparisons and was set at α = 0.004^2^.

#### Regression and correlation analyses

As the final step of our study we explored how the emotional and motor judgment could predict AOA, emotional valence, arousal, and imageability. Also, as previous studies pointed out a correlation between some indexes, such as AOA and imageability (Bird et al., 2001), we also analyzed the correlations between all the measures collected.

Emotion or motor relatedness judgments were entered into regression models predicting the dependent variables: (i) arousal; (ii) emotional valence; (iii) imageability; and (iv) AOA. Separate models were run for each dependent variable using emotional or motor relatedness judgments as predictors.

Emotional and motor relatedness, emotional valence, arousal, imageability, and AOA were entered into a correlation matrix, together with age range and gender as covariates.

The regressions and correlations were carried out on a model including the data of the participants of all the age ranges. In all eight models were analyzed. All the eight models included also age range and gender as covariates. The corrected significance level was set at α ≤ 0.004.

## Results

### Familiarity

We first made sure that participants were familiar with the items we used. Participants' verbs' familiarity was almost at ceiling (8–11 = 96%, 12–15 = 98%; 16–19 = 95%) in all the 3 age groups and within both males and females (Females 8–11 = 96%, Males 8–11 = 96%; Females 12–15 = 99%; Males 12–15 = 98%; Females 16–19 = 96%; Males 16–19 = 95%).

### Emotion relatedness

**Figure 1:**
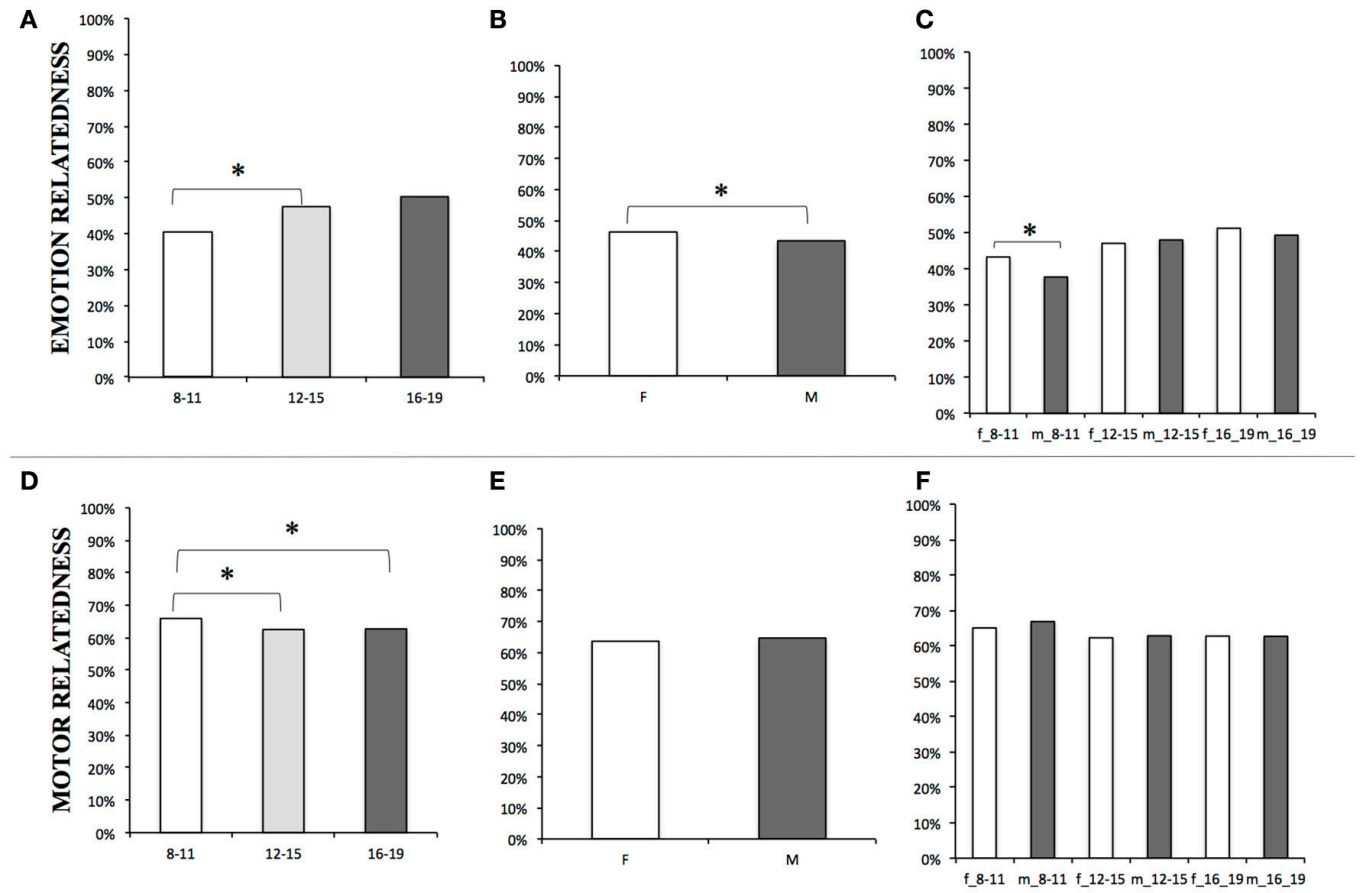
**Percentages judgments within each age range** [**(A)** emotion-related and **(D)** motor related], within females (F), and males (M) [**(B)** emotion-related and **(E)** motor related], within females **(F)**, and males (m) of each age range [**(C)** emotion-related and **(F)** motor related]. 8–11, 12–15, 16–19 = participants aged between 8 and 11, 12–15, and 16–19 years, respectively. The significant differences, surviving the Bonferroni correction, are marked (“^*^”).

Participants' judgments are shown in Figures 1A–C. Altogether, the participants judged the verbs being “Emotional” 45% of the times. The “Emotional-relatedness” rating appeared to be affected by the participants' age [χ^2^(2) = 86,04, *p* < 0.001] with the 8–11 group rating the verbs as “Emotional” less frequently (41%) than both the 12–15 (48%) [χ^2^(1) = 41.14, *p* < 0.001] and the 16–19 (50%) [χ^2^(1) = 73.81, *p* < 0.001] groups (Figure 1A).

Also, the Chi2 tests showed a main effect of gender on emotional-relatedness judgments [χ^2^(1) = 10.08, *p* < 0.001], with female rating 46% and male rating 43% of the verbs as being Emotional. However, when comparisons were run separately within each age range, the gender significantly affected the judgment only within the age range 8–11 [χ^2^(1) = 17.49, *p* < 0.001] with higher emotional ratings for female (43%) than male (38%; Figure 1C) {no significant difference for 12–15 [χ^2^(1) = 0.29, *p* > 0.1, female rating = 47%, male rating = 48%] and 16–19 [χ^2^(1) = 1.09, *p* > 0.1, female rating = 51%, male rating = 49%) groups} (see Table 1).

An estimate of the reliability of the participants' ratings was obtained, for each verb, calculating the proportion of raters' pairs that were in agreement (i.e., pairs giving the same judgment to a given verb) relatively to the number of all the possible raters' s pairs in the sample (or sub-sample) of participants. Table 2 shows that he frequencies of agreements' ranges were optimal (100 to 80%) in 52.14% of the participants, good (79 to 60%) in 29.29% of the cases and moderate/poor (<60%) in 18.57% of the subjects. The detailed reports of the judgments' agreement for each verb, within the whole sample and within age ranges are reported in Supplementary Table 1.

**Table 2:** **Frequencies of Optimal (100–80%), Good (79–60%), and Moderate/Poor (<60%) agreement ranges in the overall sample (ALL) and within each age range (8–11, 12–15, 16–19)**.

**Emotion-relatedness**	**Optimal (100–80%)**	**Good (79–60%)**	**Moderate/Poor (<60%)**	**Motor-relatedness**	**Optimal (100–80%)**	**Good (79–60%)**	**Moderate/Poor (<60%)**
ALL	52.14%	29.29%	18.57%	ALL	52.86%	22.86%	24.29%
8–11	52.86%	23.57%	23.57%	8–11	51.43%	25.00%	23.57%
12–15	52.86%	29.29%	17.86%	12–15	50.71%	27.14%	22.14%
16–19	62.14%	24.29%	13.57%	16–19	58.57%	21.43%	20.00%

### Motor relatedness

Participants' judgments are presented in Figures 1D–F and in Table 1. Verbs were rated as “Motor” 64% of the times across all the 3 age groups. Chi2 tests showed that overall “Motor-relatedness” ratings were affected by age [χ^2^(2) = 14.07, *p* < 0.001]. Children aged 8–11 rated the verbs as “Motor” more frequently (66%) than the 12–15 (63%) [χ^2^(1) = 10.56, *p* < 0.001] while the 8–11 vs. 16–19 did not survive the correction for multiple comparisons and the 12–15 vs. 16–19 did not differed between each other [16–19: χ^2^(1) = 0.02, *p* > 0.1; Figure 1).

Gender *per-se* seemed not to affect the frequencies of motor judgments. No other effects emerged. The estimate of the reliability of the participants' ratings (see Table 2) shows that he frequencies of agreements' ranges were optimal (100 to 80%) in 52.86% of the participants, good (79 to 60%) in 22.86% of the cases and moderate/poor (<60%) in 24.29% of the subjects.

### Arousal

**Figure 2:**
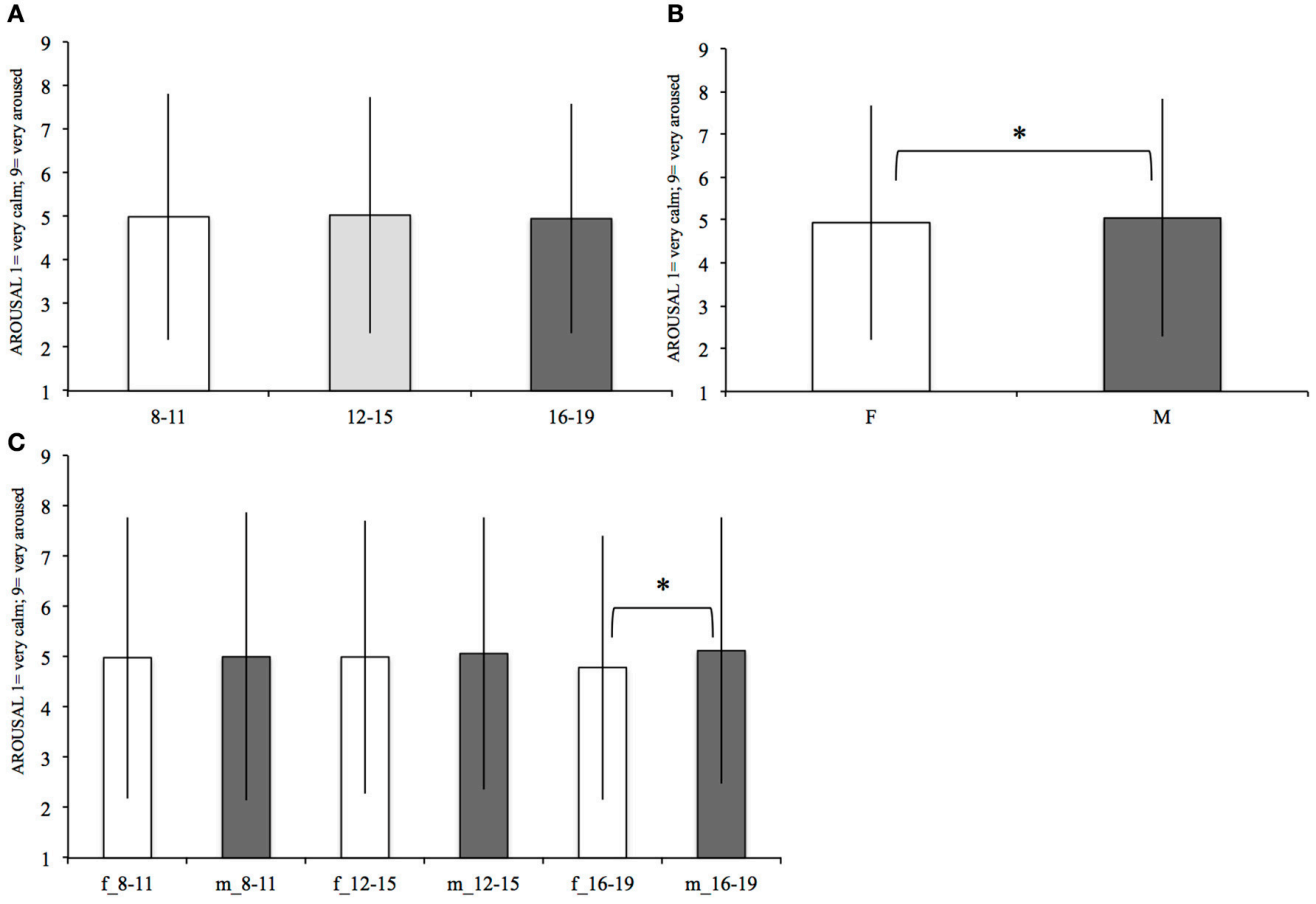
**Mean arousal within each age range (A)**. Within females (F) and males (M) **(B)**, and within females and males of each age range **(C)**. The significant differences, surviving the Bonferroni correction, are marked (“^*^”). Bars denote standard deviations.

Mean arousal values for females and males in each age range are represented in Figure 2 and in Table 3. Age had not a main effect on arousal (see Table 3): on average the 8–11 years old (mean = 4.99, *SD* = 2.83), the 12–15 (mean = 5.03, *SD* = 2.71), and the 16–19 (mean = 4.95, *SD* = 2.64) did not significantly differ between each other.

**Table 3:** **Statistical values obtained from the univariate ANOVA and ***Post-hoc*** comparisons exploring the effect of age and gender, and the interactions between these factors, on age of acquisition (AOA), arousal (1 = very calm; 9 = very aroused), valence (1 = very happy; 9 = very unhappy), and imageability (1 = very easy; 11 = very difficult)**.

		**AOA**	**Arousal**	**Valence**	**Imaginability**
Main effect	Age	***F*_(2, 11, 741)_ = 20.28**;***p* < 0.001**	*F*_(2, 11, 741)_ = 2.78; *p* = 0.06	***F*_(2, 11, 741)_ = 29.75; *p* < 0.00**	***F*_(2, 11, 741)_ = 21.38; *p* < 0.00**
*Post-hoc*	8–11 vs. 12–15	*F*_(1, 8832)_ = 0.05; *p* = 0.83	–	***F*_(1, 8832)_ = 33.83; *p* < 0.00**	***F*_(1, 8832)_ = 36.27; *p* < 0.00**
	8–11 vs. 16–19	***F*_(1, 8403)_ = 35.47; *p* < 0.001**	–	***F*_(1, 8403)_ = 46.82; *p* < 0.00**	***F*_(1, 8403)_ = 23.95; *p* < 0.00**
	12–15 vs. 16–19	***F*_(1, 6251)_ = 30.38; *p* < 0.001**	–	*F*_(1, 6251)_ = 1.18; *p* = 0.28	*F*_(1, 6251)_ = 0.84; *p* = 0.36
Main effect	Gender	*F*_(1, 11, 741)_ = 0.31; *p* = 0.5	***F*_(1, 11, 741)_ = 4.88; *p* = 0.03**	*F_(1, 11, 741)_ = 3.71; p = 0.05*	*F_(1, 11, 741)_ = 5.88; p = 0.02*
Interaction	Age by Gender	*F_(2, 11, 741)_ = 4.20; p = 0.02*	***F*_(2, 11, 741)_ = 4.52; *p* = 0.01**	*F*_(2, 11, 741)_ = 0.07; *p* = 0.93	***F*_(2, 11, 741)_ = 13.78; *p* < 0.00**
Within Age ranges	f 8–11 vs. m 8–11	–	*F_(1, 5490)_ = 0.23; p = 0.63*	–	*F_(1, 5490)_ = 6.34; p = 0.01*
	f 12–15 vs. m 12–15	–	*F_(1, 3338)_ = 0.83; p = 0.36*	–	***F*_(1, 3338)_ = 24.58; *p* < 0.00**
	f 16–19 vs. m 16–19	–	***F*_(1, 2909)_ = 11.27; *p* < 0.00**	–	*F_(1, 2909)_ = 668; p = 0.01*

*Bold numbers indicate significant results. Italic numbers indicate results below the uncorrected significance level (α = 0.05). 8–11, 12–15, 16–19 = age range from 8 to 11; 12 to 15, and 16 to 19 years respectively; f = females m = males 8*.

Gender [*F*_(1, 11, 741)_ = 4.88, *p* = 0.03; see Table 3]: on average females (mean *SD*) reported lower arousal values than males (mean *SD*). Also the interaction between age and gender [*F*_(2, 11, 741)_ = 4.52, *p* = 0.01] were significant. *Post-hoc* comparisons highlighted a significant difference [*F*_(1, 2, 909)_ = 11.27, *p* < 0.0001] between females 16–19 (mean = 4.79, *SD* = 2.62) and males 16–19 (mean = 5.12, *SD* = 2.64), with females evaluating the verbs as less arousing. There were not significant differences between females and males of the other age ranges.

### Emotional valence

**Figure 3:**
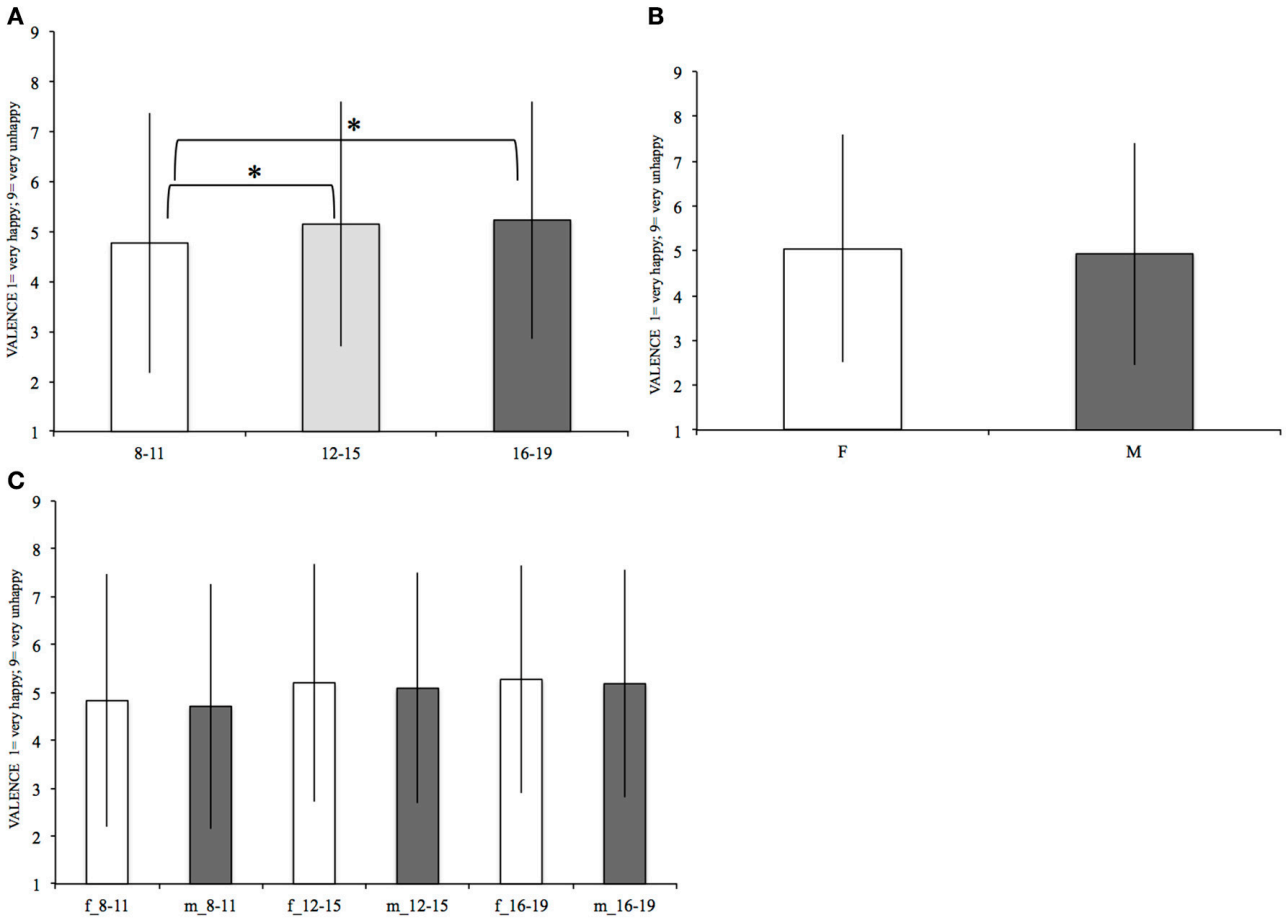
**Mean valence within each age range (A)**, within females (F) and males (M) **(B)**, and within females and males of each age range **(C)**. The significant differences, surviving the Bonferroni correction, are marked (“^*^”). Bars denote standard deviations.

Emotional valence's mean values for females and males in each age range are reported in Figure 3 and Table 3.

There was a significant effect of age on the Emotional Valence ratings [*F*_(2, 11, 741)_ = 29.75, *p* < 0.001]. *Post-hoc* contrasts revealed that the 8–11 group judged the verbs generally as having a more positive Valence (mean = 4.77, *SD* = 2.6) than both the 12–15 [*F*_(1, 8, 832)_ = 33.83, *p* < 0.001, mean = 5.15, *SD* = 2.45] and 16–19 [*F*_(1, 8, 403)_ = 46.82, *p* < 0.001, mean = 5.23, *SD* = 2.38] groups. The difference between 12–15 and 16–19 was not significant (see Table 3).

### Imageability

**Figure 4:**
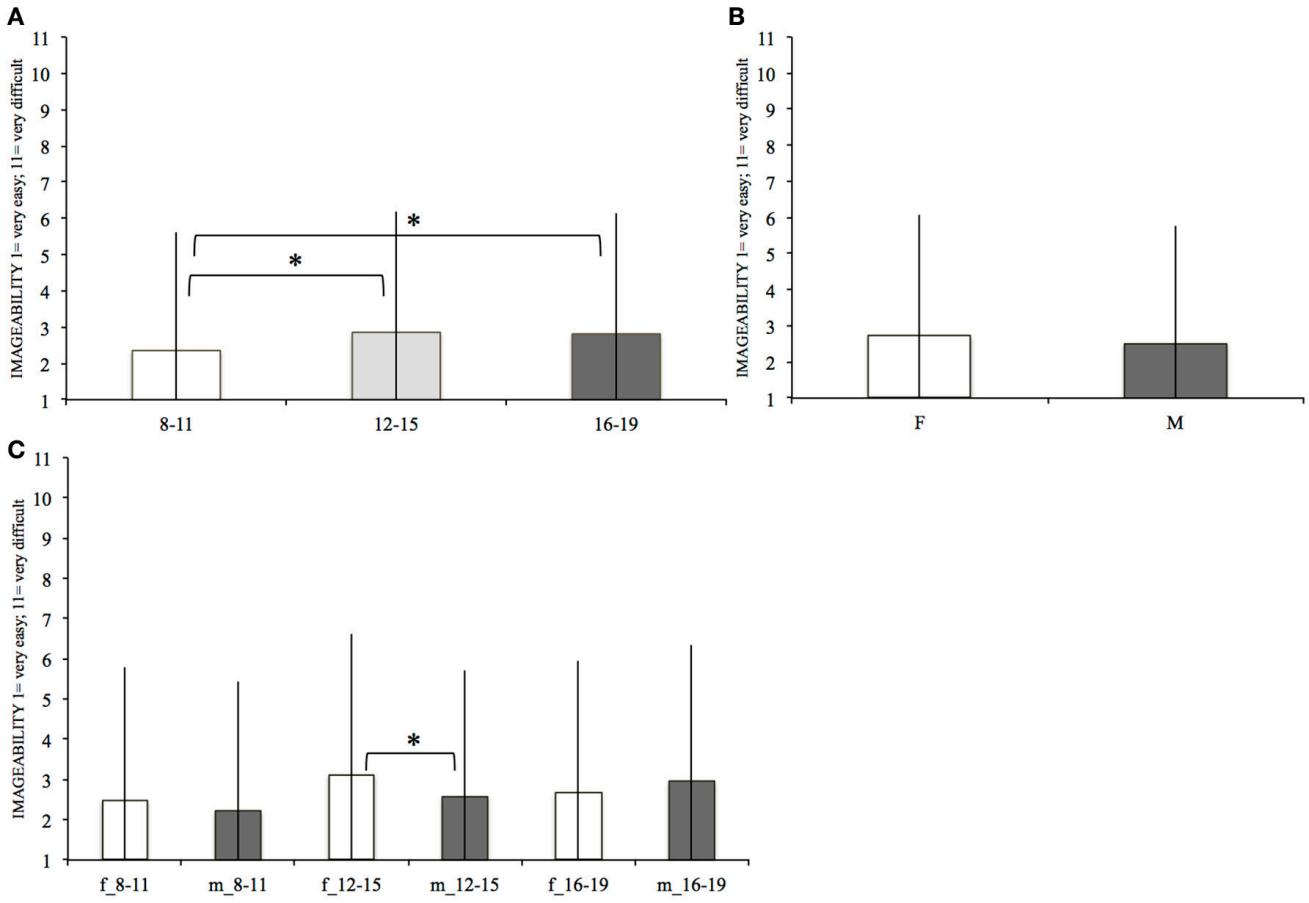
**Mean imageability within each age range (A)** within females (F) and males (M) **(B)** and within females and males of each age range **(C)**. The significant differences, surviving the Bonferroni correction, are marked (“^*^”). Bars denote standard deviations.

Imageability's mean values for females and males in each age range are represented in Figure 4.

There was a significant effect of age on Imageability ratings [*F*_(2, 11, 741)_ = 21.38, *p* < 0.001]. *Post-hoc* comparisons showed that the younger group (8–11) considered the verbs easier to be imagined (mean = 2.35, *SD* = 3.27) than the 12–15 [mean = 2.86, *SD* = 3.34; *F*_(1, 8, 832)_ = 36.27; *p* < 0.001] and the 16–19 [mean = 2.81, *SD* = 3.33; *F*_(1, 8, 403)_ = 23.95; *p* < 0.001] groups. Participants in the age ranges 12–15 and 15–19 did not show a significant difference between each other (*p* > 0.1).

Gender by age interaction was significant [*F*_(2, 11, 741)_ = 13.78; *p* < 0.001]. *Post-hoc* comparisons revealed that within the 12–15 age range [*F*_(1, 3, 338)_ = 24.58, *p* < 0.001] males (mean = 2.57, *SD* = 3.15) judged the verbs as easier to be imagined than females (mean = 3.12, *SD* = 3.49).

### Age of acquisition

**Figure 5:**
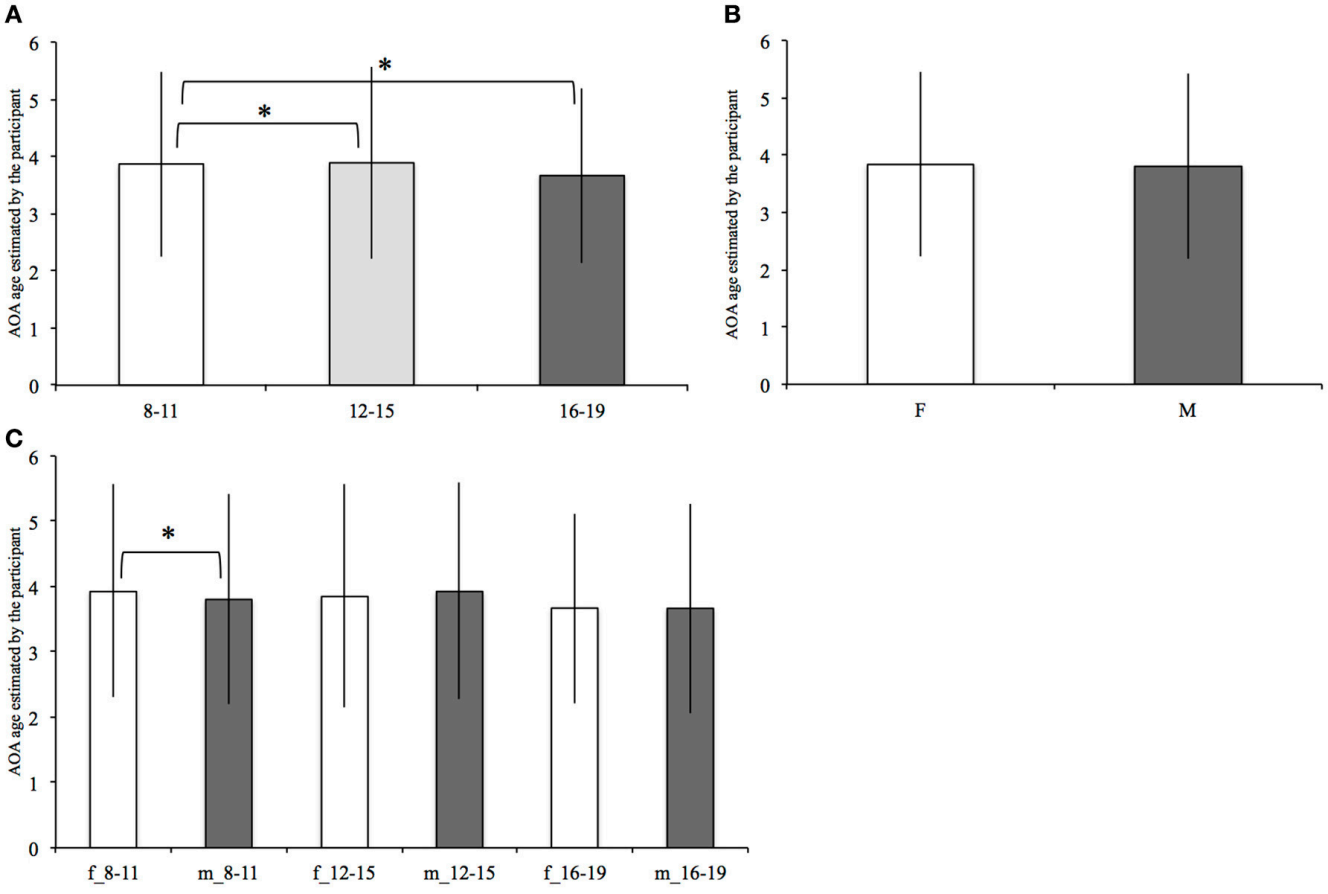
**Mean AOA within each age range (A)**, within females (F) and males (M) **(B)**, and within females and males of each age range **(C)**. The significant differences, surviving the Bonferroni correction, are marked (“^*^”). Bars denote standard deviations.

Mean age of acquisition (AOA) values for females and males in each age range are represented in Figure 5.The analysis revealed a main effect of age [*F*_(2, 11, 741)_ = 20.28, *p* < 0.001]. The *post-hoc* comparisons showed that the 8–11 (mean = 3.87, *SD* = 1.62) and 12–15 (mean = 3.89, *SD* = 1.68) did not differ for AOA, while the 16–19 years old reported on average an earlier AOA (mean 3.67, *SD* = 1.53) than both 8–11[*F*_(1, 8, 403)_ = 35.47, *p* < 0.001] and 12–15 [*F*_(1, 6, 251)_ = 30.38, *P* < 0.001].

### Regressions

Emotion relatedness and motor relatedness predicting arousal, emotional valence, imageability, and AOA.

**Figure 6:**
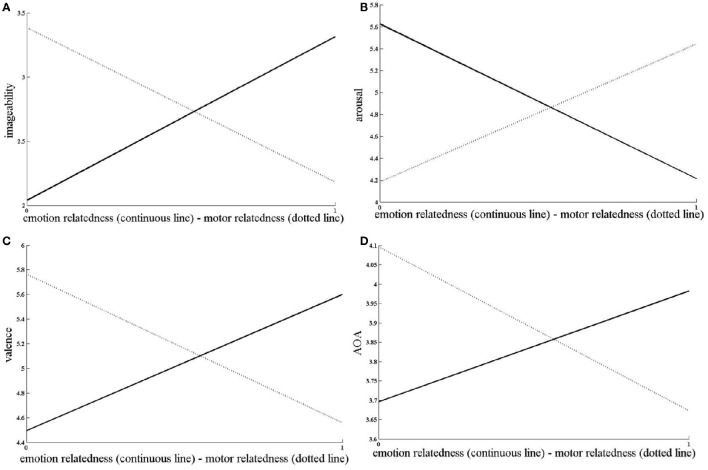
**Emotion relatedness (continuous line) and motor relatedness (dotted line) predicting imageability (1 = very easy; 11 = very difficult) (A)**, arousal (1 = very calm; 9 = very aroused) **(B)**, valence (1 = very happy, 9 = very unhappy) **(C)** and age of acquisition (AOA; age estimated by the participant) **(D)**.

Imageability predicted arousal, valence, and AOA, as well as emotion and motor relatedness (all *p* < 0.001—see Table 3). The same results were obtained when the regressions were run within each age range (i.e., 8–11; 12–15; 16–19) with either emotion or motor relatedness judgments as predictors. The Regressions' results are summarized in Table 4 and graphically represented in Figure 6.

**Table 4:** **Emotion and Motor relatedness predicting arousal, emotional valence, imageability, and AOA; imageabilitiy predicting arousal, emotional valence, and AOA; Imageability predicting Emotion and Motor relatedness**.

	**Arousal**		**Valence**		**Imageability**		**AOA**	
**df (1, 11.745)**	***f***	***p***	***f***	***p***	***f***	***p***	***f***	***p***
Emotion relatedness	821.65	<0.001	562.37	<0.001	423.37	<0.001	99.58	<0.001
Motor relatedness	595.73	<0.001	650.19	<0.001	360.85	<0.001	192.57	<0.001
Imageability	82.6	<0.001	105.26	<0.001			64.64	<0.001
	**Emotion relatedness**		**Motor relatedness**					
Imageability	43.55	<0.001	39.68	<0.001				

### Correlations

All the indexes proved to be all correlated between each other (all *p* < 0.001—see Table 5).

**Table 5:** **Correlations–Emotion and Motor relatedness, arousal, emotional valence, imageability, and AOA**.

		**AOA**	**Arousal**	**Valence**	**Imageability**	**Motor-relatedness**	**Emotion-relatedness**
AOA	*r*	1.00	−0.140^**^	0.134^**^	0.210^**^	−0.126^**^	0.088^**^
	*p*		**0.00**	**0.00**	**0.00**	**0.00**	**0.00**
Arousal	*r*		1.00	−0.485^**^	−0.263^**^	0.220^**^	−0.256^**^
	*p*			**0.00**	**0.00**	**0.00**	**0.00**
Valence	*r*			1.00	0.301^**^	−0.231^**^	0.219^**^
	*p*				**0.00**	**0.00**	**0.00**
Imaginability	*r*				1.00	−0.174^**^	0.191^**^
	*p*					**0.00**	**0.00**
Motor-relatedness	*r*					1.00	−0.715^**^
Motor-relatedness	*p*						**0.00**
Motor-relatedness	*r*						1.00
	*p*						

The r values indicating significant correlations are marked with the “**” and the significant p values are in bold.

Interestingly age at which participants reckon they learned the meaning of the verb correlated negatively with attribution of motor relatedness meaning that motor-related verbs were learned early and positively with attribution of emotion relatedness meaning that motor-related verbs were learned late.

Arousal correlated positively with attribution of motor relatedness, and negatively correlated with attribution of emotion relatedness meaning that higher physiologic activation increased when processing the verbs as motor related and decreased when processing the verbs as emotion related.

Valence correlated negatively with attribution of motor relatedness and positively correlated with attribution of emotion relatedness meaning that happiness increased when processing the verbs as motor related and decreased when processing the verbs as emotion related.

Lastly, motor relatedness negatively correlated with imageability while there was a positive correlation between the emotion relatedness and imageability (*p* < 0.001). So, attributions of motor relatedness correlated to lower imageability scores suggesting that the motor verbs were easier to be imagined. Instead, attribution of emotion relatedness correlated with higher imageablity scores, thus were harder to be imagined. In addition, imageability positively correlated with valence, thus attribution of positive valence correlated with higher imageablity scores, i.e., with verbs that were harder to be imagined. Imageability negatively correlated with arousal, meaning that verbs that were harder to be imagined elicited low arousal levels.

## Discussion

The aim of our study was to investigate the knowledge that children and adolescents had of a set of 140 Italian verbs and measure how they process them according to their relation with an “emotional” or “motor” concept.

The participants claimed to be familiar with all the verbs, even at the younger age. However, the full awareness of the concepts conveyed by the verbs develops, as indicated by their judgments on the emotion or motor relatedness, appeared to be influenced by their age and, in some cases, also by their gender.

### Emotion and motor relatedness

The analyses showed that children of 8–11 years tended to judge the verbs as “Emotional” less frequently than both the 12–15 and 16–19 years old children. Although children can have a certain awareness of basic emotions by 3–4 months, as indicated by Zieber et al. (2014), or even use some emotional labels by 28 months of age (Reilly et al., 1990), it appears to take far more time to reach a real knowledge of the emotional relatedness of verbs.

Moreover, contrarily to what reported by Baron-Cohen et al. (2010) for the emotional words, the present study shows that the development of the knowledge of the emotional verbs extends after the 11th year of age. This fit with the idea of a delay in the acquisition of the ability to use mental state verbs, apparently also with respect to emotional words (Barak et al., 2012). It is also possible that the delay in emotional (mental) verbs acquisition is due to the fact that they require mental scene construction, a process that may prove difficult for younger children. Our data do not allow for drawing conclusions on this point that should be addressed in future studies.

Beside the age, there was an effect of the children's gender, with females generally more prone to consider the verbs as emotional than males, and this difference was significant within the 8–11 age range only, while there were no differences between males and females within the 12–15 and 16–18 age ranges. From this pattern of results, it seems that the gender influences the emotional knowledge differently at different age. Taken together the main effect of gender and the gender by age interaction suggest that the knowledge of the emotional concepts related to verbs develops at different pace within females and males. It may reflect the dominant cultural environment attitude that wants the girls emotional and prone to positive feelings. Alternatively, this can be due to an innate predisposition. As already pointed out above, the questions concerning the genetic and/or environmental pressures on the girls' emotional sphere should be addressed in future studies, also because of the implications they can have on common view of girls as mainly driven by emotions, if compared to males that are seen as more practical and rational. Moreover, as already stated above, due to the effect of gender on different aspects of the verbs' knowledge, the future investigations should use groups of participants with homogeneous numbers of males and females.

Concerning the motor relatedness, a main effect of the age on participants' judgments emerged. The effect was driven by the younger children of 8–11 years who tended to judge the verbs as “Motor” more frequently than the 12–15 years old children. Instead, the 12–15 years old rated the verbs as motor as frequently as the 16–19. Various authors proposed that the semantic knowledge of all types word and verbs, but also objects and even emotions are grounded into motor representations, or are embodied (Kiefer and Pulvermüller, 2012; Moseley et al., 2012). However, from our data it seems that the motor content is not equally crucial at different ages, as our older participants judged the verbs as motor less frequently. Instead our results are more in line with a hypothesis that sees children particularly attracted by action contents (see below the effect of the measured indices). Then, growing up, children become gradually able to master more and more complex language structures (but also more complex aspects of the reality), such as the ones that indicate the presence of abstract concepts, feelings, and inner states. While this developmental change takes place the children and adolescents' attention becomes more easily cued by those complex structures and driven toward the emotional meaning conveyed by the verbs, as suggested by Barak et al. (2012).

Crucially, there is not an effect of the gender. This may suggest a general and innate tendency to give attention to the motor content of the verbs, which however seems to loose its prominence as soon as the children learn other aspects that characterize more peculiar aspects of the verbs' meaning (i.e., the emotional content).

The nature of the influence of age, gender and their interaction on the development of emotional and motor concepts related to verbs is worthy to be clarified in future studies that will investigate its relation to a genetic predisposition and/or to the environmental differential pressures on children of the two genders. Moreover, as the gender proved to have an influence especially on the knowledge of the emotional meaning of the verbs, future studies on this topic should balance the number of males and females participants across their sample's groups. Also more neuroimaging, but also behavioral, studies are required to better understand the verbs' meaning representation in the brain and, more specifically, to test the hypothesis that as long as the emotional meaning of a verb is acquired it is this representation, and not the motor, to be preferentially activated when the verb is presented, with the task's context probably giving an important cue to such activation.

### Arousal, emotional valence, imageability, and AOA

We investigated the possible main effects of age and gender and their interaction in influencing the participants' estimates of valence, arousal, imageability and age of acquisition (AOA).

The analyses revealed a significant main effect of age on AOA, valence, and imageability. Also, there was a significant interaction of age and gender for the imageability index.

On the significant main effect of age on AOA was mainly driven by the participants in the 16–19 age range who estimated to have acquired the verbs at a younger age than both the 8–11 and the 12–15. The reason for this difference can be interpreted in terms of the 16–19 being more precise than the younger children. Previous studies on adults indicate that AOA is an accurate estimate of the true age at which the vocabulary is actually acquired (see Bird et al., 2001). Our data show that the ability to do such estimate evolves over time through childhood to late adolescence. AOA positively correlated with attribution of emotion relatedness meaning that full awareness on the temporal aspect of acquiring emotion-related verbs occurs later (and negatively with attribution of motor relatedness).

Surprisingly, there was not an effect of age on arousal, meaning that all the physiological activation (arousal) was comparable across different ages. Instead there was an effect of gender on arousal and an interaction between gender and age. Particularly, there was a difference between females and males in the 16–19 age range, with females reporting to be less activated by the content described by the verbs. It is possible that females are less activated due to developmental changes occurring earlier in females than males. Further studies, involving also older participants, may help to learn more about this point. As shown by the correlation analysis it increased when attributing motor relatedness to verbs and decreased when attributing emotion relatedness as shown by the correlation analysis. This indicates that the preference of the younger children for the motor content of the verbs may be due to an innate preference to give attention to actions, as suggested by Barak et al. (2012), and not to the fact that the younger are more aroused or less able to take their arousal levels under control.

The younger children (8–11 year range) gave to the verbs a more positive valence. So, on average and regardless by the type (emotional or motor), the concepts conveyed by the verbs elicited more happy feelings in the youngers. As discussed above, they also judged the verbs as motor more frequently. This may indicate that the younger children are more attracted by motor contents also because of the positive feelings they can evoke. Indeed the correlation analysis showed that positive valence ratings increased when processing the verbs as motor related and decreased when processing the verbs as emotion related. This point it is worthy of been further investigated even for the possible implications that this may have on the therapeutic practice. The older (12–15 and 16–19) were not significantly different meaning that after 12 years the valence attribution become stable.

Also, the younger children (8–11) stated that the verbs were easier to be imagined, if compared to the older children's judgments suggesting that at this age vividness estimation is still rough. Our result shows a developmental change in the imageablity estimates, however more studies are required to determine the nature of this change as well as the quality of the mental images at different ages.

Also, for the imageability index, it emerged a significant age by gender interaction. Indeed, especially the females in the middle age range (12–15) found the verbs more difficult to be imagined than the males of same age, although the available data do not allow drawing straightforward conclusions about this point. A more pronounced preference for abstract contents, reflected by more frequent emotional judgments, could have suggested a tendency to indulge into not concrete thoughts that are not easily imaginable.

Imageability predicted arousal, age of acquisition, emotion- and motor-relatedness estimations indicating that this index influences the way verbs are processed and was positively correlated to emotion relatedness indicating that such verbs were harder to be imagined (and negatively correlated to motor-related verbs). Overall these results indicate that it is neither simply a matter of motor and visual-relatedness driving the processing of the corresponding concepts, nor an effect of imageability alone. Rather, these data indicate that it is likely the combination or summation of the investigated indexes that modulates how young people are processing motor and emotion concepts. This result strongly indicate the effect of contextual modulation on the formation of motor and emotion concepts. Laslty, imageablity positively correlated with valence meaning that verbs receiving positive valence were also those that were hard to be imagined, and negatively correlated with arousal, meaning that verbs that were harder to be imagined elicited low physiological activation.

Future studies should investigate more closely the effect of the verb's motor or emotion relatedness on imageability, arousal, valence and AOA, possibly using a bigger verbs' set that will allow to divide the verbs into (big enough) groups according to the frequency with which they are judged as motor related, emotion related or both.

Also, it should be pointed out that the correlations between these measures imply that all the aspects and factors characterizing the semantic representations of verbs (or vocabulary in general) develop in parallel during childhood and adolescence. Thus, it is the case to consider them cautiously when selecting verbal items to be used in studies having a developmental perspective. Note that our study involved participant up to 19 years of age. So we cannot exclude further modifications of the semantic representation of emotional and motor verbs (or vocabulary in general) through adulthood and on in the elderly age. Extending further our study, in order to include participants from more age ranges, may be a worthy challenge for the next future.

## Conclusions

Consistently with other studies and observations (e.g., Reilly et al., 1990; Zieber et al., 2014), in the present investigation the participants demonstrated to know the emotional verbs since an early age. Indeed the “familiarity” index was almost at ceiling and the AOA, which is considered an accurate estimate of the real age at which words/verbs are acquired (see Bird et al., 2001), was quite low, although this seemed to be a basic knowledge.

Our study suggests that children become conscious of the real meaning conveyed by the verbs gradually, with this process continuing, at least, until the late adolescence/early adulthood.

The younger children assigned an emotional meaning to the verbs less frequently than the older. Imageability also was influenced by age, with the younger evaluating the verbs as easier to be imagined, indicating a rough imagination. Also, our results are in line with an hypothesis according to which the children develop the awareness of the emotional contents of verbs as soon as they can master the complex aspects of language and reality that cue their attention toward abstract elements, feelings, or inner states.

The consciousness of the emotional content of the verbs appeared to be influenced also by gender. This result may reflect an innate tendency of females to be more prone to emotion than males. However, the difference between females and males existed only children within the younger children aged 8–11. This suggests that the difference can be culturally determined. It is likely that females are expected to be more emotional and inclined to kindness and affability than males and so they more exposed to emotional language. More studies are worthy to be run to clarify this point. In any case we recommend balancing the participants' groups for gender when studying emotions and emotional (or mental state) verbs and words.

Finally, all the indexes measured in the present investigation (i.e., familiarity, motor judgment, emotion judgment, valence, arousal, imageability, and AOA) had a strong relation between each other. Crucially imageability predicted all the indexes measured in the present investigation. Further analyses showed that the correlation of imageability with motor and emotional judgments that had opposite directions, suggesting the existence of a differential relation of the two types of verbs with mental images. This suggests that motor related verbs might rely more on mental images having sensory representations while emotion related verbs elicit more abstract contents that can hardly evoke mental images. This result is not fully in agreement with the hypothesis of the embodied cognition theory according to which in adolescents and children emotion related concepts are treated as motor related concepts.

Finally, the relations between all the measures indicates the need of considering all those variables when using verbs as stimuli for experiments, especially in those involving indexes sensitive to small variations (e.g., reaction times or BOLD signal).

Overall, these results show that it is likely that a combination or summation of the investigated indexes modulates how young people are processing motor and emotion concepts. This result strongly indicates the effect of contextual modulation on the formation of motor and emotion concepts. Further studies, also on older age ranges (adults and elder) and across different languages and cultures.

## Author contributions

CB: Data analysis, report, interpretation, and discussion of the results, manuscript writing. BT: Experimental design, reviewing manuscript. MG: Participants' recruitment and data acquisition. SP: Participants' recruitment and data acquisition. FF: Experimental design, critical review. PB: Experimental design, critical review, research coordinator.

### Conflict of interest statement

The authors declare that the research was conducted in the absence of any commercial or financial relationships that could be construed as a potential conflict of interest.

## Footnotes

^1^Few more participants took part in the survey. However they questionnaire were discarded by default and were not entered in the dataset as <85% of the questions were answered.

^2^Emotional verbs may also involve somehow the body, however they did not denoted actual body action. Similarly, motor verbs may evoke some pleasant or unpleasant experience, although they did not describe typical emotions. The extent to which each verb was related to feelings and body activity was judged later by seven independent raters.

^3^The α level was adjusted according to 13 comparisons: i.e., 1 general analysis exploring the main effects, 3 *post-hoc* comparisons clarifying the effect of age and 9 disentangling the gender by age the interactions (i.e., 0.05/13 = 0.004). This allowed also being consistent with the α level adopted for the analyses on the emotional and motor judgments presented above.
