# Supplementary material for: Age-Dependent Changes of Thinking about Verbs
Source: Front Behav Neurosci. 2017 Mar 14;11:40. doi: 10.3389/fnbeh.2017.00040 (PMC5348498; doi:10.3389/fnbeh.2017.00040)
Supplement: Supplementary file 1 [file Table1.DOCX]

**Supplementary Table 1** **- percentages of the emotion and motor relatedness judgments**

|  | 1 a - age range -> 8-19 (overall sample) | | | | | | 1 b - age range -> 8-11 | | | | | | 1 c - age range -> 12-15 | | | | | | 1 d - age range -> 16-19 | | | | | |
| --- | --- | --- | --- | --- | --- | --- | --- | --- | --- | --- | --- | --- | --- | --- | --- | --- | --- | --- | --- | --- | --- | --- | --- | --- |
|  | EMOTION RELATEDNESS | | | MOTOR RELATEDNESS | | | EMOTION RELATEDNESS | | | MOTOR RELATEDNESS | | | EMOTION RELATEDNESS | | | MOTOR RELATEDNESS | | | EMOTION RELATEDNESS | | | MOTOR RELATEDNESS | | |
| *verbo* | *no %* | *yes %* | *agree %* | *no %* | *yes %* | *agree %* | *no %* | *yes %* | *agree %* | *no %* | *yes %* | *agree %* | *no %* | *yes %* | *agree %* | *no %* | *yes %* | *agree %* | *no %* | *yes %* | *agree %* | *no %* | *yes %* | *agree %* |
| abbottonarsi | 96,20% | 3,80% | 92,60% | 2,53% | 97,47% | 95,00% | 94,29% | 5,71% | 88,91% | 2,86% | 97,14% | 94,29% | 100,00% | 0,00% | 100,00% | 4,00% | 96,00% | 92,00% | 94,74% | 5,26% | 89,47% | 0,00% | 100,00% | 100,00% |
| afferrare | 95,00% | 5,00% | 90,38% | 0,00% | 100,00% | 100,00% | 100,00% | 0,00% | 100,00% | 0,00% | 100,00% | 100,00% | 92,59% | 7,41% | 85,75% | 0,00% | 100,00% | 100,00% | 89,47% | 10,53% | 80,12% | 0,00% | 100,00% | 100,00% |
| affettare | 90,79% | 9,21% | 83,05% | 10,53% | 89,47% | 80,91% | 88,10% | 11,90% | 78,51% | 7,14% | 92,86% | 86,41% | 90,00% | 10,00% | 81,05% | 20,00% | 80,00% | 66,32% | 100,00% | 0,00% | 100,00% | 7,14% | 92,86% | 85,71% |
| aggrapparsi | 88,24% | 11,76% | 78,99% | 0,00% | 100,00% | 100,00% | 97,44% | 2,56% | 94,87% | 0,00% | 100,00% | 100,00% | 87,50% | 12,50% | 77,17% | 0,00% | 100,00% | 100,00% | 72,73% | 27,27% | 58,44% | 0,00% | 100,00% | 100,00% |
| allacciarsi | 96,34% | 3,66% | 92,86% | 1,22% | 98,78% | 97,56% | 97,30% | 2,70% | 94,59% | 2,70% | 97,30% | 94,59% | 92,00% | 8,00% | 84,67% | 0,00% | 100,00% | 100,00% | 100,00% | 0,00% | 100,00% | 0,00% | 100,00% | 100,00% |
| amare | 1,22% | 98,78% | 97,56% | 84,15% | 15,85% | 72,99% | 2,70% | 97,30% | 94,59% | 86,49% | 13,51% | 75,98% | 0,00% | 100,00% | 100,00% | 84,00% | 16,00% | 72,00% | 0,00% | 100,00% | 100,00% | 80,00% | 20,00% | 66,32% |
| amarsi | 4,88% | 95,12% | 90,61% | 82,93% | 17,07% | 71,33% | 7,14% | 92,86% | 86,41% | 88,10% | 11,90% | 78,51% | 4,76% | 95,24% | 90,48% | 80,95% | 19,05% | 67,62% | 0,00% | 100,00% | 100,00% | 73,68% | 26,32% | 59,06% |
| ammalarsi | 46,60% | 53,40% | 49,74% | 44,66% | 55,34% | 50,09% | 50,94% | 49,06% | 49,06% | 49,06% | 50,94% | 49,06% | 40,74% | 59,26% | 49,86% | 44,44% | 55,56% | 48,72% | 43,48% | 56,52% | 48,62% | 34,78% | 65,22% | 52,57% |
| annodare | 96,08% | 3,92% | 92,39% | 2,94% | 97,06% | 94,23% | 94,12% | 5,88% | 88,71% | 3,92% | 96,08% | 92,31% | 96,30% | 3,70% | 92,59% | 3,70% | 96,30% | 92,59% | 100,00% | 0,00% | 100,00% | 0,00% | 100,00% | 100,00% |
| annoiarsi | 4,82% | 95,18% | 90,71% | 91,57% | 8,43% | 84,37% | 7,50% | 92,50% | 85,77% | 90,00% | 10,00% | 81,54% | 0,00% | 100,00% | 100,00% | 91,67% | 8,33% | 84,06% | 5,26% | 94,74% | 89,47% | 94,74% | 5,26% | 89,47% |
| apparecchiare | 92,59% | 7,41% | 86,11% | 3,70% | 96,30% | 92,78% | 90,63% | 9,38% | 82,46% | 3,13% | 96,88% | 93,75% | 87,50% | 12,50% | 77,17% | 8,33% | 91,67% | 84,06% | 100,00% | 0,00% | 100,00% | 0,00% | 100,00% | 100,00% |
| appendere | 91,57% | 8,43% | 84,37% | 8,43% | 91,57% | 84,37% | 87,18% | 12,82% | 77,06% | 12,82% | 87,18% | 77,06% | 95,83% | 4,17% | 91,67% | 0,00% | 100,00% | 100,00% | 95,00% | 5,00% | 90,00% | 10,00% | 90,00% | 81,05% |
| applaudire | 85,19% | 14,81% | 74,44% | 1,23% | 98,77% | 97,53% | 92,11% | 7,89% | 85,06% | 0,00% | 100,00% | 100,00% | 76,00% | 24,00% | 62,00% | 0,00% | 100,00% | 100,00% | 83,33% | 16,67% | 70,59% | 5,56% | 94,44% | 88,89% |
| arrabbiarsi | 6,10% | 93,90% | 88,41% | 67,07% | 32,93% | 55,28% | 12,50% | 87,50% | 77,42% | 62,50% | 37,50% | 51,61% | 0,00% | 100,00% | 100,00% | 68,00% | 32,00% | 54,67% | 4,00% | 96,00% | 92,00% | 72,00% | 28,00% | 58,00% |
| avvitare | 98,65% | 1,35% | 97,30% | 2,70% | 97,30% | 94,67% | 96,97% | 3,03% | 93,94% | 3,03% | 96,97% | 93,94% | 100,00% | 0,00% | 100,00% | 0,00% | 100,00% | 100,00% | 100,00% | 0,00% | 100,00% | 5,88% | 94,12% | 88,24% |
| avvolgersi | 87,34% | 12,66% | 77,60% | 5,06% | 94,94% | 90,26% | 87,10% | 12,90% | 76,77% | 9,68% | 90,32% | 81,94% | 87,50% | 12,50% | 77,17% | 4,17% | 95,83% | 91,67% | 87,50% | 12,50% | 77,17% | 0,00% | 100,00% | 100,00% |
| bussare | 98,78% | 1,22% | 97,56% | 1,22% | 98,78% | 97,56% | 97,37% | 2,63% | 94,74% | 2,63% | 97,37% | 94,74% | 100,00% | 0,00% | 100,00% | 0,00% | 100,00% | 100,00% | 100,00% | 0,00% | 100,00% | 0,00% | 100,00% | 100,00% |
| calmare | 22,62% | 77,38% | 64,57% | 63,10% | 36,90% | 52,87% | 39,47% | 60,53% | 50,92% | 52,63% | 47,37% | 48,79% | 7,41% | 92,59% | 85,75% | 81,48% | 18,52% | 68,66% | 10,53% | 89,47% | 80,12% | 57,89% | 42,11% | 48,54% |
| calmarsi | 16,49% | 83,51% | 72,16% | 65,98% | 34,02% | 54,64% | 23,53% | 76,47% | 63,29% | 60,78% | 39,22% | 51,37% | 13,64% | 86,36% | 75,32% | 68,18% | 31,82% | 54,55% | 4,17% | 95,83% | 91,67% | 75,00% | 25,00% | 60,87% |
| cancellare | 95,19% | 4,81% | 90,76% | 0,96% | 99,04% | 98,08% | 98,15% | 1,85% | 96,30% | 0,00% | 100,00% | 100,00% | 88,46% | 11,54% | 78,77% | 3,85% | 96,15% | 92,31% | 95,83% | 4,17% | 91,67% | 0,00% | 100,00% | 100,00% |
| confessare | 25,88% | 74,12% | 61,18% | 70,59% | 29,41% | 57,98% | 31,43% | 68,57% | 55,63% | 60,00% | 40,00% | 50,59% | 28,00% | 72,00% | 58,00% | 80,00% | 20,00% | 66,67% | 16,00% | 84,00% | 72,00% | 76,00% | 24,00% | 62,00% |
| confondersi | 14,46% | 85,54% | 74,96% | 80,72% | 19,28% | 68,50% | 20,51% | 79,49% | 66,53% | 74,36% | 25,64% | 60,86% | 12,00% | 88,00% | 78,00% | 80,00% | 20,00% | 66,67% | 5,26% | 94,74% | 89,47% | 94,74% | 5,26% | 89,47% |
| controllare | 63,53% | 36,47% | 53,11% | 35,29% | 64,71% | 53,78% | 75,00% | 25,00% | 61,54% | 40,00% | 60,00% | 50,77% | 61,54% | 38,46% | 50,77% | 38,46% | 61,54% | 50,77% | 42,11% | 57,89% | 48,54% | 21,05% | 78,95% | 64,91% |
| controllarsi | 37,18% | 62,82% | 52,68% | 33,33% | 66,67% | 54,98% | 44,74% | 55,26% | 49,22% | 28,95% | 71,05% | 57,75% | 38,10% | 61,90% | 50,48% | 47,62% | 52,38% | 47,62% | 21,05% | 78,95% | 64,91% | 26,32% | 73,68% | 59,06% |
| credere | 6,02% | 93,98% | 88,54% | 90,36% | 9,64% | 82,37% | 9,76% | 90,24% | 81,95% | 92,68% | 7,32% | 86,10% | 0,00% | 100,00% | 100,00% | 87,50% | 12,50% | 77,17% | 5,56% | 94,44% | 88,89% | 88,89% | 11,11% | 79,08% |
| decidere | 16,28% | 83,72% | 72,42% | 80,23% | 19,77% | 67,91% | 22,73% | 77,27% | 64,06% | 84,09% | 15,91% | 72,62% | 15,79% | 84,21% | 71,93% | 89,47% | 10,53% | 80,12% | 4,35% | 95,65% | 91,30% | 65,22% | 34,78% | 52,57% |
| deludere | 8,54% | 91,46% | 84,19% | 85,37% | 14,63% | 74,71% | 13,16% | 86,84% | 76,53% | 84,21% | 15,79% | 72,69% | 4,35% | 95,65% | 91,30% | 82,61% | 17,39% | 69,96% | 4,76% | 95,24% | 90,48% | 90,48% | 9,52% | 81,90% |
| deprimersi | 6,15% | 93,85% | 88,27% | 86,15% | 13,85% | 75,77% | 15,38% | 84,62% | 72,92% | 84,62% | 15,38% | 72,92% | 0,00% | 100,00% | 100,00% | 85,00% | 15,00% | 73,16% | 0,00% | 100,00% | 100,00% | 89,47% | 10,53% | 80,12% |
| desiderare | 4,90% | 95,10% | 90,58% | 90,20% | 9,80% | 82,14% | 5,88% | 94,12% | 88,71% | 86,27% | 13,73% | 75,84% | 7,41% | 92,59% | 85,75% | 92,59% | 7,41% | 85,75% | 0,00% | 100,00% | 100,00% | 95,83% | 4,17% | 91,67% |
| dimenticare | 16,47% | 83,53% | 72,16% | 82,35% | 17,65% | 70,59% | 19,44% | 80,56% | 67,78% | 77,78% | 22,22% | 64,44% | 16,67% | 83,33% | 71,01% | 83,33% | 16,67% | 71,01% | 12,00% | 88,00% | 78,00% | 88,00% | 12,00% | 78,00% |
| dipingere | 75,96% | 24,04% | 63,13% | 2,88% | 97,12% | 94,34% | 80,00% | 20,00% | 67,41% | 3,64% | 96,36% | 92,86% | 74,07% | 25,93% | 60,11% | 3,70% | 96,30% | 92,59% | 68,18% | 31,82% | 54,55% | 0,00% | 100,00% | 100,00% |
| disegnare | 84,93% | 15,07% | 74,05% | 4,11% | 95,89% | 92,01% | 93,75% | 6,25% | 87,90% | 3,13% | 96,88% | 93,75% | 72,73% | 27,27% | 58,44% | 0,00% | 100,00% | 100,00% | 84,21% | 15,79% | 71,93% | 10,53% | 89,47% | 80,12% |
| dispiacersi | 1,25% | 98,75% | 97,50% | 88,75% | 11,25% | 79,78% | 2,33% | 97,67% | 95,35% | 86,05% | 13,95% | 75,42% | 0,00% | 100,00% | 100,00% | 88,24% | 11,76% | 77,94% | 0,00% | 100,00% | 100,00% | 95,00% | 5,00% | 90,00% |
| fallire | 21,69% | 78,31% | 65,62% | 75,90% | 24,10% | 62,97% | 41,18% | 58,82% | 50,09% | 55,88% | 44,12% | 49,20% | 8,33% | 91,67% | 84,06% | 87,50% | 12,50% | 77,17% | 8,00% | 92,00% | 84,67% | 92,00% | 8,00% | 84,67% |
| fingere | 33,75% | 66,25% | 54,72% | 57,50% | 42,50% | 50,51% | 55,88% | 44,12% | 49,20% | 50,00% | 50,00% | 48,48% | 19,23% | 80,77% | 67,69% | 69,23% | 30,77% | 55,69% | 15,00% | 85,00% | 73,16% | 55,00% | 45,00% | 47,89% |
| fotografarsi | 80,00% | 20,00% | 67,59% | 13,75% | 86,25% | 75,98% | 74,47% | 25,53% | 61,15% | 4,26% | 95,74% | 91,67% | 95,00% | 5,00% | 90,00% | 30,00% | 70,00% | 55,79% | 76,92% | 23,08% | 61,54% | 23,08% | 76,92% | 61,54% |
| gettare | 97,59% | 2,41% | 95,24% | 0,00% | 100,00% | 100,00% | 97,30% | 2,70% | 94,59% | 0,00% | 100,00% | 100,00% | 96,15% | 3,85% | 92,31% | 0,00% | 100,00% | 100,00% | 100,00% | 0,00% | 100,00% | 0,00% | 100,00% | 100,00% |
| graffiarsi | 88,12% | 11,88% | 78,85% | 1,98% | 98,02% | 96,08% | 88,89% | 11,11% | 79,87% | 0,00% | 100,00% | 100,00% | 86,96% | 13,04% | 76,28% | 4,35% | 95,65% | 91,30% | 87,50% | 12,50% | 77,17% | 4,17% | 95,83% | 91,67% |
| grattarsi | 98,70% | 1,30% | 97,40% | 0,00% | 100,00% | 100,00% | 96,77% | 3,23% | 93,55% | 0,00% | 100,00% | 100,00% | 100,00% | 0,00% | 100,00% | 0,00% | 100,00% | 100,00% | 100,00% | 0,00% | 100,00% | 0,00% | 100,00% | 100,00% |
| grattugiare | 96,39% | 3,61% | 92,95% | 8,43% | 91,57% | 84,37% | 95,56% | 4,44% | 91,31% | 11,11% | 88,89% | 79,80% | 95,00% | 5,00% | 90,00% | 10,00% | 90,00% | 81,05% | 100,00% | 0,00% | 100,00% | 0,00% | 100,00% | 100,00% |
| guarire | 61,73% | 38,27% | 52,16% | 27,16% | 72,84% | 59,94% | 65,63% | 34,38% | 53,43% | 25,00% | 75,00% | 61,29% | 70,83% | 29,17% | 56,88% | 16,67% | 83,33% | 71,01% | 48,00% | 52,00% | 48,00% | 40,00% | 60,00% | 50,00% |
| impaurirsi | 4,04% | 95,96% | 92,17% | 74,75% | 25,25% | 61,86% | 7,69% | 92,31% | 85,52% | 69,23% | 30,77% | 56,56% | 0,00% | 100,00% | 100,00% | 84,62% | 15,38% | 72,92% | 0,00% | 100,00% | 100,00% | 76,19% | 23,81% | 61,90% |
| impazzire | 12,35% | 87,65% | 78,09% | 74,07% | 25,93% | 61,11% | 22,22% | 77,78% | 64,44% | 77,78% | 22,22% | 64,44% | 8,33% | 91,67% | 84,06% | 66,67% | 33,33% | 53,62% | 0,00% | 100,00% | 100,00% | 76,19% | 23,81% | 61,90% |
| impressionarsi | 1,27% | 98,73% | 97,47% | 89,87% | 10,13% | 81,56% | 2,78% | 97,22% | 94,44% | 94,44% | 5,56% | 89,21% | 0,00% | 100,00% | 100,00% | 88,00% | 12,00% | 78,00% | 0,00% | 100,00% | 100,00% | 83,33% | 16,67% | 70,59% |
| inchiodare | 93,33% | 6,67% | 87,39% | 6,67% | 93,33% | 87,39% | 95,12% | 4,88% | 90,49% | 0,00% | 100,00% | 100,00% | 94,74% | 5,26% | 89,47% | 21,05% | 78,95% | 64,91% | 86,67% | 13,33% | 75,24% | 6,67% | 93,33% | 86,67% |
| incollarsi | 87,32% | 12,68% | 77,55% | 11,27% | 88,73% | 79,72% | 87,50% | 12,50% | 77,56% | 5,00% | 95,00% | 90,26% | 84,21% | 15,79% | 71,93% | 26,32% | 73,68% | 59,06% | 91,67% | 8,33% | 83,33% | 8,33% | 91,67% | 83,33% |
| indicarsi | 92,71% | 7,29% | 86,34% | 1,04% | 98,96% | 97,92% | 91,11% | 8,89% | 83,43% | 0,00% | 100,00% | 100,00% | 96,30% | 3,70% | 92,59% | 0,00% | 100,00% | 100,00% | 91,67% | 8,33% | 84,06% | 4,17% | 95,83% | 91,67% |
| infilare | 93,14% | 6,86% | 87,09% | 3,92% | 96,08% | 92,39% | 96,36% | 3,64% | 92,86% | 5,45% | 94,55% | 89,49% | 87,50% | 12,50% | 77,17% | 4,17% | 95,83% | 91,67% | 91,30% | 8,70% | 83,40% | 0,00% | 100,00% | 100,00% |
| ingelosirsi | 1,32% | 98,68% | 97,37% | 90,79% | 9,21% | 83,05% | 2,94% | 97,06% | 94,12% | 88,24% | 11,76% | 78,61% | 0,00% | 100,00% | 100,00% | 90,91% | 9,09% | 82,68% | 0,00% | 100,00% | 100,00% | 95,00% | 5,00% | 90,00% |
| insultare | 30,86% | 69,14% | 56,79% | 51,85% | 48,15% | 49,44% | 40,63% | 59,38% | 50,20% | 46,88% | 53,13% | 48,59% | 30,43% | 69,57% | 55,73% | 60,87% | 39,13% | 50,20% | 19,23% | 80,77% | 67,69% | 50,00% | 50,00% | 48,00% |
| intrecciarsi | 78,87% | 21,13% | 66,20% | 12,68% | 87,32% | 77,55% | 82,76% | 17,24% | 70,44% | 17,24% | 82,76% | 70,44% | 71,43% | 28,57% | 57,14% | 9,52% | 90,48% | 81,90% | 80,95% | 19,05% | 67,62% | 9,52% | 90,48% | 81,90% |
| intristirsi | 3,80% | 96,20% | 92,60% | 87,34% | 12,66% | 77,60% | 3,33% | 96,67% | 93,33% | 90,00% | 10,00% | 81,38% | 0,00% | 100,00% | 100,00% | 87,50% | 12,50% | 77,17% | 8,00% | 92,00% | 84,67% | 84,00% | 16,00% | 72,00% |
| invidiare | 3,75% | 96,25% | 92,69% | 92,50% | 7,50% | 85,95% | 6,25% | 93,75% | 87,90% | 90,63% | 9,38% | 82,46% | 4,35% | 95,65% | 91,30% | 95,65% | 4,35% | 91,30% | 0,00% | 100,00% | 100,00% | 92,00% | 8,00% | 84,67% |
| irritarsi | 28,38% | 71,62% | 58,79% | 59,46% | 40,54% | 51,13% | 51,72% | 48,28% | 48,28% | 48,28% | 51,72% | 48,28% | 20,83% | 79,17% | 65,58% | 62,50% | 37,50% | 51,09% | 4,76% | 95,24% | 90,48% | 71,43% | 28,57% | 57,14% |
| lanciare | 97,67% | 2,33% | 95,40% | 0,00% | 100,00% | 100,00% | 97,50% | 2,50% | 95,00% | 0,00% | 100,00% | 100,00% | 96,30% | 3,70% | 92,59% | 0,00% | 100,00% | 100,00% | 100,00% | 0,00% | 100,00% | 0,00% | 100,00% | 100,00% |
| lavarsi | 94,12% | 5,88% | 88,80% | 1,18% | 98,82% | 97,65% | 97,62% | 2,38% | 95,24% | 0,00% | 100,00% | 100,00% | 95,83% | 4,17% | 91,67% | 0,00% | 100,00% | 100,00% | 84,21% | 15,79% | 71,93% | 5,26% | 94,74% | 89,47% |
| legare | 88,37% | 11,63% | 79,21% | 8,14% | 91,86% | 84,87% | 94,44% | 5,56% | 89,21% | 11,11% | 88,89% | 79,68% | 82,61% | 17,39% | 69,96% | 8,70% | 91,30% | 83,40% | 85,19% | 14,81% | 73,79% | 3,70% | 96,30% | 92,59% |
| maledire | 19,75% | 80,25% | 67,90% | 74,07% | 25,93% | 61,11% | 36,36% | 63,64% | 52,27% | 63,64% | 36,36% | 52,27% | 10,71% | 89,29% | 80,16% | 82,14% | 17,86% | 69,58% | 5,00% | 95,00% | 90,00% | 80,00% | 20,00% | 66,32% |
| martellare | 94,94% | 5,06% | 90,26% | 5,06% | 94,94% | 90,26% | 94,12% | 5,88% | 88,59% | 5,88% | 94,12% | 88,59% | 100,00% | 0,00% | 100,00% | 0,00% | 100,00% | 100,00% | 90,48% | 9,52% | 81,90% | 9,52% | 90,48% | 81,90% |
| mentire | 17,72% | 82,28% | 70,46% | 78,48% | 21,52% | 65,79% | 21,05% | 78,95% | 65,86% | 78,95% | 21,05% | 65,86% | 9,09% | 90,91% | 82,68% | 72,73% | 27,27% | 58,44% | 21,05% | 78,95% | 64,91% | 84,21% | 15,79% | 71,93% |
| meritare | 29,63% | 70,37% | 57,78% | 70,37% | 29,63% | 57,78% | 42,50% | 57,50% | 49,87% | 55,00% | 45,00% | 49,23% | 23,81% | 76,19% | 61,90% | 85,71% | 14,29% | 74,29% | 10,00% | 90,00% | 81,05% | 85,00% | 15,00% | 73,16% |
| mescolare | 96,30% | 3,70% | 92,78% | 1,23% | 98,77% | 97,53% | 97,44% | 2,56% | 94,87% | 0,00% | 100,00% | 100,00% | 95,83% | 4,17% | 91,67% | 4,17% | 95,83% | 91,67% | 94,44% | 5,56% | 88,89% | 0,00% | 100,00% | 100,00% |
| morire | 36,36% | 63,64% | 53,19% | 37,50% | 62,50% | 52,59% | 37,78% | 62,22% | 51,92% | 42,22% | 57,78% | 50,10% | 30,00% | 70,00% | 55,79% | 60,00% | 40,00% | 49,47% | 39,13% | 60,87% | 50,20% | 8,70% | 91,30% | 83,40% |
| obbedire | 30,00% | 70,00% | 57,47% | 51,25% | 48,75% | 49,40% | 34,29% | 65,71% | 53,61% | 54,29% | 45,71% | 48,91% | 41,67% | 58,33% | 49,28% | 37,50% | 62,50% | 51,09% | 9,52% | 90,48% | 81,90% | 61,90% | 38,10% | 50,48% |
| odiare | 1,18% | 98,82% | 97,65% | 96,47% | 3,53% | 93,11% | 2,63% | 97,37% | 94,74% | 94,74% | 5,26% | 89,76% | 0,00% | 100,00% | 100,00% | 96,43% | 3,57% | 92,86% | 0,00% | 100,00% | 100,00% | 100,00% | 0,00% | 100,00% |
| odiarsi | 3,85% | 96,15% | 92,51% | 91,03% | 8,97% | 83,45% | 6,45% | 93,55% | 87,53% | 87,10% | 12,90% | 76,77% | 0,00% | 100,00% | 100,00% | 100,00% | 0,00% | 100,00% | 4,17% | 95,83% | 91,67% | 87,50% | 12,50% | 77,17% |
| peccare | 25,00% | 75,00% | 62,07% | 56,82% | 43,18% | 50,37% | 34,15% | 65,85% | 53,90% | 56,10% | 43,90% | 49,51% | 12,50% | 87,50% | 77,17% | 62,50% | 37,50% | 51,09% | 21,74% | 78,26% | 64,43% | 52,17% | 47,83% | 47,83% |
| penare | 31,82% | 68,18% | 55,94% | 71,21% | 28,79% | 58,37% | 41,94% | 58,06% | 49,68% | 64,52% | 35,48% | 52,69% | 33,33% | 66,67% | 52,94% | 66,67% | 33,33% | 52,94% | 11,76% | 88,24% | 77,94% | 88,24% | 11,76% | 77,94% |
| pennellarsi | 84,78% | 15,22% | 73,91% | 6,52% | 93,48% | 87,67% | 79,59% | 20,41% | 66,84% | 8,16% | 91,84% | 84,69% | 90,48% | 9,52% | 81,90% | 4,76% | 95,24% | 90,48% | 90,91% | 9,09% | 82,68% | 4,55% | 95,45% | 90,91% |
| perdere | 35,80% | 64,20% | 53,46% | 53,09% | 46,91% | 49,57% | 63,64% | 36,36% | 52,27% | 39,39% | 60,61% | 50,76% | 12,00% | 88,00% | 78,00% | 64,00% | 36,00% | 52,00% | 21,74% | 78,26% | 64,43% | 60,87% | 39,13% | 50,20% |
| perdonare | 16,25% | 83,75% | 72,44% | 72,50% | 27,50% | 59,62% | 28,95% | 71,05% | 57,75% | 60,53% | 39,47% | 50,92% | 0,00% | 100,00% | 100,00% | 81,82% | 18,18% | 68,83% | 10,00% | 90,00% | 81,05% | 85,00% | 15,00% | 73,16% |
| perdonarsi | 10,39% | 89,61% | 81,13% | 79,22% | 20,78% | 66,64% | 10,81% | 89,19% | 80,18% | 75,68% | 24,32% | 62,16% | 16,67% | 83,33% | 71,01% | 79,17% | 20,83% | 65,58% | 0,00% | 100,00% | 100,00% | 87,50% | 12,50% | 76,67% |
| perseguitare | 45,33% | 54,67% | 49,77% | 34,67% | 65,33% | 54,09% | 74,07% | 25,93% | 60,11% | 7,41% | 92,59% | 85,75% | 40,00% | 60,00% | 50,00% | 40,00% | 60,00% | 50,00% | 17,39% | 82,61% | 69,96% | 60,87% | 39,13% | 50,20% |
| pettinarsi | 93,02% | 6,98% | 86,87% | 4,65% | 95,35% | 91,03% | 90,91% | 9,09% | 83,09% | 2,27% | 97,73% | 95,45% | 95,45% | 4,55% | 90,91% | 13,64% | 86,36% | 75,32% | 95,00% | 5,00% | 90,00% | 0,00% | 100,00% | 100,00% |
| piacere | 10,00% | 90,00% | 81,77% | 76,25% | 23,75% | 63,32% | 16,22% | 83,78% | 72,07% | 78,38% | 21,62% | 65,17% | 0,00% | 100,00% | 100,00% | 84,62% | 15,38% | 72,92% | 11,76% | 88,24% | 77,94% | 58,82% | 41,18% | 48,53% |
| piacersi | 4,76% | 95,24% | 90,82% | 82,14% | 17,86% | 70,31% | 9,76% | 90,24% | 81,95% | 80,49% | 19,51% | 67,80% | 0,00% | 100,00% | 100,00% | 86,96% | 13,04% | 76,28% | 0,00% | 100,00% | 100,00% | 80,00% | 20,00% | 66,32% |
| piangere | 27,38% | 72,62% | 59,75% | 36,90% | 63,10% | 52,87% | 25,00% | 75,00% | 61,29% | 43,75% | 56,25% | 49,19% | 20,00% | 80,00% | 66,67% | 40,00% | 60,00% | 50,00% | 37,04% | 62,96% | 51,57% | 25,93% | 74,07% | 60,11% |
| piegare | 96,47% | 3,53% | 93,11% | 4,71% | 95,29% | 90,92% | 95,45% | 4,55% | 91,12% | 2,27% | 97,73% | 95,45% | 95,24% | 4,76% | 90,48% | 14,29% | 85,71% | 74,29% | 100,00% | 0,00% | 100,00% | 0,00% | 100,00% | 100,00% |
| pinzare | 100,00% | 0,00% | 100,00% | 1,32% | 98,68% | 97,37% | 100,00% | 0,00% | 100,00% | 3,23% | 96,77% | 93,55% | 100,00% | 0,00% | 100,00% | 0,00% | 100,00% | 100,00% | 100,00% | 0,00% | 100,00% | 0,00% | 100,00% | 100,00% |
| pizzicarsi | 85,92% | 14,08% | 75,45% | 1,41% | 98,59% | 97,18% | 91,89% | 8,11% | 84,68% | 2,70% | 97,30% | 94,59% | 85,00% | 15,00% | 73,16% | 0,00% | 100,00% | 100,00% | 71,43% | 28,57% | 56,04% | 0,00% | 100,00% | 100,00% |
| posare | 90,41% | 9,59% | 82,42% | 4,11% | 95,89% | 92,01% | 86,21% | 13,79% | 75,37% | 0,00% | 100,00% | 100,00% | 90,48% | 9,52% | 81,90% | 9,52% | 90,48% | 81,90% | 95,65% | 4,35% | 91,30% | 4,35% | 95,65% | 91,30% |
| premere | 93,90% | 6,10% | 88,41% | 0,00% | 100,00% | 100,00% | 100,00% | 0,00% | 100,00% | 0,00% | 100,00% | 100,00% | 87,50% | 12,50% | 77,17% | 0,00% | 100,00% | 100,00% | 90,48% | 9,52% | 81,90% | 0,00% | 100,00% | 100,00% |
| prendere | 94,05% | 5,95% | 88,67% | 2,38% | 97,62% | 95,30% | 91,89% | 8,11% | 84,68% | 5,41% | 94,59% | 89,49% | 96,00% | 4,00% | 92,00% | 0,00% | 100,00% | 100,00% | 95,45% | 4,55% | 90,91% | 0,00% | 100,00% | 100,00% |
| preoccuparsi | 4,76% | 95,24% | 90,82% | 86,90% | 13,10% | 76,97% | 5,13% | 94,87% | 90,01% | 87,18% | 12,82% | 77,06% | 8,00% | 92,00% | 84,67% | 80,00% | 20,00% | 66,67% | 0,00% | 100,00% | 100,00% | 95,00% | 5,00% | 90,00% |
| punire | 54,81% | 45,19% | 49,98% | 40,38% | 59,62% | 51,38% | 66,07% | 33,93% | 54,35% | 33,93% | 66,07% | 54,35% | 46,15% | 53,85% | 48,31% | 46,15% | 53,85% | 48,31% | 36,36% | 63,64% | 51,52% | 50,00% | 50,00% | 47,62% |
| punirsi | 43,18% | 56,82% | 50,37% | 37,50% | 62,50% | 52,59% | 50,00% | 50,00% | 48,72% | 40,00% | 60,00% | 50,77% | 35,71% | 64,29% | 52,38% | 42,86% | 57,14% | 49,21% | 40,00% | 60,00% | 49,47% | 25,00% | 75,00% | 60,53% |
| raccogliere | 95,35% | 4,65% | 91,03% | 0,00% | 100,00% | 100,00% | 97,50% | 2,50% | 95,00% | 0,00% | 100,00% | 100,00% | 88,89% | 11,11% | 79,49% | 0,00% | 100,00% | 100,00% | 100,00% | 0,00% | 100,00% | 0,00% | 100,00% | 100,00% |
| rallegrarsi | 5,00% | 95,00% | 90,38% | 90,00% | 10,00% | 81,77% | 5,71% | 94,29% | 88,91% | 85,71% | 14,29% | 74,79% | 3,70% | 96,30% | 92,59% | 100,00% | 0,00% | 100,00% | 5,56% | 94,44% | 88,89% | 83,33% | 16,67% | 70,59% |
| rastrellare | 98,72% | 1,28% | 97,44% | 2,56% | 97,44% | 94,94% | 100,00% | 0,00% | 100,00% | 2,94% | 97,06% | 94,12% | 100,00% | 0,00% | 100,00% | 0,00% | 100,00% | 100,00% | 95,45% | 4,55% | 90,91% | 4,55% | 95,45% | 90,91% |
| rattristarsi | 0,00% | 100,00% | 100,00% | 84,42% | 15,58% | 73,34% | 0,00% | 100,00% | 100,00% | 90,91% | 9,09% | 82,95% | 0,00% | 100,00% | 100,00% | 79,17% | 20,83% | 65,58% | 0,00% | 100,00% | 100,00% | 80,00% | 20,00% | 66,32% |
| ridere | 31,40% | 68,60% | 56,42% | 24,42% | 75,58% | 62,65% | 30,56% | 69,44% | 56,35% | 27,78% | 72,22% | 58,73% | 29,17% | 70,83% | 56,88% | 25,00% | 75,00% | 60,87% | 34,62% | 65,38% | 52,92% | 19,23% | 80,77% | 67,69% |
| rifiutare | 35,63% | 64,37% | 53,60% | 56,32% | 43,68% | 50,23% | 41,30% | 58,70% | 50,43% | 58,70% | 41,30% | 50,43% | 31,58% | 68,42% | 54,39% | 63,16% | 36,84% | 50,88% | 27,27% | 72,73% | 58,44% | 45,45% | 54,55% | 48,05% |
| rilassarsi | 34,15% | 65,85% | 54,47% | 34,15% | 65,85% | 54,47% | 48,48% | 51,52% | 48,48% | 27,27% | 72,73% | 59,09% | 36,00% | 64,00% | 52,00% | 36,00% | 64,00% | 52,00% | 12,50% | 87,50% | 77,17% | 41,67% | 58,33% | 49,28% |
| rubare | 73,91% | 26,09% | 61,01% | 6,52% | 93,48% | 87,67% | 69,39% | 30,61% | 56,63% | 12,24% | 87,76% | 78,06% | 81,82% | 18,18% | 68,83% | 0,00% | 100,00% | 100,00% | 76,19% | 23,81% | 61,90% | 0,00% | 100,00% | 100,00% |
| ruotare | 93,06% | 6,94% | 86,89% | 4,17% | 95,83% | 91,90% | 86,49% | 13,51% | 75,98% | 5,41% | 94,59% | 89,49% | 100,00% | 0,00% | 100,00% | 5,00% | 95,00% | 90,00% | 100,00% | 0,00% | 100,00% | 0,00% | 100,00% | 100,00% |
| sanguinare | 83,33% | 16,67% | 71,86% | 11,54% | 88,46% | 79,32% | 78,57% | 21,43% | 65,51% | 14,29% | 85,71% | 74,91% | 90,91% | 9,09% | 82,68% | 4,55% | 95,45% | 90,91% | 85,71% | 14,29% | 73,63% | 14,29% | 85,71% | 73,63% |
| sbagliare | 38,27% | 61,73% | 52,16% | 53,09% | 46,91% | 49,57% | 50,00% | 50,00% | 48,57% | 50,00% | 50,00% | 48,57% | 37,50% | 62,50% | 51,09% | 50,00% | 50,00% | 47,83% | 19,05% | 80,95% | 67,62% | 61,90% | 38,10% | 50,48% |
| sbattere | 87,21% | 12,79% | 77,43% | 3,49% | 96,51% | 93,19% | 94,74% | 5,26% | 89,76% | 5,26% | 94,74% | 89,76% | 80,00% | 20,00% | 66,67% | 0,00% | 100,00% | 100,00% | 82,61% | 17,39% | 69,96% | 4,35% | 95,65% | 91,30% |
| scarabocchiare | 94,05% | 5,95% | 88,67% | 0,00% | 100,00% | 100,00% | 100,00% | 0,00% | 100,00% | 0,00% | 100,00% | 100,00% | 87,50% | 12,50% | 77,17% | 0,00% | 100,00% | 100,00% | 90,00% | 10,00% | 81,05% | 0,00% | 100,00% | 100,00% |
| schiacciare | 88,76% | 11,24% | 79,83% | 3,37% | 96,63% | 93,41% | 97,50% | 2,50% | 95,00% | 0,00% | 100,00% | 100,00% | 89,29% | 10,71% | 80,16% | 3,57% | 96,43% | 92,86% | 71,43% | 28,57% | 57,14% | 9,52% | 90,48% | 81,90% |
| sconfiggere | 52,00% | 48,00% | 49,58% | 24,00% | 76,00% | 63,15% | 67,35% | 32,65% | 55,10% | 18,37% | 81,63% | 69,39% | 48,15% | 51,85% | 48,15% | 11,11% | 88,89% | 79,49% | 25,00% | 75,00% | 60,87% | 50,00% | 50,00% | 47,83% |
| scoraggiarsi | 6,85% | 93,15% | 87,06% | 87,67% | 12,33% | 78,08% | 11,11% | 88,89% | 79,49% | 81,48% | 18,52% | 68,66% | 4,17% | 95,83% | 91,67% | 87,50% | 12,50% | 77,17% | 4,55% | 95,45% | 90,91% | 95,45% | 4,55% | 90,91% |
| scrivere | 90,29% | 9,71% | 82,30% | 1,94% | 98,06% | 96,15% | 90,74% | 9,26% | 82,88% | 0,00% | 100,00% | 100,00% | 84,00% | 16,00% | 72,00% | 8,00% | 92,00% | 84,67% | 95,83% | 4,17% | 91,67% | 0,00% | 100,00% | 100,00% |
| scuotere | 90,36% | 9,64% | 82,37% | 0,00% | 100,00% | 100,00% | 97,30% | 2,70% | 94,59% | 0,00% | 100,00% | 100,00% | 88,89% | 11,11% | 79,49% | 0,00% | 100,00% | 100,00% | 78,95% | 21,05% | 64,91% | 0,00% | 100,00% | 100,00% |
| scusare | 15,66% | 84,34% | 73,26% | 72,29% | 27,71% | 59,45% | 27,78% | 72,22% | 58,73% | 66,67% | 33,33% | 54,29% | 7,69% | 92,31% | 85,23% | 80,77% | 19,23% | 67,69% | 4,76% | 95,24% | 90,48% | 71,43% | 28,57% | 57,14% |
| scusarsi | 24,39% | 75,61% | 62,66% | 63,41% | 36,59% | 53,03% | 33,33% | 66,67% | 54,39% | 53,85% | 46,15% | 48,99% | 16,67% | 83,33% | 71,01% | 66,67% | 33,33% | 53,62% | 15,79% | 84,21% | 71,93% | 78,95% | 21,05% | 64,91% |
| segare | 95,06% | 4,94% | 90,49% | 2,47% | 97,53% | 95,12% | 94,12% | 5,88% | 88,59% | 5,88% | 94,12% | 88,59% | 96,43% | 3,57% | 92,86% | 0,00% | 100,00% | 100,00% | 94,74% | 5,26% | 89,47% | 0,00% | 100,00% | 100,00% |
| sfidare | 44,58% | 55,42% | 49,99% | 31,33% | 68,67% | 56,45% | 56,52% | 43,48% | 49,76% | 17,39% | 82,61% | 70,63% | 31,58% | 68,42% | 54,39% | 47,37% | 52,63% | 47,37% | 27,78% | 72,22% | 57,52% | 50,00% | 50,00% | 47,06% |
| sganciare | 92,00% | 8,00% | 85,08% | 2,67% | 97,33% | 94,74% | 93,55% | 6,45% | 87,53% | 0,00% | 100,00% | 100,00% | 91,30% | 8,70% | 83,40% | 0,00% | 100,00% | 100,00% | 90,48% | 9,52% | 81,90% | 9,52% | 90,48% | 81,90% |
| slegarsi | 92,50% | 7,50% | 85,95% | 1,25% | 98,75% | 97,50% | 95,12% | 4,88% | 90,49% | 0,00% | 100,00% | 100,00% | 87,50% | 12,50% | 77,17% | 4,17% | 95,83% | 91,67% | 93,33% | 6,67% | 86,67% | 0,00% | 100,00% | 100,00% |
| soffrire | 20,24% | 79,76% | 67,33% | 51,19% | 48,81% | 49,43% | 29,27% | 70,73% | 57,56% | 48,78% | 51,22% | 48,78% | 9,52% | 90,48% | 81,90% | 66,67% | 33,33% | 53,33% | 13,64% | 86,36% | 75,32% | 40,91% | 59,09% | 49,35% |
| sognare | 17,65% | 82,35% | 70,59% | 83,53% | 16,47% | 72,16% | 12,82% | 87,18% | 77,06% | 87,18% | 12,82% | 77,06% | 24,00% | 76,00% | 62,00% | 76,00% | 24,00% | 62,00% | 19,05% | 80,95% | 67,62% | 85,71% | 14,29% | 74,29% |
| sollevare | 85,54% | 14,46% | 74,96% | 2,41% | 97,59% | 95,24% | 86,36% | 13,64% | 75,90% | 2,27% | 97,73% | 95,45% | 85,00% | 15,00% | 73,16% | 5,00% | 95,00% | 90,00% | 84,21% | 15,79% | 71,93% | 0,00% | 100,00% | 100,00% |
| sospettare | 13,33% | 86,67% | 76,67% | 86,67% | 13,33% | 76,67% | 16,67% | 83,33% | 71,70% | 79,63% | 20,37% | 66,95% | 11,11% | 88,89% | 79,49% | 92,59% | 7,41% | 85,75% | 8,33% | 91,67% | 84,06% | 95,83% | 4,17% | 91,67% |
| spalmare | 98,80% | 1,20% | 97,59% | 1,20% | 98,80% | 97,59% | 97,37% | 2,63% | 94,74% | 2,63% | 97,37% | 94,74% | 100,00% | 0,00% | 100,00% | 0,00% | 100,00% | 100,00% | 100,00% | 0,00% | 100,00% | 0,00% | 100,00% | 100,00% |
| spaventare | 46,25% | 53,75% | 49,65% | 33,75% | 66,25% | 54,72% | 66,67% | 33,33% | 54,17% | 21,21% | 78,79% | 65,53% | 34,78% | 65,22% | 52,57% | 34,78% | 65,22% | 52,57% | 29,17% | 70,83% | 56,88% | 50,00% | 50,00% | 47,83% |
| spaventarsi | 18,75% | 81,25% | 69,15% | 60,00% | 40,00% | 51,39% | 31,58% | 68,42% | 55,62% | 52,63% | 47,37% | 48,79% | 4,55% | 95,45% | 90,91% | 77,27% | 22,73% | 63,20% | 10,00% | 90,00% | 81,05% | 55,00% | 45,00% | 47,89% |
| spazzolarsi | 92,59% | 7,41% | 86,11% | 3,70% | 96,30% | 92,78% | 94,59% | 5,41% | 89,49% | 5,41% | 94,59% | 89,49% | 91,67% | 8,33% | 84,06% | 0,00% | 100,00% | 100,00% | 90,00% | 10,00% | 81,05% | 5,00% | 95,00% | 90,00% |
| sperare | 5,88% | 94,12% | 88,80% | 95,29% | 4,71% | 90,92% | 7,89% | 92,11% | 85,06% | 97,37% | 2,63% | 94,74% | 4,17% | 95,83% | 91,67% | 91,67% | 8,33% | 84,06% | 4,35% | 95,65% | 91,30% | 95,65% | 4,35% | 91,30% |
| spezzare | 87,06% | 12,94% | 77,20% | 2,35% | 97,65% | 95,35% | 94,87% | 5,13% | 90,01% | 2,56% | 97,44% | 94,87% | 81,48% | 18,52% | 68,66% | 0,00% | 100,00% | 100,00% | 78,95% | 21,05% | 64,91% | 5,26% | 94,74% | 89,47% |
| spingere | 95,12% | 4,88% | 90,61% | 2,44% | 97,56% | 95,18% | 96,88% | 3,13% | 93,75% | 3,13% | 96,88% | 93,75% | 91,67% | 8,33% | 84,06% | 4,17% | 95,83% | 91,67% | 96,15% | 3,85% | 92,31% | 0,00% | 100,00% | 100,00% |
| spremere | 89,41% | 10,59% | 80,84% | 9,41% | 90,59% | 82,75% | 88,57% | 11,43% | 79,16% | 5,71% | 94,29% | 88,91% | 88,00% | 12,00% | 78,00% | 16,00% | 84,00% | 72,00% | 92,00% | 8,00% | 84,67% | 8,00% | 92,00% | 84,67% |
| spruzzarsi | 93,90% | 6,10% | 88,41% | 3,66% | 96,34% | 92,86% | 94,74% | 5,26% | 89,76% | 5,26% | 94,74% | 89,76% | 90,91% | 9,09% | 82,68% | 4,55% | 95,45% | 90,91% | 95,45% | 4,55% | 90,91% | 0,00% | 100,00% | 100,00% |
| staccare | 88,46% | 11,54% | 79,32% | 2,56% | 97,44% | 94,94% | 89,47% | 10,53% | 80,65% | 2,63% | 97,37% | 94,74% | 82,61% | 17,39% | 69,96% | 4,35% | 95,65% | 91,30% | 94,12% | 5,88% | 88,24% | 0,00% | 100,00% | 100,00% |
| stringersi | 73,33% | 26,67% | 60,51% | 0,95% | 99,05% | 98,10% | 78,57% | 21,43% | 65,71% | 0,00% | 100,00% | 100,00% | 73,08% | 26,92% | 59,08% | 0,00% | 100,00% | 100,00% | 60,87% | 39,13% | 50,20% | 4,35% | 95,65% | 91,30% |
| strizzare | 93,81% | 6,19% | 88,27% | 0,00% | 100,00% | 100,00% | 91,84% | 8,16% | 84,69% | 0,00% | 100,00% | 100,00% | 92,59% | 7,41% | 85,75% | 0,00% | 100,00% | 100,00% | 100,00% | 0,00% | 100,00% | 0,00% | 100,00% | 100,00% |
| strofinare | 96,25% | 3,75% | 92,69% | 2,50% | 97,50% | 95,06% | 97,14% | 2,86% | 94,29% | 0,00% | 100,00% | 100,00% | 92,00% | 8,00% | 84,67% | 4,00% | 96,00% | 92,00% | 100,00% | 0,00% | 100,00% | 5,00% | 95,00% | 90,00% |
| suicidarsi | 70,59% | 29,41% | 57,98% | 7,06% | 92,94% | 86,72% | 91,89% | 8,11% | 84,68% | 8,11% | 91,89% | 84,68% | 60,71% | 39,29% | 50,53% | 3,57% | 96,43% | 92,86% | 45,00% | 55,00% | 47,89% | 10,00% | 90,00% | 81,05% |
| svitare | 95,06% | 4,94% | 90,49% | 2,47% | 97,53% | 95,12% | 90,63% | 9,38% | 82,46% | 0,00% | 100,00% | 100,00% | 96,00% | 4,00% | 92,00% | 8,00% | 92,00% | 84,67% | 100,00% | 0,00% | 100,00% | 0,00% | 100,00% | 100,00% |
| temere | 3,88% | 96,12% | 92,46% | 85,44% | 14,56% | 74,87% | 5,56% | 94,44% | 89,31% | 83,33% | 16,67% | 71,70% | 0,00% | 100,00% | 100,00% | 80,77% | 19,23% | 67,69% | 4,35% | 95,65% | 91,30% | 95,65% | 4,35% | 91,30% |
| terrorizzare | 37,66% | 62,34% | 52,43% | 38,96% | 61,04% | 51,81% | 48,84% | 51,16% | 48,84% | 32,56% | 67,44% | 55,04% | 20,00% | 80,00% | 66,32% | 50,00% | 50,00% | 47,37% | 28,57% | 71,43% | 56,04% | 42,86% | 57,14% | 47,25% |
| terrorizzarsi | 3,77% | 96,23% | 92,67% | 85,85% | 14,15% | 75,47% | 5,36% | 94,64% | 89,68% | 87,50% | 12,50% | 77,73% | 3,85% | 96,15% | 92,31% | 84,62% | 15,38% | 72,92% | 0,00% | 100,00% | 100,00% | 83,33% | 16,67% | 71,01% |
| tirare | 88,24% | 11,76% | 78,99% | 0,00% | 100,00% | 100,00% | 94,74% | 5,26% | 89,76% | 0,00% | 100,00% | 100,00% | 87,50% | 12,50% | 77,17% | 0,00% | 100,00% | 100,00% | 78,26% | 21,74% | 64,43% | 0,00% | 100,00% | 100,00% |
| toccare | 90,59% | 9,41% | 82,75% | 0,00% | 100,00% | 100,00% | 95,00% | 5,00% | 90,26% | 0,00% | 100,00% | 100,00% | 88,46% | 11,54% | 78,77% | 0,00% | 100,00% | 100,00% | 84,21% | 15,79% | 71,93% | 0,00% | 100,00% | 100,00% |
| torturare | 62,65% | 37,35% | 52,63% | 14,46% | 85,54% | 74,96% | 71,11% | 28,89% | 57,98% | 11,11% | 88,89% | 79,80% | 45,00% | 55,00% | 47,89% | 25,00% | 75,00% | 60,53% | 61,11% | 38,89% | 49,67% | 11,11% | 88,89% | 79,08% |
| tradire | 19,61% | 80,39% | 68,16% | 67,65% | 32,35% | 55,79% | 23,08% | 76,92% | 63,80% | 75,00% | 25,00% | 61,76% | 23,08% | 76,92% | 63,08% | 61,54% | 38,46% | 50,77% | 8,33% | 91,67% | 84,06% | 58,33% | 41,67% | 49,28% |
| vendicarsi | 23,17% | 76,83% | 63,96% | 48,78% | 51,22% | 49,41% | 23,68% | 76,32% | 62,87% | 55,26% | 44,74% | 49,22% | 37,50% | 62,50% | 51,09% | 29,17% | 70,83% | 56,88% | 5,00% | 95,00% | 90,00% | 60,00% | 40,00% | 49,47% |
| vergognarsi | 1,20% | 98,80% | 97,59% | 87,95% | 12,05% | 78,55% | 2,44% | 97,56% | 95,12% | 92,68% | 7,32% | 86,10% | 0,00% | 100,00% | 100,00% | 86,96% | 13,04% | 76,28% | 0,00% | 100,00% | 100,00% | 78,95% | 21,05% | 64,91% |
| versare | 98,82% | 1,18% | 97,65% | 1,18% | 98,82% | 97,65% | 100,00% | 0,00% | 100,00% | 0,00% | 100,00% | 100,00% | 100,00% | 0,00% | 100,00% | 0,00% | 100,00% | 100,00% | 95,45% | 4,55% | 90,91% | 4,55% | 95,45% | 90,91% |
| vestirsi | 94,87% | 5,13% | 90,14% | 2,56% | 97,44% | 94,94% | 96,97% | 3,03% | 93,94% | 6,06% | 93,94% | 88,26% | 91,30% | 8,70% | 83,40% | 0,00% | 100,00% | 100,00% | 95,45% | 4,55% | 90,91% | 0,00% | 100,00% | 100,00% |

**Supplementary Table 1** - showing, for each verb, the percentages of the emotion and motor relatedness judgments. The percentages are reported for each age range (8-11 ALL, 12-15 ALL, 16-19 ALL), for males and females within each age range(8-11 femal\es, 8-11 males; 12-15 females, 12-15 males; 16-19 females, 16-19 males), for males and females within the whole sample (females ALL, males ALL) and for the whole sample (ALL).
