# Supplementary material for: Age-Dependent Changes of Thinking about Verbs
Source: Front Behav Neurosci. 2017 Mar 14;11:40. doi: 10.3389/fnbeh.2017.00040 (PMC5348498; doi:10.3389/fnbeh.2017.00040)
Supplement: Supplementary file 2 [file Table2.DOCX]

**Supplementary Table 2 – familiarity**

| **Familliarity** | | **age range -> 8-19 (0VERALL SAMPLE)** | | | **age range -> 8-11** | | | **age range ->12-15** | | | **age range ->16-19** | | |
| --- | --- | --- | --- | --- | --- | --- | --- | --- | --- | --- | --- | --- | --- |
|  | R -NR | ALL | F | M | ALL | F | M | ALL | F | M | ALL | F | M |
|  |  | % | % | % | % | % | % | % | % | % | % | % | % |
| abbottonarsi | NR | 97,53% | 97,50% | 97,56% | 94,59% | 94,44% | 94,74% | 100,00% | 100,00% | 100,00% | 100,00% | 100,00% | 100,00% |
| afferrare | NR | 100,00% | 100,00% | 100,00% | 100,00% | 100,00% | 100,00% | 100,00% | 100,00% | 100,00% | 100,00% | 100,00% | 100,00% |
| affettare | NR | 85,39% | 86,96% | 83,72% | 93,33% | 95,83% | 90,48% | 90,91% | 90,91% | 90,91% | 63,64% | 63,64% | 63,64% |
| aggrapparsi | R | 98,84% | 100,00% | 97,37% | 97,50% | 100,00% | 94,12% | 100,00% | 100,00% | 100,00% | 100,00% | 100,00% | 100,00% |
| allacciarsi | R | 98,80% | 100,00% | 97,22% | 100,00% | 100,00% | 100,00% | 100,00% | 100,00% | 100,00% | 95,24% | 100,00% | 88,89% |
| amare | NR | 100,00% | 100,00% | 100,00% | 100,00% | 100,00% | 100,00% | 100,00% | 100,00% | 100,00% | 100,00% | 100,00% | 100,00% |
| amarsi | R | 97,62% | 100,00% | 95,35% | 97,67% | 100,00% | 95,65% | 95,45% | 100,00% | 90,00% | 100,00% | 100,00% | 100,00% |
| ammalarsi | R | 100,00% | 100,00% | 100,00% | 100,00% | 100,00% | 100,00% | 100,00% | 100,00% | 100,00% | 100,00% | 100,00% | 100,00% |
| annodare | NR | 97,14% | 96,23% | 98,08% | 94,44% | 92,86% | 96,15% | 100,00% | 100,00% | 100,00% | 100,00% | 100,00% | 100,00% |
| annoiarsi | R | 98,81% | 97,44% | 100,00% | 100,00% | 100,00% | 100,00% | 100,00% | 100,00% | 100,00% | 95,00% | 90,00% | 100,00% |
| apparecchiare | NR | 98,78% | 97,67% | 100,00% | 100,00% | 100,00% | 100,00% | 100,00% | 100,00% | 100,00% | 96,15% | 93,33% | 100,00% |
| appendere | NR | 98,81% | 100,00% | 97,62% | 97,50% | 100,00% | 95,00% | 100,00% | 100,00% | 100,00% | 100,00% | 100,00% | 100,00% |
| applaudire | NR | 98,78% | 100,00% | 97,73% | 97,44% | 100,00% | 95,45% | 100,00% | 100,00% | 100,00% | 100,00% | 100,00% | 100,00% |
| arrabbiarsi | R | 100,00% | 100,00% | 100,00% | 100,00% | 100,00% | 100,00% | 100,00% | 100,00% | 100,00% | 100,00% | 100,00% | 100,00% |
| avvitare | NR | 94,87% | 94,29% | 95,35% | 97,06% | 100,00% | 95,24% | 100,00% | 100,00% | 100,00% | 85,00% | 80,00% | 90,00% |
| avvolgersi | R | 90,80% | 91,49% | 90,00% | 86,11% | 83,33% | 88,89% | 100,00% | 100,00% | 100,00% | 88,89% | 93,75% | 81,82% |
| bussare | NR | 100,00% | 100,00% | 100,00% | 100,00% | 100,00% | 100,00% | 100,00% | 100,00% | 100,00% | 100,00% | 100,00% | 100,00% |
| calmare | NR | 98,82% | 100,00% | 97,87% | 97,44% | 100,00% | 95,83% | 100,00% | 100,00% | 100,00% | 100,00% | 100,00% | 100,00% |
| calmarsi | R | 100,00% | 100,00% | 100,00% | 100,00% | 100,00% | 100,00% | 100,00% | 100,00% | 100,00% | 100,00% | 100,00% | 100,00% |
| cancellare | NR | 100,00% | 100,00% | 100,00% | 100,00% | 100,00% | 100,00% | 100,00% | 100,00% | 100,00% | 100,00% | 100,00% | 100,00% |
| confessare | NR | 96,59% | 100,00% | 92,86% | 94,59% | 100,00% | 89,47% | 100,00% | 100,00% | 100,00% | 96,15% | 100,00% | 90,91% |
| confondersi | R | 96,51% | 97,56% | 95,56% | 95,12% | 100,00% | 90,91% | 100,00% | 100,00% | 100,00% | 95,00% | 90,00% | 100,00% |
| controllare | NR | 100,00% | 100,00% | 100,00% | 100,00% | 100,00% | 100,00% | 100,00% | 100,00% | 100,00% | 100,00% | 100,00% | 100,00% |
| controllarsi | R | 96,30% | 93,02% | 100,00% | 97,44% | 95,65% | 100,00% | 100,00% | 100,00% | 100,00% | 90,48% | 80,00% | 100,00% |
| credere | NR | 100,00% | 100,00% | 100,00% | 100,00% | 100,00% | 100,00% | 100,00% | 100,00% | 100,00% | 100,00% | 100,00% | 100,00% |
| decidere | NR | 100,00% | 100,00% | 100,00% | 100,00% | 100,00% | 100,00% | 100,00% | 100,00% | 100,00% | 100,00% | 100,00% | 100,00% |
| deludere | NR | 98,80% | 100,00% | 97,37% | 100,00% | 100,00% | 100,00% | 100,00% | 100,00% | 100,00% | 95,45% | 100,00% | 90,00% |
| deprimersi | R | 77,38% | 76,09% | 78,95% | 65,00% | 58,33% | 75,00% | 86,96% | 91,67% | 81,82% | 90,48% | 100,00% | 81,82% |
| desiderare | NR | 100,00% | 100,00% | 100,00% | 100,00% | 100,00% | 100,00% | 100,00% | 100,00% | 100,00% | 100,00% | 100,00% | 100,00% |
| dimenticare | NR | 98,84% | 97,83% | 100,00% | 100,00% | 100,00% | 100,00% | 100,00% | 100,00% | 100,00% | 96,15% | 93,75% | 100,00% |
| dipingere | NR | 100,00% | 100,00% | 100,00% | 100,00% | 100,00% | 100,00% | 100,00% | 100,00% | 100,00% | 100,00% | 100,00% | 100,00% |
| disegnare | NR | 100,00% | 100,00% | 100,00% | 100,00% | 100,00% | 100,00% | 100,00% | 100,00% | 100,00% | 100,00% | 100,00% | 100,00% |
| dispiacersi | R | 96,39% | 97,83% | 94,59% | 95,56% | 96,00% | 95,00% | 100,00% | 100,00% | 100,00% | 95,24% | 100,00% | 90,00% |
| fallire | NR | 98,81% | 100,00% | 97,50% | 97,14% | 100,00% | 94,74% | 100,00% | 100,00% | 100,00% | 100,00% | 100,00% | 100,00% |
| fingere | NR | 97,56% | 100,00% | 95,74% | 94,44% | 100,00% | 91,67% | 100,00% | 100,00% | 100,00% | 100,00% | 100,00% | 100,00% |
| fotografarsi | R | 90,91% | 93,48% | 88,10% | 100,00% | 100,00% | 100,00% | 95,24% | 100,00% | 90,91% | 65,00% | 72,73% | 55,56% |
| gettare | NR | 97,65% | 97,37% | 97,87% | 94,87% | 93,33% | 95,83% | 100,00% | 100,00% | 100,00% | 100,00% | 100,00% | 100,00% |
| graffiarsi | R | 100,00% | 100,00% | 100,00% | 100,00% | 100,00% | 100,00% | 100,00% | 100,00% | 100,00% | 100,00% | 100,00% | 100,00% |
| grattarsi | R | 98,72% | 97,67% | 100,00% | 96,88% | 94,12% | 100,00% | 100,00% | 100,00% | 100,00% | 100,00% | 100,00% | 100,00% |
| grattugiare | NR | 93,26% | 100,00% | 86,67% | 97,83% | 100,00% | 95,45% | 95,24% | 100,00% | 90,91% | 81,82% | 100,00% | 66,67% |
| guarire | NR | 98,78% | 100,00% | 97,37% | 96,97% | 100,00% | 94,12% | 100,00% | 100,00% | 100,00% | 100,00% | 100,00% | 100,00% |
| impaurirsi | R | 98,02% | 98,00% | 98,04% | 98,11% | 96,43% | 100,00% | 100,00% | 100,00% | 100,00% | 95,45% | 100,00% | 92,31% |
| impazzire | NR | 94,19% | 95,83% | 92,11% | 94,74% | 95,65% | 93,33% | 96,00% | 100,00% | 91,67% | 91,30% | 91,67% | 90,91% |
| impressionarsi | R | 92,94% | 92,86% | 93,02% | 90,00% | 95,00% | 85,00% | 100,00% | 100,00% | 100,00% | 90,00% | 80,00% | 100,00% |
| inchiodare | NR | 89,29% | 92,86% | 85,71% | 95,35% | 100,00% | 90,48% | 95,00% | 100,00% | 90,00% | 71,43% | 70,00% | 72,73% |
| incollarsi | R | 85,54% | 88,10% | 82,93% | 95,24% | 95,65% | 94,74% | 95,00% | 100,00% | 90,91% | 57,14% | 60,00% | 54,55% |
| indicarsi | R | 96,00% | 93,88% | 98,04% | 93,75% | 91,67% | 95,83% | 96,43% | 92,86% | 100,00% | 100,00% | 100,00% | 100,00% |
| infilare | NR | 99,03% | 98,11% | 100,00% | 98,21% | 96,55% | 100,00% | 100,00% | 100,00% | 100,00% | 100,00% | 100,00% | 100,00% |
| ingelosirsi | R | 96,20% | 100,00% | 91,43% | 97,14% | 100,00% | 92,86% | 95,65% | 100,00% | 90,91% | 95,24% | 100,00% | 90,00% |
| insultare | NR | 98,78% | 97,67% | 100,00% | 100,00% | 100,00% | 100,00% | 100,00% | 100,00% | 100,00% | 96,30% | 93,75% | 100,00% |
| intrecciarsi | R | 81,61% | 82,61% | 80,49% | 80,56% | 76,47% | 84,21% | 84,00% | 84,62% | 83,33% | 80,77% | 87,50% | 70,00% |
| intristirsi | R | 94,05% | 95,56% | 92,31% | 85,71% | 88,89% | 82,35% | 100,00% | 100,00% | 100,00% | 100,00% | 100,00% | 100,00% |
| invidiare | NR | 94,12% | 95,65% | 92,31% | 91,43% | 94,44% | 88,24% | 100,00% | 100,00% | 100,00% | 92,59% | 93,75% | 90,91% |
| irritarsi | NR | 90,24% | 93,62% | 85,71% | 82,86% | 86,36% | 76,92% | 96,00% | 100,00% | 91,67% | 95,45% | 100,00% | 90,00% |
| lanciare | NR | 100,00% | 100,00% | 100,00% | 100,00% | 100,00% | 100,00% | 100,00% | 100,00% | 100,00% | 100,00% | 100,00% | 100,00% |
| lavarsi | R | 100,00% | 100,00% | 100,00% | 100,00% | 100,00% | 100,00% | 100,00% | 100,00% | 100,00% | 100,00% | 100,00% | 100,00% |
| legare | NR | 98,85% | 97,87% | 100,00% | 100,00% | 100,00% | 100,00% | 95,83% | 92,31% | 100,00% | 100,00% | 100,00% | 100,00% |
| maledire | NR | 91,01% | 92,50% | 89,80% | 80,49% | 81,25% | 80,00% | 100,00% | 100,00% | 100,00% | 100,00% | 100,00% | 100,00% |
| martellare | NR | 92,94% | 95,65% | 89,74% | 91,89% | 95,24% | 87,50% | 96,00% | 100,00% | 91,67% | 91,30% | 91,67% | 90,91% |
| mentire | NR | 97,53% | 97,44% | 97,62% | 95,00% | 94,74% | 95,24% | 100,00% | 100,00% | 100,00% | 100,00% | 100,00% | 100,00% |
| meritare | NR | 95,29% | 93,18% | 97,56% | 95,24% | 91,30% | 100,00% | 100,00% | 100,00% | 100,00% | 90,91% | 90,91% | 90,91% |
| mescolare | NR | 96,43% | 100,00% | 93,02% | 95,12% | 100,00% | 90,48% | 96,00% | 100,00% | 92,31% | 100,00% | 100,00% | 100,00% |
| morire | NR | 98,88% | 100,00% | 97,67% | 100,00% | 100,00% | 100,00% | 95,24% | 100,00% | 90,91% | 100,00% | 100,00% | 100,00% |
| obbedire | NR | 100,00% | 100,00% | 100,00% | 100,00% | 100,00% | 100,00% | 100,00% | 100,00% | 100,00% | 100,00% | 100,00% | 100,00% |
| odiare | NR | 98,84% | 100,00% | 97,92% | 97,44% | 100,00% | 95,83% | 100,00% | 100,00% | 100,00% | 100,00% | 100,00% | 100,00% |
| odiarsi | R | 92,86% | 95,35% | 90,24% | 96,88% | 92,86% | 100,00% | 92,00% | 100,00% | 83,33% | 88,89% | 93,75% | 81,82% |
| peccare | NR | 84,62% | 81,13% | 88,24% | 75,93% | 71,43% | 80,77% | 92,31% | 92,86% | 91,67% | 95,83% | 90,91% | 100,00% |
| penare | NR | 62,86% | 62,75% | 62,96% | 57,41% | 55,56% | 59,26% | 66,67% | 61,54% | 71,43% | 70,83% | 81,82% | 61,54% |
| pennellarsi | R | 85,98% | 85,19% | 86,79% | 85,96% | 83,33% | 88,89% | 77,78% | 78,57% | 76,92% | 95,65% | 100,00% | 92,31% |
| perdere | NR | 100,00% | 100,00% | 100,00% | 100,00% | 100,00% | 100,00% | 100,00% | 100,00% | 100,00% | 100,00% | 100,00% | 100,00% |
| perdonare | NR | 97,56% | 97,62% | 97,50% | 97,44% | 100,00% | 94,44% | 100,00% | 100,00% | 100,00% | 95,24% | 90,00% | 100,00% |
| perdonarsi | R | 92,77% | 95,00% | 90,70% | 92,50% | 100,00% | 85,71% | 100,00% | 100,00% | 100,00% | 84,21% | 77,78% | 90,00% |
| perseguitare | NR | 88,24% | 89,13% | 87,18% | 77,14% | 76,47% | 77,78% | 100,00% | 100,00% | 100,00% | 92,00% | 93,75% | 88,89% |
| pettinarsi | R | 100,00% | 100,00% | 100,00% | 100,00% | 100,00% | 100,00% | 100,00% | 100,00% | 100,00% | 100,00% | 100,00% | 100,00% |
| piacere | NR | 96,39% | 97,22% | 95,74% | 92,50% | 93,33% | 92,00% | 100,00% | 100,00% | 100,00% | 100,00% | 100,00% | 100,00% |
| piacersi | R | 100,00% | 100,00% | 100,00% | 100,00% | 100,00% | 100,00% | 100,00% | 100,00% | 100,00% | 100,00% | 100,00% | 100,00% |
| piangere | NR | 100,00% | 100,00% | 100,00% | 100,00% | 100,00% | 100,00% | 100,00% | 100,00% | 100,00% | 100,00% | 100,00% | 100,00% |
| piegare | NR | 100,00% | 100,00% | 100,00% | 100,00% | 100,00% | 100,00% | 100,00% | 100,00% | 100,00% | 100,00% | 100,00% | 100,00% |
| pinzare | NR | 89,41% | 83,78% | 93,75% | 83,78% | 69,23% | 91,67% | 92,86% | 92,86% | 92,86% | 95,00% | 90,00% | 100,00% |
| pizzicarsi | R | 84,52% | 88,37% | 80,49% | 90,24% | 95,45% | 84,21% | 95,24% | 100,00% | 90,91% | 63,64% | 63,64% | 63,64% |
| posare | NR | 89,02% | 90,70% | 87,18% | 93,55% | 92,86% | 94,12% | 87,50% | 92,31% | 81,82% | 85,19% | 87,50% | 81,82% |
| premere | NR | 100,00% | 100,00% | 100,00% | 100,00% | 100,00% | 100,00% | 100,00% | 100,00% | 100,00% | 100,00% | 100,00% | 100,00% |
| prendere | NR | 100,00% | 100,00% | 100,00% | 100,00% | 100,00% | 100,00% | 100,00% | 100,00% | 100,00% | 100,00% | 100,00% | 100,00% |
| preoccuparsi | R | 100,00% | 100,00% | 100,00% | 100,00% | 100,00% | 100,00% | 100,00% | 100,00% | 100,00% | 100,00% | 100,00% | 100,00% |
| punire | NR | 100,00% | 100,00% | 100,00% | 100,00% | 100,00% | 100,00% | 100,00% | 100,00% | 100,00% | 100,00% | 100,00% | 100,00% |
| punirsi | R | 98,88% | 97,50% | 100,00% | 97,56% | 93,75% | 100,00% | 100,00% | 100,00% | 100,00% | 100,00% | 100,00% | 100,00% |
| raccogliere | NR | 100,00% | 100,00% | 100,00% | 100,00% | 100,00% | 100,00% | 100,00% | 100,00% | 100,00% | 100,00% | 100,00% | 100,00% |
| rallegrarsi | R | 95,24% | 97,37% | 93,48% | 89,74% | 93,33% | 87,50% | 100,00% | 100,00% | 100,00% | 100,00% | 100,00% | 100,00% |
| rastrellare | NR | 95,12% | 95,45% | 94,74% | 97,14% | 95,00% | 100,00% | 95,65% | 100,00% | 91,67% | 91,67% | 92,31% | 90,91% |
| rattristarsi | R | 92,77% | 95,24% | 90,24% | 84,62% | 90,00% | 78,95% | 100,00% | 100,00% | 100,00% | 100,00% | 100,00% | 100,00% |
| ridere | NR | 100,00% | 100,00% | 100,00% | 100,00% | 100,00% | 100,00% | 100,00% | 100,00% | 100,00% | 100,00% | 100,00% | 100,00% |
| rifiutare | NR | 100,00% | 100,00% | 100,00% | 100,00% | 100,00% | 100,00% | 100,00% | 100,00% | 100,00% | 100,00% | 100,00% | 100,00% |
| rilassarsi | R | 98,80% | 100,00% | 97,50% | 100,00% | 100,00% | 100,00% | 100,00% | 100,00% | 100,00% | 96,00% | 100,00% | 90,00% |
| rubare | NR | 100,00% | 100,00% | 100,00% | 100,00% | 100,00% | 100,00% | 100,00% | 100,00% | 100,00% | 100,00% | 100,00% | 100,00% |
| ruotare | NR | 85,71% | 89,13% | 81,58% | 90,24% | 92,00% | 87,50% | 95,24% | 100,00% | 90,00% | 68,18% | 70,00% | 66,67% |
| sanguinare | NR | 98,73% | 97,56% | 100,00% | 97,67% | 95,83% | 100,00% | 100,00% | 100,00% | 100,00% | 100,00% | 100,00% | 100,00% |
| sbagliare | NR | 100,00% | 100,00% | 100,00% | 100,00% | 100,00% | 100,00% | 100,00% | 100,00% | 100,00% | 100,00% | 100,00% | 100,00% |
| sbattere | NR | 100,00% | 100,00% | 100,00% | 100,00% | 100,00% | 100,00% | 100,00% | 100,00% | 100,00% | 100,00% | 100,00% | 100,00% |
| scarabocchiare | NR | 100,00% | 100,00% | 100,00% | 100,00% | 100,00% | 100,00% | 100,00% | 100,00% | 100,00% | 100,00% | 100,00% | 100,00% |
| schiacciare | NR | 100,00% | 100,00% | 100,00% | 100,00% | 100,00% | 100,00% | 100,00% | 100,00% | 100,00% | 100,00% | 100,00% | 100,00% |
| sconfiggere | NR | 99,01% | 98,08% | 100,00% | 98,00% | 96,30% | 100,00% | 100,00% | 100,00% | 100,00% | 100,00% | 100,00% | 100,00% |
| scoraggiarsi | R | 85,88% | 89,13% | 82,05% | 75,00% | 77,78% | 72,22% | 100,00% | 100,00% | 100,00% | 88,00% | 93,33% | 80,00% |
| scrivere | NR | 100,00% | 100,00% | 100,00% | 100,00% | 100,00% | 100,00% | 100,00% | 100,00% | 100,00% | 100,00% | 100,00% | 100,00% |
| scuotere | NR | 96,51% | 97,50% | 95,65% | 92,50% | 93,75% | 91,67% | 100,00% | 100,00% | 100,00% | 100,00% | 100,00% | 100,00% |
| scusare | NR | 97,65% | 97,37% | 97,87% | 94,74% | 93,75% | 95,45% | 100,00% | 100,00% | 100,00% | 100,00% | 100,00% | 100,00% |
| scusarsi | R | 97,62% | 97,62% | 97,62% | 95,12% | 95,00% | 95,24% | 100,00% | 100,00% | 100,00% | 100,00% | 100,00% | 100,00% |
| segare | NR | 95,29% | 95,00% | 95,56% | 89,47% | 87,50% | 90,91% | 100,00% | 100,00% | 100,00% | 100,00% | 100,00% | 100,00% |
| sfidare | NR | 94,32% | 95,35% | 93,33% | 100,00% | 100,00% | 100,00% | 95,00% | 100,00% | 90,91% | 81,82% | 80,00% | 83,33% |
| sganciare | NR | 93,75% | 95,35% | 91,89% | 91,18% | 89,47% | 93,33% | 95,83% | 100,00% | 90,91% | 95,45% | 100,00% | 90,91% |
| slegarsi | R | 95,24% | 93,02% | 97,56% | 100,00% | 100,00% | 100,00% | 100,00% | 100,00% | 100,00% | 78,95% | 70,00% | 88,89% |
| soffrire | NR | 96,55% | 97,87% | 95,00% | 95,35% | 95,83% | 94,74% | 95,45% | 100,00% | 90,00% | 100,00% | 100,00% | 100,00% |
| sognare | NR | 100,00% | 100,00% | 100,00% | 100,00% | 100,00% | 100,00% | 100,00% | 100,00% | 100,00% | 100,00% | 100,00% | 100,00% |
| sollevare | NR | 98,81% | 97,78% | 100,00% | 100,00% | 100,00% | 100,00% | 100,00% | 100,00% | 100,00% | 95,00% | 90,00% | 100,00% |
| sospettare | NR | 99,06% | 98,15% | 100,00% | 98,18% | 96,55% | 100,00% | 100,00% | 100,00% | 100,00% | 100,00% | 100,00% | 100,00% |
| spalmare | NR | 100,00% | 100,00% | 100,00% | 100,00% | 100,00% | 100,00% | 100,00% | 100,00% | 100,00% | 100,00% | 100,00% | 100,00% |
| spaventare | NR | 98,77% | 97,67% | 100,00% | 100,00% | 100,00% | 100,00% | 100,00% | 100,00% | 100,00% | 96,00% | 93,75% | 100,00% |
| spaventarsi | R | 97,56% | 95,12% | 100,00% | 97,44% | 95,00% | 100,00% | 100,00% | 100,00% | 100,00% | 95,24% | 90,00% | 100,00% |
| spazzolarsi | R | 97,59% | 100,00% | 94,74% | 100,00% | 100,00% | 100,00% | 96,00% | 100,00% | 91,67% | 95,24% | 100,00% | 90,00% |
| sperare | NR | 100,00% | 100,00% | 100,00% | 100,00% | 100,00% | 100,00% | 100,00% | 100,00% | 100,00% | 100,00% | 100,00% | 100,00% |
| spezzare | NR | 100,00% | 100,00% | 100,00% | 100,00% | 100,00% | 100,00% | 100,00% | 100,00% | 100,00% | 100,00% | 100,00% | 100,00% |
| spingere | NR | 98,80% | 100,00% | 97,50% | 96,97% | 100,00% | 94,74% | 100,00% | 100,00% | 100,00% | 100,00% | 100,00% | 100,00% |
| spremere | NR | 100,00% | 100,00% | 100,00% | 100,00% | 100,00% | 100,00% | 100,00% | 100,00% | 100,00% | 100,00% | 100,00% | 100,00% |
| spruzzarsi | R | 96,47% | 100,00% | 92,31% | 97,44% | 100,00% | 94,12% | 95,65% | 100,00% | 90,91% | 95,65% | 100,00% | 90,91% |
| staccare | NR | 96,30% | 95,24% | 97,44% | 100,00% | 100,00% | 100,00% | 100,00% | 100,00% | 100,00% | 85,00% | 80,00% | 90,00% |
| stringersi | R | 100,00% | 100,00% | 100,00% | 100,00% | 100,00% | 100,00% | 100,00% | 100,00% | 100,00% | 100,00% | 100,00% | 100,00% |
| strizzare | NR | 95,10% | 91,84% | 98,11% | 90,74% | 85,19% | 96,30% | 100,00% | 100,00% | 100,00% | 100,00% | 100,00% | 100,00% |
| strofinare | NR | 97,56% | 97,37% | 97,73% | 94,59% | 93,75% | 95,24% | 100,00% | 100,00% | 100,00% | 100,00% | 100,00% | 100,00% |
| suicidarsi | R | 96,59% | 95,00% | 97,92% | 92,50% | 87,50% | 95,83% | 100,00% | 100,00% | 100,00% | 100,00% | 100,00% | 100,00% |
| svitare | NR | 96,43% | 97,73% | 95,00% | 96,97% | 100,00% | 94,12% | 100,00% | 100,00% | 100,00% | 92,31% | 93,33% | 90,91% |
| temere | NR | 99,04% | 98,08% | 100,00% | 98,18% | 96,55% | 100,00% | 100,00% | 100,00% | 100,00% | 100,00% | 100,00% | 100,00% |
| terrorizzare | NR | 87,50% | 93,33% | 81,40% | 95,56% | 100,00% | 90,48% | 95,24% | 100,00% | 90,91% | 63,64% | 72,73% | 54,55% |
| terrorizzarsi | R | 100,00% | 100,00% | 100,00% | 100,00% | 100,00% | 100,00% | 100,00% | 100,00% | 100,00% | 100,00% | 100,00% | 100,00% |
| tirare | NR | 100,00% | 100,00% | 100,00% | 100,00% | 100,00% | 100,00% | 100,00% | 100,00% | 100,00% | 100,00% | 100,00% | 100,00% |
| toccare | NR | 100,00% | 100,00% | 100,00% | 100,00% | 100,00% | 100,00% | 100,00% | 100,00% | 100,00% | 100,00% | 100,00% | 100,00% |
| torturare | NR | 91,21% | 93,62% | 88,64% | 95,74% | 96,00% | 95,45% | 90,91% | 100,00% | 81,82% | 81,82% | 81,82% | 81,82% |
| tradire | NR | 98,08% | 98,15% | 98,00% | 96,30% | 96,55% | 96,00% | 100,00% | 100,00% | 100,00% | 100,00% | 100,00% | 100,00% |
| vendicarsi | R | 96,47% | 97,62% | 95,35% | 92,68% | 95,00% | 90,48% | 100,00% | 100,00% | 100,00% | 100,00% | 100,00% | 100,00% |
| vergognarsi | R | 98,81% | 100,00% | 97,73% | 100,00% | 100,00% | 100,00% | 95,83% | 100,00% | 92,31% | 100,00% | 100,00% | 100,00% |
| versare | NR | 98,84% | 100,00% | 97,37% | 97,44% | 100,00% | 93,75% | 100,00% | 100,00% | 100,00% | 100,00% | 100,00% | 100,00% |
| vestirsi | R | 100,00% | 100,00% | 100,00% | 100,00% | 100,00% | 100,00% | 100,00% | 100,00% | 100,00% | 100,00% | 100,00% | 100,00% |

**Supplementary Table 2** - showing, for each verb, the percentage of children that stated to know the verb (familiarity). The percentages are reported for each age range (8-11 ALL, 12-15 ALL, 16-19 ALL), for males and females within each age range(8-11 females, 8-11 males; 12-15 females, 12-15 males; 16-19 females, 16-19 males), for males and females within the whole sample (females ALL, males ALL) and for the whole sample (ALL).
