# Supplementary material for: Age-Dependent Changes of Thinking about Verbs
Source: Front Behav Neurosci. 2017 Mar 14;11:40. doi: 10.3389/fnbeh.2017.00040 (PMC5348498; doi:10.3389/fnbeh.2017.00040)
Supplement: Supplementary file 3 [file Table3.DOCX]

**Supplementary Table 3 - Imageability**

| **Imageability** | | **age range -> 8-19 (0VERALL SAMPLE)** | | | | | | **age range -> 8-11** | | | | | | **age range -> 12-15** | | | | | | **age range -> 16-19** | | | | | |
| --- | --- | --- | --- | --- | --- | --- | --- | --- | --- | --- | --- | --- | --- | --- | --- | --- | --- | --- | --- | --- | --- | --- | --- | --- | --- |
|  | R -NR | ALL | | F | | M | | ALL | | F | | M | | ALL | | F | | M | | ALL | | F | | M | |
|  |  | mean | sd | mean | sd | mean | sd | mean | sd | mean | sd | mean | sd | mean | sd | mean | sd | mean | sd | mean | sd | mean | sd | mean | sd |
| abbottonarsi | NR | 1,13 | 2,13 | 0,82 | 1,32 | 1,43 | 2,69 | 1,26 | 2,28 | 0,76 | 1,20 | 1,72 | 2,93 | 1,00 | 2,22 | 0,50 | 0,52 | 1,46 | 3,02 | 1,05 | 1,81 | 1,30 | 2,00 | 0,78 | 1,64 |
| afferrare | NR | 1,16 | 2,04 | 0,49 | 0,98 | 1,69 | 2,47 | 1,12 | 1,77 | 0,55 | 1,21 | 1,39 | 1,95 | 1,22 | 1,89 | 0,64 | 1,08 | 1,85 | 2,38 | 1,16 | 2,71 | 0,20 | 0,42 | 2,22 | 3,73 |
| affettare | NR | 2,11 | 3,07 | 1,83 | 2,65 | 2,42 | 3,50 | 2,14 | 3,12 | 1,65 | 2,33 | 2,74 | 3,86 | 1,65 | 2,32 | 1,90 | 2,81 | 1,40 | 1,84 | 2,64 | 3,91 | 2,29 | 3,68 | 3,00 | 4,40 |
| aggrapparsi | R | 2,60 | 2,74 | 2,81 | 2,94 | 2,32 | 2,47 | 2,31 | 2,72 | 2,65 | 3,11 | 1,81 | 2,01 | 3,08 | 2,93 | 3,46 | 3,07 | 2,64 | 2,84 | 2,59 | 2,61 | 2,42 | 2,54 | 2,80 | 2,82 |
| allacciarsi | R | 1,62 | 2,30 | 1,74 | 2,28 | 1,46 | 2,36 | 1,89 | 2,79 | 1,77 | 2,76 | 2,07 | 2,91 | 1,64 | 2,08 | 2,00 | 2,16 | 1,25 | 2,01 | 1,10 | 1,41 | 1,42 | 1,38 | 0,63 | 1,41 |
| amare | NR | 3,20 | 3,58 | 2,72 | 3,10 | 3,81 | 4,08 | 2,92 | 3,56 | 2,82 | 3,32 | 3,07 | 4,01 | 3,44 | 3,70 | 2,00 | 2,16 | 5,00 | 4,43 | 3,40 | 3,63 | 3,36 | 3,67 | 3,44 | 3,81 |
| amarsi | R | 3,73 | 4,04 | 3,63 | 4,06 | 3,83 | 4,07 | 4,24 | 4,32 | 3,90 | 4,45 | 4,55 | 4,27 | 3,14 | 3,93 | 3,33 | 4,23 | 2,89 | 3,72 | 3,26 | 3,56 | 3,44 | 3,24 | 3,10 | 3,98 |
| ammalarsi | R | 3,19 | 3,78 | 2,67 | 3,36 | 3,73 | 4,12 | 3,55 | 3,95 | 2,74 | 3,39 | 4,38 | 4,36 | 3,63 | 3,98 | 2,93 | 3,71 | 4,38 | 4,27 | 1,87 | 2,85 | 2,18 | 3,09 | 1,58 | 2,71 |
| annodare | NR | 1,84 | 2,60 | 1,84 | 2,66 | 1,84 | 2,56 | 1,55 | 2,59 | 1,54 | 2,52 | 1,56 | 2,72 | 3,00 | 3,01 | 2,86 | 3,35 | 3,15 | 2,73 | 1,17 | 1,58 | 1,27 | 1,68 | 1,08 | 1,55 |
| annoiarsi | R | 2,69 | 3,57 | 2,79 | 3,68 | 2,60 | 3,51 | 3,43 | 4,08 | 3,94 | 4,33 | 3,00 | 3,92 | 1,79 | 2,69 | 1,64 | 2,29 | 1,92 | 3,07 | 2,26 | 3,19 | 1,89 | 3,22 | 2,60 | 3,31 |
| apparecchiare | NR | 1,23 | 2,21 | 1,33 | 2,66 | 1,13 | 1,63 | 0,97 | 2,07 | 1,27 | 2,84 | 0,71 | 1,05 | 1,79 | 2,80 | 1,85 | 3,41 | 1,73 | 2,00 | 1,04 | 1,67 | 0,93 | 1,54 | 1,18 | 1,89 |
| appendere | NR | 1,42 | 2,61 | 1,02 | 2,16 | 1,83 | 2,98 | 1,33 | 2,59 | 1,00 | 2,53 | 1,68 | 2,67 | 1,58 | 3,13 | 0,58 | 1,44 | 2,58 | 4,03 | 1,40 | 2,04 | 1,60 | 2,12 | 1,20 | 2,04 |
| applaudire | NR | 1,15 | 2,16 | 1,61 | 2,81 | 0,74 | 1,26 | 1,16 | 2,06 | 1,65 | 2,76 | 0,76 | 1,18 | 0,92 | 1,71 | 1,08 | 2,19 | 0,77 | 1,17 | 1,44 | 2,91 | 2,22 | 3,73 | 0,67 | 1,66 |
| arrabbiarsi | R | 2,35 | 3,07 | 2,51 | 3,33 | 2,18 | 2,79 | 3,13 | 3,37 | 3,47 | 3,80 | 2,82 | 3,03 | 1,92 | 2,56 | 2,15 | 3,16 | 1,67 | 1,83 | 1,80 | 3,06 | 1,87 | 2,97 | 1,70 | 3,33 |
| avvitare | NR | 2,19 | 2,99 | 2,12 | 2,78 | 2,24 | 3,18 | 2,00 | 2,87 | 2,54 | 3,02 | 1,65 | 2,80 | 2,13 | 2,83 | 1,17 | 1,47 | 3,08 | 3,55 | 2,65 | 3,53 | 2,88 | 3,72 | 2,44 | 3,57 |
| avvolgersi | R | 2,90 | 3,36 | 3,14 | 3,28 | 2,61 | 3,47 | 2,94 | 3,77 | 3,60 | 4,08 | 2,31 | 3,46 | 3,17 | 3,53 | 3,54 | 3,33 | 2,73 | 3,88 | 2,58 | 2,65 | 2,33 | 2,23 | 3,00 | 3,35 |
| bussare | NR | 1,01 | 1,93 | 0,68 | 1,19 | 1,33 | 2,42 | 0,87 | 1,73 | 0,50 | 0,71 | 1,20 | 2,26 | 1,25 | 2,36 | 0,50 | 0,67 | 2,00 | 3,16 | 1,00 | 1,81 | 1,20 | 2,04 | 0,80 | 1,62 |
| calmare | NR | 2,48 | 3,14 | 2,34 | 3,22 | 2,59 | 3,11 | 1,76 | 2,77 | 2,07 | 2,84 | 1,57 | 2,78 | 3,04 | 3,19 | 3,62 | 4,01 | 2,50 | 2,21 | 3,11 | 3,60 | 1,10 | 2,08 | 5,33 | 3,71 |
| calmarsi | R | 2,25 | 3,04 | 2,50 | 3,25 | 1,98 | 2,82 | 2,12 | 2,89 | 2,23 | 3,10 | 2,00 | 2,71 | 3,36 | 3,51 | 3,31 | 3,43 | 3,44 | 3,84 | 1,50 | 2,73 | 2,18 | 3,52 | 0,92 | 1,80 |
| cancellare | NR | 1,18 | 2,17 | 1,13 | 2,06 | 1,24 | 2,30 | 1,19 | 2,10 | 0,97 | 1,65 | 1,46 | 2,57 | 1,46 | 2,83 | 1,64 | 3,03 | 1,25 | 2,70 | 0,88 | 1,39 | 0,91 | 1,64 | 0,85 | 1,21 |
| confessare | NR | 3,94 | 3,81 | 4,76 | 4,27 | 2,97 | 2,94 | 3,40 | 3,61 | 4,78 | 4,28 | 1,94 | 1,95 | 4,72 | 3,69 | 5,85 | 4,20 | 3,50 | 2,71 | 3,92 | 4,19 | 3,80 | 4,38 | 4,10 | 4,12 |
| confondersi | R | 3,07 | 2,98 | 3,03 | 2,82 | 3,12 | 3,16 | 3,21 | 3,29 | 2,58 | 2,91 | 3,80 | 3,58 | 2,68 | 2,70 | 3,17 | 2,52 | 2,23 | 2,89 | 3,32 | 2,77 | 3,78 | 3,15 | 2,90 | 2,47 |
| controllare | NR | 2,05 | 2,66 | 2,21 | 3,03 | 1,91 | 2,35 | 1,40 | 2,16 | 1,88 | 2,83 | 1,08 | 1,56 | 2,92 | 2,76 | 3,31 | 3,22 | 2,54 | 2,26 | 2,21 | 3,22 | 1,22 | 2,95 | 3,10 | 3,35 |
| controllarsi | R | 3,56 | 3,49 | 3,45 | 3,54 | 3,68 | 3,48 | 3,08 | 3,64 | 2,59 | 3,39 | 3,75 | 3,97 | 3,19 | 2,48 | 3,90 | 2,69 | 2,55 | 2,21 | 4,95 | 3,92 | 5,25 | 4,46 | 4,73 | 3,69 |
| credere | NR | 2,36 | 2,90 | 2,79 | 3,30 | 1,98 | 2,46 | 2,39 | 3,02 | 2,50 | 3,26 | 2,30 | 2,90 | 2,42 | 3,09 | 3,58 | 3,90 | 1,25 | 1,36 | 2,22 | 2,46 | 2,33 | 2,65 | 2,11 | 2,42 |
| decidere | NR | 3,33 | 3,34 | 3,18 | 2,86 | 3,48 | 3,81 | 3,02 | 3,25 | 2,92 | 2,95 | 3,15 | 3,65 | 3,32 | 3,80 | 3,33 | 3,24 | 3,30 | 4,42 | 3,91 | 3,19 | 3,64 | 2,54 | 4,17 | 3,79 |
| deludere | NR | 4,04 | 3,63 | 4,60 | 3,69 | 3,35 | 3,47 | 3,53 | 4,02 | 4,43 | 3,74 | 2,41 | 4,18 | 4,43 | 3,15 | 4,42 | 3,92 | 4,45 | 2,21 | 4,52 | 3,40 | 5,08 | 3,65 | 3,78 | 3,07 |
| deprimersi | R | 4,37 | 3,68 | 4,51 | 3,32 | 4,20 | 4,11 | 3,04 | 3,62 | 2,93 | 2,97 | 3,17 | 4,39 | 4,80 | 3,58 | 5,27 | 3,10 | 4,22 | 4,21 | 5,74 | 3,41 | 5,90 | 3,38 | 5,56 | 3,64 |
| desiderare | NR | 1,77 | 2,90 | 2,04 | 3,00 | 1,50 | 2,79 | 1,63 | 3,08 | 1,74 | 3,13 | 1,50 | 3,08 | 2,11 | 2,98 | 2,29 | 2,97 | 1,92 | 3,09 | 1,71 | 2,48 | 2,45 | 2,91 | 1,08 | 1,93 |
| dimenticare | NR | 3,24 | 3,58 | 3,96 | 3,67 | 2,43 | 3,34 | 3,39 | 3,47 | 4,59 | 3,50 | 2,32 | 3,15 | 3,38 | 3,84 | 3,46 | 4,03 | 3,27 | 3,80 | 2,88 | 3,62 | 3,67 | 3,70 | 1,70 | 3,33 |
| dipingere | NR | 1,33 | 2,57 | 1,04 | 2,26 | 1,63 | 2,86 | 1,13 | 2,33 | 0,70 | 1,34 | 1,64 | 3,08 | 1,78 | 3,15 | 1,50 | 3,03 | 2,08 | 3,38 | 1,27 | 2,43 | 1,44 | 3,28 | 1,15 | 1,77 |
| disegnare | NR | 1,60 | 2,70 | 1,51 | 2,49 | 1,68 | 2,91 | 1,25 | 2,29 | 1,71 | 3,05 | 0,89 | 1,45 | 1,77 | 3,31 | 0,82 | 1,08 | 2,73 | 4,45 | 2,00 | 2,65 | 2,00 | 2,79 | 2,00 | 2,65 |
| dispiacersi | R | 3,51 | 3,75 | 3,09 | 3,32 | 4,06 | 4,22 | 3,42 | 4,00 | 2,46 | 3,04 | 4,63 | 4,76 | 2,29 | 3,00 | 2,20 | 2,25 | 2,43 | 4,04 | 4,75 | 3,55 | 5,27 | 3,98 | 4,11 | 3,06 |
| fallire | NR | 4,78 | 3,72 | 5,41 | 3,53 | 4,08 | 3,86 | 5,15 | 4,30 | 6,75 | 3,96 | 3,72 | 4,18 | 4,58 | 3,73 | 5,00 | 3,29 | 4,09 | 4,30 | 4,48 | 2,87 | 4,33 | 2,94 | 4,70 | 2,91 |
| fingere | NR | 3,28 | 3,57 | 3,37 | 3,57 | 3,20 | 3,61 | 1,88 | 2,63 | 1,58 | 2,23 | 2,05 | 2,85 | 4,46 | 3,74 | 5,62 | 3,99 | 3,31 | 3,22 | 4,10 | 4,06 | 2,60 | 2,95 | 5,60 | 4,60 |
| fotografarsi | R | 1,03 | 2,00 | 0,70 | 1,35 | 1,41 | 2,52 | 1,00 | 1,90 | 0,76 | 1,51 | 1,27 | 2,27 | 0,50 | 0,95 | 0,40 | 0,97 | 0,60 | 0,97 | 1,92 | 3,12 | 0,88 | 1,36 | 3,60 | 4,51 |
| gettare | NR | 1,06 | 1,86 | 0,68 | 1,20 | 1,37 | 2,22 | 0,57 | 1,14 | 0,50 | 1,09 | 0,61 | 1,20 | 1,27 | 1,80 | 1,08 | 1,44 | 1,46 | 2,15 | 1,70 | 2,70 | 0,40 | 0,97 | 3,00 | 3,27 |
| graffiarsi | R | 2,37 | 3,19 | 2,27 | 3,15 | 2,46 | 3,26 | 2,00 | 2,93 | 1,82 | 2,75 | 2,19 | 3,16 | 3,65 | 4,01 | 4,00 | 4,11 | 3,27 | 4,05 | 1,96 | 2,63 | 1,55 | 2,42 | 2,31 | 2,84 |
| grattarsi | R | 1,26 | 2,12 | 1,38 | 2,25 | 1,11 | 1,97 | 1,29 | 2,04 | 1,38 | 1,54 | 1,20 | 2,51 | 1,68 | 2,95 | 2,18 | 3,76 | 1,18 | 1,89 | 0,83 | 1,05 | 0,80 | 1,15 | 0,89 | 0,93 |
| grattugiare | NR | 1,41 | 2,19 | 1,27 | 1,99 | 1,56 | 2,41 | 1,31 | 1,95 | 1,33 | 2,10 | 1,29 | 1,82 | 1,00 | 2,08 | 0,50 | 0,97 | 1,50 | 2,76 | 2,11 | 2,78 | 1,90 | 2,38 | 2,38 | 3,38 |
| guarire | NR | 2,38 | 2,93 | 3,05 | 3,19 | 1,59 | 2,39 | 2,75 | 3,43 | 3,69 | 3,79 | 1,81 | 2,83 | 2,29 | 2,29 | 2,54 | 2,33 | 2,00 | 2,32 | 2,00 | 2,83 | 2,80 | 3,23 | 0,80 | 1,55 |
| impaurirsi | R | 2,86 | 3,27 | 2,65 | 3,00 | 3,06 | 3,53 | 2,52 | 3,21 | 2,19 | 2,90 | 2,88 | 3,55 | 4,00 | 3,44 | 3,85 | 3,00 | 4,15 | 3,95 | 2,29 | 2,97 | 2,33 | 3,16 | 2,25 | 2,96 |
| impazzire | NR | 4,74 | 4,11 | 4,98 | 4,01 | 4,43 | 4,28 | 5,14 | 4,29 | 5,00 | 4,28 | 5,36 | 4,47 | 4,71 | 4,06 | 5,23 | 3,88 | 4,09 | 4,37 | 4,10 | 3,97 | 4,64 | 3,98 | 3,50 | 4,09 |
| impressionarsi | R | 3,53 | 3,37 | 3,97 | 3,49 | 3,10 | 3,24 | 4,39 | 3,87 | 4,58 | 3,85 | 4,18 | 4,00 | 2,16 | 1,91 | 2,58 | 2,11 | 1,77 | 1,69 | 3,72 | 3,44 | 4,63 | 4,03 | 3,00 | 2,91 |
| inchiodare | NR | 2,97 | 3,31 | 3,46 | 3,31 | 2,44 | 3,27 | 2,56 | 3,07 | 3,36 | 3,05 | 1,63 | 2,89 | 3,21 | 3,68 | 4,10 | 4,01 | 2,22 | 3,19 | 3,80 | 3,51 | 2,86 | 3,39 | 4,63 | 3,62 |
| incollarsi | R | 3,39 | 3,66 | 3,51 | 3,56 | 3,26 | 3,82 | 3,23 | 3,53 | 3,82 | 3,49 | 2,50 | 3,54 | 2,84 | 3,55 | 2,89 | 3,89 | 2,80 | 3,43 | 4,83 | 4,22 | 3,33 | 3,83 | 6,33 | 4,37 |
| indicarsi | R | 2,06 | 3,10 | 1,80 | 2,80 | 2,30 | 3,36 | 1,84 | 3,04 | 1,59 | 2,50 | 2,09 | 3,52 | 2,22 | 2,94 | 2,23 | 2,83 | 2,21 | 3,14 | 2,29 | 3,47 | 1,73 | 3,50 | 2,77 | 3,52 |
| infilare | NR | 1,84 | 2,83 | 2,19 | 3,31 | 1,48 | 2,21 | 1,47 | 2,56 | 1,43 | 2,63 | 1,52 | 2,53 | 3,13 | 3,48 | 4,14 | 4,07 | 1,70 | 1,77 | 1,39 | 2,41 | 1,60 | 3,06 | 1,23 | 1,88 |
| ingelosirsi | R | 3,97 | 3,91 | 3,64 | 3,83 | 4,44 | 4,04 | 3,47 | 4,46 | 3,67 | 4,60 | 3,15 | 4,39 | 4,95 | 3,72 | 3,50 | 3,03 | 6,70 | 3,86 | 3,75 | 2,99 | 3,73 | 3,26 | 3,78 | 2,82 |
| insultare | NR | 3,78 | 3,68 | 4,48 | 4,04 | 3,03 | 3,12 | 4,16 | 4,08 | 5,00 | 4,49 | 3,41 | 3,66 | 3,83 | 3,79 | 5,58 | 4,23 | 1,91 | 2,02 | 3,27 | 3,09 | 3,07 | 3,17 | 3,55 | 3,11 |
| intrecciarsi | R | 4,76 | 3,94 | 4,87 | 3,78 | 4,64 | 4,17 | 5,21 | 4,25 | 5,85 | 3,91 | 4,69 | 4,57 | 4,48 | 3,68 | 5,45 | 3,91 | 3,40 | 3,27 | 4,43 | 3,85 | 3,50 | 3,39 | 6,29 | 4,31 |
| intristirsi | R | 3,08 | 3,14 | 3,72 | 3,47 | 2,31 | 2,53 | 3,63 | 3,42 | 5,00 | 3,72 | 2,07 | 2,27 | 3,21 | 3,12 | 4,08 | 3,48 | 2,33 | 2,57 | 2,28 | 2,75 | 2,07 | 2,63 | 2,60 | 3,03 |
| invidiare | NR | 4,65 | 4,12 | 5,16 | 4,18 | 4,03 | 4,02 | 4,03 | 4,34 | 4,88 | 4,57 | 3,07 | 3,99 | 6,13 | 3,79 | 6,75 | 3,70 | 5,45 | 3,96 | 4,08 | 3,94 | 4,20 | 3,97 | 3,90 | 4,09 |
| irritarsi | NR | 3,41 | 3,40 | 4,00 | 3,52 | 2,53 | 3,05 | 3,62 | 3,90 | 3,95 | 4,24 | 3,00 | 3,27 | 3,17 | 3,02 | 3,85 | 3,00 | 2,36 | 2,98 | 3,38 | 3,20 | 4,25 | 3,05 | 2,22 | 3,19 |
| lanciare | NR | 0,97 | 1,66 | 0,53 | 1,48 | 1,35 | 1,72 | 0,95 | 1,62 | 0,69 | 1,70 | 1,13 | 1,57 | 0,67 | 1,21 | 0,29 | 0,83 | 1,08 | 1,44 | 1,42 | 2,19 | 0,60 | 1,90 | 2,33 | 2,24 |
| lavarsi | R | 1,18 | 2,23 | 0,78 | 1,42 | 1,55 | 2,74 | 1,07 | 1,83 | 0,60 | 1,23 | 1,50 | 2,18 | 1,13 | 2,36 | 0,83 | 1,34 | 1,42 | 3,12 | 1,47 | 2,87 | 1,11 | 1,96 | 1,80 | 3,58 |
| legare | NR | 2,41 | 2,83 | 3,35 | 3,24 | 1,33 | 1,77 | 2,47 | 3,17 | 3,67 | 3,90 | 1,28 | 1,56 | 2,48 | 2,63 | 3,58 | 2,75 | 1,27 | 1,95 | 2,26 | 2,63 | 2,81 | 2,88 | 1,45 | 2,07 |
| maledire | NR | 5,33 | 4,04 | 5,59 | 4,09 | 5,11 | 4,04 | 4,42 | 4,12 | 4,23 | 4,02 | 4,55 | 4,29 | 6,57 | 3,67 | 7,57 | 3,72 | 5,57 | 3,46 | 5,10 | 4,17 | 4,60 | 3,92 | 5,60 | 4,55 |
| martellare | NR | 2,42 | 2,76 | 2,93 | 3,17 | 1,77 | 2,00 | 2,56 | 2,85 | 3,10 | 3,35 | 1,79 | 1,76 | 2,17 | 2,71 | 2,46 | 3,10 | 1,82 | 2,27 | 2,48 | 2,77 | 3,18 | 3,12 | 1,70 | 2,21 |
| mentire | NR | 4,37 | 4,02 | 4,55 | 3,96 | 4,20 | 4,11 | 5,18 | 4,39 | 5,33 | 4,14 | 5,05 | 4,70 | 3,95 | 3,77 | 3,73 | 4,13 | 4,18 | 3,57 | 3,21 | 3,29 | 4,00 | 3,50 | 2,50 | 3,10 |
| meritare | NR | 2,91 | 3,21 | 2,61 | 2,85 | 3,23 | 3,56 | 2,40 | 3,09 | 1,81 | 2,44 | 3,05 | 3,63 | 2,24 | 2,70 | 2,20 | 2,15 | 2,27 | 3,23 | 4,65 | 3,47 | 4,70 | 3,43 | 4,60 | 3,69 |
| mescolare | NR | 0,95 | 1,53 | 0,73 | 1,47 | 1,18 | 1,58 | 0,72 | 1,15 | 0,45 | 0,83 | 1,00 | 1,37 | 1,04 | 1,40 | 0,67 | 0,98 | 1,42 | 1,68 | 1,33 | 2,28 | 1,44 | 2,65 | 1,22 | 1,99 |
| morire | NR | 6,51 | 4,61 | 7,28 | 4,36 | 5,67 | 4,78 | 6,22 | 4,67 | 6,96 | 4,41 | 5,30 | 4,93 | 6,60 | 4,63 | 8,70 | 3,95 | 4,50 | 4,45 | 7,00 | 4,64 | 6,73 | 4,73 | 7,25 | 4,75 |
| obbedire | NR | 2,38 | 2,95 | 2,30 | 2,65 | 2,47 | 3,32 | 1,29 | 2,38 | 1,86 | 2,87 | 0,43 | 0,94 | 3,13 | 2,94 | 2,75 | 2,63 | 3,50 | 3,29 | 3,33 | 3,32 | 2,64 | 2,29 | 4,10 | 4,18 |
| odiare | NR | 3,99 | 3,72 | 3,84 | 3,94 | 4,11 | 3,58 | 3,32 | 3,71 | 3,87 | 4,27 | 2,96 | 3,35 | 4,25 | 3,35 | 4,57 | 3,72 | 3,93 | 3,05 | 4,95 | 4,18 | 2,67 | 3,87 | 7,00 | 3,43 |
| odiarsi | R | 4,73 | 4,16 | 5,12 | 4,17 | 4,30 | 4,16 | 3,90 | 4,17 | 5,31 | 4,15 | 2,89 | 4,00 | 5,13 | 4,18 | 5,00 | 4,45 | 5,30 | 4,03 | 5,42 | 4,12 | 5,07 | 4,23 | 6,00 | 4,09 |
| peccare | NR | 4,20 | 3,77 | 4,07 | 3,24 | 4,33 | 4,24 | 3,59 | 3,52 | 3,70 | 3,15 | 3,48 | 3,92 | 5,00 | 3,66 | 4,31 | 2,43 | 5,82 | 4,73 | 4,48 | 4,25 | 4,50 | 4,43 | 4,46 | 4,29 |
| penare | NR | 4,98 | 3,58 | 5,03 | 3,57 | 4,94 | 3,63 | 4,35 | 3,17 | 4,07 | 2,63 | 4,63 | 3,67 | 6,33 | 4,17 | 7,25 | 4,43 | 5,60 | 4,03 | 4,71 | 3,42 | 4,67 | 3,64 | 4,75 | 3,41 |
| pennellarsi | R | 3,02 | 3,75 | 2,91 | 3,73 | 3,13 | 3,80 | 2,00 | 2,88 | 1,40 | 2,10 | 2,63 | 3,45 | 4,38 | 4,42 | 5,00 | 5,04 | 3,70 | 3,77 | 4,00 | 4,25 | 4,40 | 3,95 | 3,67 | 4,64 |
| perdere | NR | 2,88 | 3,16 | 3,50 | 3,62 | 2,21 | 2,46 | 2,58 | 3,22 | 3,47 | 3,91 | 1,63 | 2,00 | 2,72 | 2,91 | 3,23 | 3,35 | 2,17 | 2,37 | 3,48 | 3,40 | 3,83 | 3,76 | 3,09 | 3,08 |
| perdonare | NR | 2,93 | 3,16 | 2,98 | 3,18 | 2,87 | 3,18 | 2,61 | 2,87 | 2,38 | 2,50 | 2,88 | 3,33 | 3,00 | 3,65 | 3,36 | 4,03 | 2,64 | 3,38 | 3,45 | 3,20 | 3,89 | 3,55 | 3,09 | 3,02 |
| perdonarsi | R | 2,78 | 2,96 | 3,16 | 3,19 | 2,41 | 2,70 | 2,68 | 2,97 | 2,79 | 3,17 | 2,56 | 2,83 | 3,17 | 3,33 | 3,58 | 3,73 | 2,75 | 2,99 | 2,44 | 2,39 | 3,43 | 2,51 | 1,67 | 2,12 |
| perseguitare | NR | 5,51 | 4,02 | 6,15 | 3,97 | 4,74 | 4,01 | 4,52 | 4,27 | 5,15 | 4,39 | 3,93 | 4,23 | 6,08 | 4,01 | 7,15 | 4,00 | 4,92 | 3,85 | 6,04 | 3,67 | 6,13 | 3,60 | 5,88 | 4,05 |
| pettinarsi | R | 1,22 | 2,26 | 0,96 | 1,83 | 1,53 | 2,66 | 0,84 | 1,72 | 0,64 | 1,29 | 1,11 | 2,18 | 0,82 | 1,87 | 0,55 | 1,21 | 1,09 | 2,39 | 2,50 | 3,15 | 2,20 | 2,90 | 2,80 | 3,52 |
| piacere | NR | 2,06 | 2,81 | 2,09 | 2,87 | 2,04 | 2,80 | 1,43 | 2,68 | 0,71 | 1,07 | 1,87 | 3,25 | 2,77 | 3,20 | 3,00 | 3,83 | 2,54 | 2,57 | 2,35 | 2,23 | 3,00 | 2,56 | 1,78 | 1,86 |
| piacersi | R | 2,73 | 3,07 | 2,95 | 3,30 | 2,51 | 2,86 | 2,66 | 3,43 | 2,65 | 3,76 | 2,67 | 3,18 | 2,48 | 2,41 | 2,64 | 2,58 | 2,33 | 2,35 | 3,15 | 3,07 | 3,90 | 3,14 | 2,40 | 2,95 |
| piangere | NR | 2,68 | 3,57 | 2,67 | 3,27 | 2,68 | 3,95 | 2,16 | 3,12 | 2,65 | 3,20 | 1,60 | 3,04 | 3,68 | 3,99 | 4,08 | 4,07 | 3,25 | 4,03 | 2,37 | 3,62 | 1,56 | 2,22 | 3,55 | 4,91 |
| piegare | NR | 1,61 | 2,55 | 1,60 | 2,60 | 1,63 | 2,53 | 1,05 | 2,15 | 1,00 | 1,98 | 1,11 | 2,40 | 1,86 | 3,00 | 2,50 | 3,87 | 1,27 | 1,95 | 2,60 | 2,64 | 2,20 | 2,30 | 3,00 | 3,02 |
| pinzare | NR | 1,37 | 2,23 | 1,00 | 2,28 | 1,62 | 2,18 | 0,68 | 1,22 | 0,00 | 0,00 | 0,95 | 1,36 | 2,19 | 2,67 | 2,31 | 3,12 | 2,08 | 2,25 | 1,37 | 2,54 | 0,11 | 0,33 | 2,50 | 3,14 |
| pizzicarsi | R | 2,66 | 3,28 | 2,68 | 3,13 | 2,64 | 3,49 | 2,08 | 2,82 | 2,57 | 3,22 | 1,44 | 2,13 | 1,65 | 1,79 | 2,20 | 2,20 | 1,10 | 1,10 | 5,64 | 4,36 | 3,71 | 4,11 | 7,57 | 3,95 |
| posare | NR | 1,90 | 2,72 | 2,00 | 2,72 | 1,79 | 2,75 | 1,03 | 2,23 | 1,92 | 3,04 | 0,31 | 0,79 | 2,57 | 2,93 | 2,75 | 3,14 | 2,33 | 2,78 | 2,39 | 2,90 | 1,43 | 1,99 | 3,89 | 3,55 |
| premere | NR | 1,40 | 1,94 | 1,52 | 1,93 | 1,25 | 1,98 | 0,70 | 1,13 | 0,91 | 1,12 | 0,36 | 1,08 | 1,79 | 2,11 | 2,08 | 2,23 | 1,50 | 2,02 | 2,19 | 2,48 | 2,18 | 2,60 | 2,20 | 2,49 |
| prendere | NR | 1,19 | 1,83 | 1,30 | 1,94 | 1,05 | 1,69 | 0,59 | 1,19 | 0,76 | 1,37 | 0,38 | 0,89 | 1,44 | 2,08 | 1,54 | 2,18 | 1,33 | 2,06 | 1,91 | 2,14 | 2,00 | 2,37 | 1,80 | 1,93 |
| preoccuparsi | R | 3,44 | 3,61 | 3,98 | 3,84 | 2,93 | 3,34 | 4,10 | 3,95 | 4,11 | 4,12 | 4,10 | 3,89 | 3,20 | 3,37 | 4,67 | 3,65 | 1,85 | 2,51 | 2,45 | 3,05 | 2,90 | 3,63 | 2,00 | 2,45 |
| punire | NR | 3,48 | 3,68 | 3,17 | 3,54 | 3,82 | 3,84 | 2,96 | 3,58 | 2,90 | 3,49 | 3,04 | 3,76 | 4,54 | 3,90 | 4,14 | 3,90 | 5,00 | 4,02 | 3,55 | 3,56 | 2,60 | 3,24 | 4,33 | 3,75 |
| punirsi | R | 4,98 | 4,01 | 5,36 | 4,16 | 4,67 | 3,90 | 3,85 | 4,08 | 3,93 | 4,37 | 3,80 | 3,99 | 5,79 | 3,75 | 6,57 | 3,98 | 5,00 | 3,46 | 6,10 | 3,82 | 5,80 | 3,85 | 6,40 | 3,98 |
| raccogliere | NR | 0,83 | 1,51 | 0,47 | 1,27 | 1,10 | 1,64 | 0,78 | 1,42 | 0,73 | 1,71 | 0,80 | 1,26 | 0,74 | 1,38 | 0,46 | 1,13 | 1,00 | 1,57 | 1,05 | 1,90 | 0,10 | 0,32 | 2,11 | 2,37 |
| rallegrarsi | R | 1,70 | 2,78 | 0,95 | 2,19 | 2,35 | 3,08 | 1,57 | 2,76 | 0,71 | 1,38 | 2,14 | 3,29 | 2,44 | 3,32 | 1,79 | 3,14 | 3,15 | 3,48 | 0,83 | 1,42 | 0,00 | 0,00 | 1,67 | 1,66 |
| rastrellare | NR | 2,29 | 2,90 | 2,19 | 2,77 | 2,42 | 3,08 | 2,03 | 3,20 | 1,79 | 3,14 | 2,33 | 3,37 | 2,45 | 2,76 | 2,36 | 2,34 | 2,55 | 3,24 | 2,55 | 2,63 | 2,67 | 2,64 | 2,40 | 2,76 |
| rattristarsi | R | 3,05 | 3,46 | 3,25 | 3,73 | 2,84 | 3,18 | 3,24 | 3,67 | 3,22 | 3,84 | 3,27 | 3,59 | 2,67 | 2,96 | 2,42 | 2,75 | 2,92 | 3,26 | 3,20 | 3,79 | 4,30 | 4,60 | 2,10 | 2,56 |
| ridere | NR | 0,77 | 1,81 | 0,78 | 1,53 | 0,75 | 2,10 | 0,94 | 2,20 | 1,17 | 1,79 | 0,72 | 2,59 | 0,50 | 1,38 | 0,42 | 1,00 | 0,58 | 1,73 | 0,77 | 1,56 | 0,63 | 1,54 | 1,00 | 1,63 |
| rifiutare | NR | 2,53 | 2,99 | 2,74 | 2,95 | 2,32 | 3,05 | 2,52 | 3,16 | 3,04 | 3,13 | 1,90 | 3,16 | 2,16 | 2,81 | 2,38 | 3,16 | 2,00 | 2,68 | 2,86 | 2,87 | 2,30 | 2,50 | 3,33 | 3,17 |
| rilassarsi | R | 1,77 | 2,63 | 1,95 | 2,79 | 1,56 | 2,45 | 1,39 | 2,21 | 2,20 | 2,88 | 0,72 | 1,13 | 2,20 | 2,71 | 2,62 | 3,12 | 1,75 | 2,22 | 1,83 | 3,07 | 1,13 | 2,36 | 3,00 | 3,87 |
| rubare | NR | 4,51 | 4,36 | 4,13 | 4,21 | 4,93 | 4,54 | 4,14 | 4,44 | 3,42 | 4,00 | 4,96 | 4,85 | 6,27 | 4,19 | 6,62 | 4,43 | 5,78 | 4,02 | 3,52 | 4,02 | 2,56 | 3,21 | 4,25 | 4,54 |
| ruotare | NR | 2,33 | 3,16 | 2,17 | 2,76 | 2,55 | 3,66 | 2,46 | 3,38 | 2,65 | 3,23 | 2,14 | 3,72 | 1,60 | 2,04 | 1,91 | 2,21 | 1,22 | 1,86 | 3,00 | 3,80 | 1,00 | 1,41 | 4,75 | 4,43 |
| sanguinare | NR | 2,53 | 3,21 | 3,43 | 3,20 | 1,58 | 2,98 | 2,45 | 3,01 | 3,52 | 3,12 | 1,16 | 2,36 | 2,64 | 3,42 | 3,36 | 3,50 | 1,91 | 3,33 | 2,57 | 3,69 | 3,17 | 3,54 | 2,13 | 3,98 |
| sbagliare | NR | 2,23 | 2,58 | 2,57 | 2,62 | 1,84 | 2,50 | 1,47 | 2,36 | 2,05 | 2,82 | 0,75 | 1,39 | 3,00 | 2,80 | 2,54 | 2,07 | 3,55 | 3,50 | 2,67 | 2,42 | 3,55 | 2,77 | 1,70 | 1,57 |
| sbattere | NR | 1,91 | 2,40 | 1,83 | 2,14 | 2,00 | 2,73 | 1,53 | 2,21 | 1,65 | 2,21 | 1,33 | 2,29 | 1,96 | 2,32 | 1,77 | 1,64 | 2,17 | 2,95 | 2,48 | 2,76 | 2,25 | 2,56 | 2,73 | 3,07 |
| scarabocchiare | NR | 1,29 | 2,29 | 1,21 | 2,55 | 1,35 | 2,08 | 1,28 | 2,39 | 1,69 | 3,24 | 1,00 | 1,62 | 1,33 | 2,20 | 1,50 | 2,43 | 1,17 | 2,04 | 1,25 | 2,31 | 0,10 | 0,32 | 2,40 | 2,88 |
| schiacciare | NR | 1,52 | 2,25 | 1,13 | 1,92 | 1,82 | 2,45 | 0,88 | 1,44 | 0,87 | 1,13 | 0,88 | 1,62 | 2,29 | 2,85 | 2,14 | 2,71 | 2,43 | 3,08 | 1,71 | 2,35 | 0,10 | 0,32 | 3,18 | 2,44 |
| sconfiggere | NR | 2,54 | 3,16 | 3,02 | 3,25 | 2,04 | 3,02 | 2,41 | 3,28 | 2,69 | 3,34 | 2,09 | 3,25 | 3,15 | 3,34 | 3,93 | 3,20 | 2,31 | 3,40 | 2,13 | 2,72 | 2,64 | 3,17 | 1,69 | 2,32 |
| scoraggiarsi | R | 4,32 | 3,36 | 4,27 | 3,31 | 4,38 | 3,47 | 5,30 | 3,75 | 5,21 | 3,26 | 5,38 | 4,35 | 4,04 | 2,99 | 4,62 | 3,10 | 3,36 | 2,84 | 3,41 | 3,05 | 3,00 | 3,37 | 4,13 | 2,42 |
| scrivere | NR | 0,99 | 2,18 | 1,00 | 2,17 | 0,98 | 2,20 | 0,93 | 2,21 | 0,59 | 1,27 | 1,32 | 2,93 | 1,08 | 2,29 | 1,21 | 2,91 | 0,91 | 1,22 | 1,04 | 2,07 | 1,82 | 2,86 | 0,38 | 0,65 |
| scuotere | NR | 1,46 | 2,46 | 0,97 | 2,13 | 1,89 | 2,66 | 0,68 | 1,53 | 0,13 | 0,35 | 1,05 | 1,89 | 1,63 | 2,56 | 2,00 | 3,21 | 1,23 | 1,64 | 2,74 | 3,21 | 0,80 | 1,14 | 4,89 | 3,44 |
| scusare | NR | 2,42 | 3,35 | 2,14 | 3,47 | 2,65 | 3,27 | 1,58 | 2,68 | 1,47 | 2,33 | 1,67 | 2,96 | 2,50 | 3,19 | 2,42 | 3,82 | 2,57 | 2,68 | 3,76 | 4,19 | 2,80 | 4,52 | 4,64 | 3,88 |
| scusarsi | R | 2,74 | 3,37 | 3,37 | 3,77 | 2,12 | 2,82 | 2,05 | 2,71 | 2,26 | 2,81 | 1,85 | 2,68 | 3,29 | 3,90 | 3,83 | 4,37 | 2,75 | 3,47 | 3,47 | 3,75 | 4,90 | 4,31 | 1,89 | 2,32 |
| segare | NR | 2,22 | 2,96 | 1,97 | 3,06 | 2,44 | 2,89 | 1,44 | 2,48 | 1,00 | 1,71 | 1,75 | 2,90 | 3,32 | 3,45 | 4,00 | 4,04 | 2,64 | 2,73 | 2,00 | 2,60 | 0,50 | 0,71 | 3,67 | 2,96 |
| sfidare | NR | 3,41 | 3,35 | 4,29 | 3,64 | 2,55 | 2,82 | 2,87 | 3,18 | 3,67 | 3,50 | 2,00 | 2,60 | 3,16 | 3,13 | 4,56 | 3,50 | 1,90 | 2,23 | 5,06 | 3,64 | 5,88 | 4,16 | 4,40 | 3,24 |
| sganciare | NR | 2,75 | 2,84 | 2,37 | 2,72 | 3,21 | 2,96 | 1,97 | 2,83 | 0,88 | 1,32 | 3,29 | 3,60 | 3,22 | 2,75 | 3,92 | 3,12 | 2,30 | 1,95 | 3,38 | 2,82 | 2,82 | 2,82 | 4,00 | 2,83 |
| slegarsi | R | 2,04 | 2,92 | 2,18 | 2,95 | 1,90 | 2,92 | 1,49 | 2,13 | 1,43 | 1,80 | 1,55 | 2,48 | 2,96 | 3,90 | 3,42 | 3,90 | 2,50 | 4,01 | 2,07 | 2,79 | 2,29 | 3,59 | 1,88 | 2,10 |
| soffrire | NR | 3,62 | 3,63 | 3,89 | 3,97 | 3,29 | 3,19 | 3,85 | 3,95 | 4,57 | 4,12 | 2,94 | 3,62 | 3,19 | 3,70 | 3,67 | 4,64 | 2,56 | 1,94 | 3,59 | 3,00 | 2,73 | 2,69 | 4,45 | 3,17 |
| sognare | NR | 2,41 | 2,94 | 2,78 | 3,03 | 1,97 | 2,80 | 2,31 | 3,00 | 2,36 | 3,13 | 2,24 | 2,93 | 1,84 | 2,41 | 2,77 | 2,83 | 0,83 | 1,34 | 3,29 | 3,30 | 3,64 | 3,17 | 2,90 | 3,57 |
| sollevare | NR | 2,01 | 2,84 | 2,11 | 2,88 | 1,90 | 2,83 | 1,82 | 2,70 | 1,72 | 2,56 | 1,95 | 2,95 | 2,45 | 3,76 | 3,30 | 4,08 | 1,60 | 3,41 | 2,00 | 2,00 | 1,89 | 1,96 | 2,10 | 2,13 |
| sospettare | NR | 2,88 | 3,17 | 3,08 | 3,18 | 2,67 | 3,17 | 2,57 | 3,17 | 2,75 | 3,25 | 2,38 | 3,14 | 4,04 | 3,14 | 4,43 | 2,93 | 3,62 | 3,43 | 2,25 | 2,97 | 2,18 | 3,03 | 2,31 | 3,04 |
| spalmare | NR | 0,86 | 1,51 | 0,59 | 1,34 | 1,07 | 1,61 | 0,74 | 1,50 | 0,15 | 0,38 | 1,04 | 1,77 | 1,08 | 1,60 | 1,36 | 1,95 | 0,75 | 1,06 | 0,79 | 1,44 | 0,10 | 0,32 | 1,56 | 1,81 |
| spaventare | NR | 1,95 | 2,54 | 2,52 | 3,01 | 1,32 | 1,69 | 1,55 | 2,60 | 2,40 | 3,50 | 0,83 | 1,20 | 2,83 | 2,57 | 3,42 | 2,68 | 2,18 | 2,40 | 1,67 | 2,30 | 1,93 | 2,76 | 1,22 | 1,20 |
| spaventarsi | R | 3,04 | 3,55 | 3,03 | 3,44 | 3,05 | 3,68 | 2,34 | 2,67 | 2,21 | 2,35 | 2,47 | 3,03 | 2,41 | 3,00 | 2,64 | 2,62 | 2,18 | 3,46 | 5,05 | 4,76 | 5,22 | 5,31 | 4,91 | 4,53 |
| spazzolarsi | R | 1,19 | 1,92 | 0,89 | 1,27 | 1,56 | 2,49 | 0,81 | 1,94 | 0,57 | 0,93 | 1,13 | 2,78 | 1,63 | 2,22 | 1,23 | 1,74 | 2,09 | 2,70 | 1,35 | 1,39 | 1,09 | 1,14 | 1,67 | 1,66 |
| sperare | NR | 3,04 | 3,39 | 3,11 | 3,38 | 2,95 | 3,44 | 2,32 | 3,00 | 2,73 | 3,38 | 1,75 | 2,35 | 3,33 | 3,48 | 3,25 | 3,62 | 3,42 | 3,50 | 3,91 | 3,78 | 3,67 | 3,34 | 4,18 | 4,35 |
| spezzare | NR | 1,51 | 2,51 | 1,47 | 2,82 | 1,53 | 2,26 | 1,15 | 2,25 | 1,33 | 2,99 | 1,04 | 1,71 | 2,07 | 3,04 | 1,71 | 3,07 | 2,46 | 3,07 | 1,42 | 2,14 | 1,33 | 2,35 | 1,50 | 2,07 |
| spingere | NR | 1,98 | 2,92 | 2,35 | 3,08 | 1,56 | 2,72 | 2,28 | 3,45 | 3,00 | 3,59 | 1,72 | 3,32 | 1,96 | 2,63 | 2,62 | 3,20 | 1,18 | 1,54 | 1,62 | 2,52 | 1,56 | 2,45 | 1,70 | 2,75 |
| spremere | NR | 2,52 | 3,18 | 3,33 | 3,67 | 1,60 | 2,22 | 2,37 | 3,02 | 3,12 | 3,67 | 1,67 | 2,11 | 3,04 | 3,88 | 4,77 | 4,60 | 1,17 | 1,53 | 2,20 | 2,65 | 2,33 | 2,41 | 2,00 | 3,09 |
| spruzzarsi | R | 2,13 | 2,49 | 2,09 | 2,46 | 2,19 | 2,56 | 1,63 | 2,54 | 1,73 | 2,76 | 1,50 | 2,28 | 2,18 | 2,54 | 1,92 | 2,15 | 2,50 | 3,03 | 2,95 | 2,21 | 2,92 | 2,11 | 3,00 | 2,45 |
| staccare | NR | 1,46 | 2,21 | 1,38 | 2,22 | 1,55 | 2,23 | 1,24 | 2,34 | 1,00 | 2,08 | 1,50 | 2,64 | 1,74 | 1,84 | 1,75 | 1,82 | 1,73 | 1,95 | 1,59 | 2,43 | 1,75 | 3,11 | 1,44 | 1,81 |
| stringersi | R | 2,04 | 2,65 | 2,49 | 2,66 | 1,58 | 2,58 | 1,71 | 2,71 | 1,86 | 2,63 | 1,56 | 2,83 | 2,96 | 2,86 | 3,50 | 2,85 | 2,33 | 2,87 | 1,78 | 2,04 | 2,90 | 2,18 | 0,92 | 1,50 |
| strizzare | NR | 2,07 | 3,02 | 2,56 | 3,43 | 1,65 | 2,57 | 1,82 | 3,05 | 1,87 | 3,11 | 1,77 | 3,05 | 2,78 | 3,02 | 4,07 | 3,47 | 1,38 | 1,61 | 1,76 | 2,95 | 1,88 | 3,83 | 1,69 | 2,43 |
| strofinare | NR | 1,43 | 1,93 | 1,08 | 1,95 | 1,72 | 1,89 | 1,11 | 1,55 | 0,47 | 0,83 | 1,60 | 1,79 | 1,72 | 2,54 | 1,83 | 2,89 | 1,62 | 2,29 | 1,60 | 1,67 | 1,10 | 1,60 | 2,10 | 1,66 |
| suicidarsi | R | 6,55 | 4,74 | 7,76 | 4,43 | 5,57 | 4,81 | 4,27 | 4,48 | 5,50 | 4,99 | 3,52 | 4,08 | 7,46 | 4,73 | 9,07 | 3,97 | 5,86 | 5,01 | 9,50 | 3,03 | 9,10 | 3,03 | 9,90 | 3,14 |
| svitare | NR | 1,83 | 2,71 | 2,56 | 3,11 | 1,00 | 1,89 | 2,03 | 3,25 | 3,13 | 3,81 | 0,94 | 2,17 | 1,60 | 2,47 | 2,46 | 3,15 | 0,67 | 0,78 | 1,79 | 2,21 | 2,00 | 2,15 | 1,50 | 2,37 |
| temere | NR | 3,48 | 3,46 | 3,24 | 3,13 | 3,71 | 3,76 | 3,19 | 3,64 | 2,64 | 2,97 | 3,77 | 4,23 | 4,42 | 3,50 | 4,69 | 3,40 | 4,15 | 3,72 | 3,09 | 2,84 | 3,00 | 2,91 | 3,15 | 2,91 |
| terrorizzare | NR | 2,29 | 2,92 | 2,52 | 2,96 | 2,00 | 2,89 | 2,09 | 2,84 | 2,38 | 3,21 | 1,74 | 2,33 | 2,60 | 2,52 | 4,10 | 2,69 | 1,10 | 1,10 | 2,43 | 3,76 | 1,00 | 1,31 | 4,33 | 5,16 |
| terrorizzarsi | R | 3,43 | 3,76 | 3,16 | 3,51 | 3,73 | 4,04 | 3,21 | 3,90 | 3,00 | 3,64 | 3,46 | 4,24 | 4,46 | 3,97 | 4,43 | 3,65 | 4,50 | 4,48 | 2,83 | 3,07 | 2,00 | 2,61 | 3,54 | 3,36 |
| tirare | NR | 1,75 | 2,10 | 1,83 | 2,12 | 1,65 | 2,10 | 1,39 | 1,98 | 1,26 | 1,96 | 1,60 | 2,06 | 2,21 | 2,40 | 2,54 | 2,44 | 1,82 | 2,40 | 1,87 | 1,94 | 2,17 | 1,90 | 1,55 | 2,02 |
| toccare | NR | 0,75 | 1,78 | 0,20 | 0,52 | 1,24 | 2,30 | 0,60 | 1,19 | 0,25 | 0,58 | 0,83 | 1,43 | 0,73 | 1,78 | 0,29 | 0,61 | 1,25 | 2,49 | 1,11 | 2,66 | 0,00 | 0,00 | 2,33 | 3,57 |
| torturare | NR | 4,46 | 4,33 | 5,00 | 4,40 | 3,85 | 4,23 | 4,31 | 4,41 | 5,17 | 4,66 | 3,33 | 4,00 | 3,65 | 4,03 | 4,64 | 4,30 | 2,44 | 3,54 | 5,72 | 4,42 | 5,00 | 4,27 | 6,44 | 4,69 |
| tradire | NR | 5,28 | 4,17 | 5,26 | 4,16 | 5,31 | 4,21 | 4,73 | 4,25 | 4,43 | 4,26 | 5,08 | 4,30 | 6,38 | 3,87 | 6,93 | 3,36 | 5,75 | 4,45 | 5,29 | 4,22 | 5,27 | 4,52 | 5,31 | 4,13 |
| vendicarsi | R | 3,59 | 3,98 | 4,44 | 4,43 | 2,73 | 3,32 | 3,68 | 3,81 | 4,32 | 3,97 | 3,05 | 3,63 | 2,29 | 3,39 | 2,92 | 4,27 | 1,67 | 2,23 | 4,95 | 4,63 | 6,50 | 5,04 | 3,40 | 3,81 |
| vergognarsi | R | 3,49 | 3,67 | 3,50 | 3,41 | 3,49 | 3,93 | 3,63 | 3,88 | 3,74 | 3,69 | 3,55 | 4,13 | 3,09 | 3,07 | 3,27 | 2,53 | 2,92 | 3,60 | 3,68 | 3,99 | 3,30 | 3,97 | 4,11 | 4,20 |
| versare | NR | 1,71 | 2,29 | 1,46 | 2,02 | 2,03 | 2,60 | 1,55 | 2,64 | 1,26 | 2,28 | 2,00 | 3,14 | 2,00 | 2,00 | 1,92 | 2,10 | 2,08 | 1,98 | 1,64 | 2,01 | 1,33 | 1,37 | 2,00 | 2,62 |
| vestirsi | R | 0,86 | 1,56 | 0,66 | 1,10 | 1,12 | 2,00 | 0,67 | 1,34 | 0,65 | 1,23 | 0,69 | 1,55 | 0,96 | 1,55 | 0,50 | 0,52 | 1,45 | 2,11 | 1,05 | 1,89 | 0,83 | 1,34 | 1,30 | 2,45 |

**Supplementary Table 3** - showing, for each verb, the means and the standard deviations (SDs) for the index of imageability. Means and SDs are reported for each age range (8-11 ALL, 12-15 ALL, 16-19 ALL), for males and females within each age range(8-11 females, 8-11 males; 12-15 females, 12-15 males; 16-19 females, 16-19 males), for males and females within the whole sample (females ALL, males ALL) and for the whole sample (ALL).
