# Supplementary material for: Age-Dependent Changes of Thinking about Verbs
Source: Front Behav Neurosci. 2017 Mar 14;11:40. doi: 10.3389/fnbeh.2017.00040 (PMC5348498; doi:10.3389/fnbeh.2017.00040)
Supplement: Supplementary file 5 [file Table5.DOCX]

**Supplementary Table 5 - Valence**

| **Arousal** | | **age range -> 8-19 (0VERALL SAMPLE)** | | | | | | **age range -> 8-11** | | | | | | **age range -> 12-15** | | | | | | **age range -> 16-19** | | | | | |
| --- | --- | --- | --- | --- | --- | --- | --- | --- | --- | --- | --- | --- | --- | --- | --- | --- | --- | --- | --- | --- | --- | --- | --- | --- | --- |
|  | R -NR | ALL | | F | | M | | ALL | | F | | M | | ALL | | F | | M | | ALL | | F | | M | |
|  |  | mean | sd | mean | sd | mean | sd | mean | sd | mean | sd | mean | sd | mean | sd | mean | sd | mean | sd | mean | sd | mean | sd | mean | sd |
| abbottonarsi | NR | 7,61 | 1,76 | 7,62 | 1,84 | 7,60 | 1,69 | 7,77 | 1,68 | 7,71 | 1,86 | 7,83 | 1,54 | 7,72 | 1,72 | 7,50 | 1,93 | 7,92 | 1,55 | 7,16 | 1,95 | 7,60 | 1,90 | 6,67 | 2,00 |
| afferrare | NR | 6,18 | 2,23 | 6,74 | 2,16 | 5,73 | 2,20 | 5,65 | 2,25 | 5,45 | 2,16 | 5,74 | 2,34 | 6,48 | 2,05 | 7,29 | 1,90 | 5,62 | 1,89 | 6,68 | 2,33 | 7,40 | 2,07 | 5,89 | 2,47 |
| affettare | NR | 6,11 | 2,46 | 6,08 | 2,42 | 6,14 | 2,53 | 5,81 | 2,62 | 5,52 | 2,69 | 6,16 | 2,54 | 6,40 | 2,54 | 7,10 | 2,02 | 5,70 | 2,91 | 6,57 | 1,79 | 6,43 | 1,51 | 6,71 | 2,14 |
| aggrapparsi | R | 4,44 | 2,45 | 4,56 | 2,46 | 4,27 | 2,46 | 4,67 | 2,55 | 4,78 | 2,43 | 4,50 | 2,78 | 3,54 | 2,45 | 4,31 | 2,87 | 2,64 | 1,50 | 5,00 | 2,07 | 4,42 | 2,19 | 5,70 | 1,77 |
| allacciarsi | R | 6,95 | 1,99 | 7,09 | 1,99 | 6,77 | 2,00 | 7,14 | 2,11 | 7,50 | 1,74 | 6,60 | 2,53 | 6,80 | 1,87 | 7,08 | 2,18 | 6,50 | 1,51 | 6,80 | 1,96 | 6,33 | 2,15 | 7,50 | 1,51 |
| amare | NR | 4,70 | 3,02 | 4,87 | 2,96 | 4,47 | 3,11 | 5,05 | 2,90 | 5,00 | 2,71 | 5,13 | 3,25 | 4,36 | 3,15 | 4,85 | 3,31 | 3,83 | 3,01 | 4,45 | 3,15 | 4,64 | 3,29 | 4,22 | 3,15 |
| amarsi | R | 5,34 | 2,91 | 5,46 | 2,67 | 5,22 | 3,16 | 4,71 | 2,80 | 5,35 | 2,62 | 4,14 | 2,90 | 6,00 | 2,61 | 5,42 | 2,31 | 6,78 | 2,91 | 6,00 | 3,28 | 5,78 | 3,46 | 6,20 | 3,29 |
| ammalarsi | R | 3,52 | 2,38 | 3,25 | 2,34 | 3,80 | 2,40 | 3,06 | 2,41 | 2,85 | 2,40 | 3,27 | 2,46 | 3,93 | 2,27 | 3,86 | 2,18 | 4,00 | 2,45 | 4,13 | 2,28 | 3,45 | 2,42 | 4,75 | 2,05 |
| annodare | NR | 6,57 | 2,04 | 6,47 | 2,10 | 6,67 | 1,99 | 6,47 | 2,20 | 6,42 | 2,35 | 6,52 | 2,08 | 6,93 | 1,88 | 7,14 | 1,99 | 6,69 | 1,80 | 6,38 | 1,86 | 5,73 | 1,35 | 6,92 | 2,10 |
| annoiarsi | R | 6,02 | 2,65 | 5,37 | 2,68 | 6,58 | 2,53 | 5,55 | 3,01 | 4,50 | 3,07 | 6,41 | 2,74 | 6,75 | 2,23 | 6,82 | 2,09 | 6,69 | 2,43 | 6,11 | 2,18 | 5,33 | 1,73 | 6,80 | 2,39 |
| apparecchiare | NR | 6,53 | 2,14 | 6,81 | 1,90 | 6,23 | 2,36 | 6,41 | 2,53 | 6,73 | 1,98 | 6,12 | 2,96 | 6,83 | 2,04 | 7,15 | 2,08 | 6,45 | 2,02 | 6,40 | 1,71 | 6,57 | 1,74 | 6,18 | 1,72 |
| appendere | NR | 7,10 | 1,99 | 7,14 | 1,80 | 7,05 | 2,18 | 6,90 | 2,16 | 7,20 | 1,94 | 6,58 | 2,39 | 7,29 | 1,90 | 6,75 | 1,91 | 7,83 | 1,80 | 7,25 | 1,77 | 7,50 | 1,43 | 7,00 | 2,11 |
| applaudire | NR | 6,46 | 2,48 | 6,24 | 2,45 | 6,65 | 2,51 | 6,00 | 2,63 | 6,00 | 2,69 | 6,00 | 2,65 | 7,08 | 2,41 | 6,83 | 2,48 | 7,31 | 2,43 | 6,56 | 2,12 | 5,89 | 2,03 | 7,22 | 2,11 |
| arrabbiarsi | R | 1,88 | 1,69 | 2,09 | 1,52 | 1,64 | 1,84 | 1,84 | 1,82 | 2,20 | 1,66 | 1,53 | 1,94 | 1,88 | 1,54 | 2,38 | 1,89 | 1,33 | 0,78 | 1,92 | 1,73 | 1,73 | 0,96 | 2,20 | 2,53 |
| avvitare | NR | 6,69 | 2,29 | 6,33 | 2,22 | 6,98 | 2,33 | 6,00 | 2,65 | 5,46 | 2,60 | 6,35 | 2,68 | 7,17 | 1,95 | 6,67 | 1,87 | 7,67 | 1,97 | 7,35 | 1,62 | 7,25 | 1,67 | 7,44 | 1,67 |
| avvolgersi | R | 5,76 | 2,00 | 5,86 | 1,78 | 5,64 | 2,26 | 5,55 | 2,39 | 5,53 | 1,92 | 5,56 | 2,83 | 5,50 | 1,47 | 5,46 | 1,20 | 5,55 | 1,81 | 6,29 | 1,88 | 6,53 | 1,96 | 5,89 | 1,76 |
| bussare | NR | 6,71 | 2,21 | 6,43 | 2,15 | 6,98 | 2,27 | 6,68 | 2,42 | 6,50 | 2,38 | 6,85 | 2,50 | 6,75 | 2,31 | 6,33 | 2,46 | 7,17 | 2,17 | 6,70 | 1,75 | 6,40 | 1,35 | 7,00 | 2,11 |
| calmare | NR | 6,57 | 2,31 | 6,71 | 2,28 | 6,46 | 2,35 | 6,21 | 2,38 | 6,20 | 2,21 | 6,22 | 2,54 | 6,52 | 2,46 | 6,46 | 2,60 | 6,57 | 2,41 | 7,37 | 1,80 | 7,80 | 1,69 | 6,89 | 1,90 |
| calmarsi | R | 6,27 | 2,62 | 6,10 | 2,46 | 6,45 | 2,80 | 6,73 | 2,47 | 6,15 | 2,60 | 7,32 | 2,21 | 5,05 | 2,66 | 5,77 | 2,39 | 4,00 | 2,83 | 6,42 | 2,65 | 6,36 | 2,38 | 6,46 | 2,96 |
| cancellare | NR | 7,24 | 2,05 | 7,33 | 2,10 | 7,14 | 2,01 | 7,28 | 2,20 | 7,47 | 2,27 | 7,04 | 2,14 | 7,12 | 1,93 | 7,14 | 1,83 | 7,08 | 2,11 | 7,29 | 1,90 | 7,18 | 2,09 | 7,38 | 1,80 |
| confessare | NR | 4,05 | 2,66 | 4,26 | 2,68 | 3,79 | 2,65 | 4,89 | 2,96 | 4,89 | 2,95 | 4,88 | 3,06 | 3,00 | 2,45 | 3,46 | 2,60 | 2,50 | 2,28 | 3,92 | 2,04 | 4,20 | 2,37 | 3,50 | 1,43 |
| confondersi | R | 4,16 | 2,25 | 4,18 | 2,17 | 4,14 | 2,35 | 3,95 | 2,22 | 4,58 | 2,29 | 3,35 | 2,03 | 4,60 | 1,98 | 4,25 | 1,96 | 4,92 | 2,02 | 4,00 | 2,65 | 3,22 | 2,11 | 4,70 | 2,98 |
| controllare | NR | 5,36 | 2,11 | 5,08 | 2,17 | 5,60 | 2,05 | 5,33 | 2,30 | 5,13 | 1,75 | 5,46 | 2,64 | 5,04 | 1,89 | 4,62 | 2,50 | 5,46 | 0,88 | 5,89 | 1,97 | 5,67 | 2,45 | 6,10 | 1,52 |
| controllarsi | R | 5,41 | 2,45 | 5,43 | 2,53 | 5,39 | 2,40 | 5,97 | 2,28 | 5,82 | 2,20 | 6,19 | 2,46 | 5,52 | 2,38 | 5,90 | 2,69 | 5,18 | 2,14 | 4,16 | 2,52 | 3,75 | 2,82 | 4,45 | 2,38 |
| credere | NR | 6,70 | 2,18 | 6,85 | 2,07 | 6,57 | 2,30 | 6,54 | 2,42 | 6,44 | 2,36 | 6,61 | 2,52 | 6,75 | 2,07 | 6,50 | 1,93 | 7,00 | 2,26 | 7,00 | 1,81 | 8,11 | 1,05 | 5,89 | 1,76 |
| decidere | NR | 4,77 | 2,69 | 4,61 | 2,66 | 4,93 | 2,75 | 5,16 | 3,04 | 4,71 | 2,85 | 5,70 | 3,25 | 3,58 | 2,41 | 3,67 | 2,40 | 3,50 | 2,55 | 5,00 | 1,91 | 5,18 | 2,44 | 4,83 | 1,34 |
| deludere | NR | 3,87 | 2,26 | 3,82 | 2,35 | 3,92 | 2,18 | 3,74 | 2,49 | 3,67 | 2,58 | 3,82 | 2,46 | 4,00 | 2,43 | 4,08 | 2,68 | 3,91 | 2,26 | 3,95 | 1,63 | 3,83 | 1,64 | 4,11 | 1,69 |
| deprimersi | R | 4,37 | 2,67 | 4,17 | 2,53 | 4,60 | 2,86 | 4,38 | 3,02 | 4,71 | 3,10 | 4,00 | 3,02 | 3,85 | 2,37 | 4,00 | 2,41 | 3,67 | 2,45 | 4,89 | 2,49 | 3,60 | 1,71 | 6,33 | 2,50 |
| desiderare | NR | 5,87 | 2,73 | 5,92 | 2,62 | 5,82 | 2,86 | 5,90 | 2,94 | 5,93 | 2,89 | 5,88 | 3,05 | 5,81 | 2,30 | 6,29 | 1,86 | 5,31 | 2,69 | 5,88 | 2,80 | 5,45 | 2,88 | 6,23 | 2,80 |
| dimenticare | NR | 4,74 | 2,39 | 4,89 | 2,46 | 4,58 | 2,32 | 5,14 | 2,64 | 6,18 | 2,56 | 4,21 | 2,42 | 4,38 | 2,57 | 3,85 | 2,58 | 5,00 | 2,53 | 4,52 | 1,73 | 4,33 | 1,59 | 4,80 | 1,99 |
| dipingere | NR | 8,18 | 1,49 | 8,40 | 1,28 | 7,96 | 1,67 | 8,13 | 1,54 | 8,33 | 1,32 | 7,88 | 1,76 | 8,04 | 1,60 | 8,29 | 1,49 | 7,77 | 1,74 | 8,50 | 1,22 | 8,78 | 0,67 | 8,31 | 1,49 |
| disegnare | NR | 7,62 | 2,19 | 7,91 | 1,84 | 7,34 | 2,46 | 7,28 | 2,50 | 7,43 | 2,38 | 7,17 | 2,66 | 7,82 | 2,20 | 8,09 | 1,64 | 7,55 | 2,70 | 7,95 | 1,54 | 8,40 | 0,97 | 7,44 | 1,94 |
| dispiacersi | R | 4,15 | 2,39 | 4,24 | 2,27 | 4,03 | 2,56 | 4,26 | 2,34 | 4,54 | 2,38 | 3,89 | 2,31 | 4,88 | 2,26 | 4,50 | 1,84 | 5,43 | 2,82 | 3,30 | 2,45 | 3,36 | 2,34 | 3,22 | 2,73 |
| fallire | NR | 2,90 | 2,02 | 3,09 | 2,14 | 2,69 | 1,87 | 3,24 | 2,24 | 3,50 | 2,13 | 3,00 | 2,38 | 2,75 | 1,80 | 3,15 | 2,08 | 2,27 | 1,35 | 2,60 | 1,89 | 2,60 | 2,26 | 2,60 | 1,26 |
| fingere | NR | 4,46 | 2,53 | 3,46 | 2,43 | 5,24 | 2,35 | 4,74 | 2,76 | 3,17 | 2,62 | 5,59 | 2,48 | 3,92 | 2,35 | 3,15 | 2,38 | 4,69 | 2,14 | 4,70 | 2,36 | 4,20 | 2,35 | 5,20 | 2,39 |
| fotografarsi | R | 6,29 | 2,50 | 6,47 | 2,35 | 6,08 | 2,68 | 6,19 | 2,67 | 6,40 | 2,42 | 5,95 | 2,97 | 7,05 | 2,26 | 7,60 | 1,90 | 6,50 | 2,55 | 5,46 | 2,03 | 5,25 | 2,25 | 5,80 | 1,79 |
| gettare | NR | 5,83 | 2,10 | 5,54 | 2,08 | 6,07 | 2,11 | 5,43 | 2,10 | 5,00 | 2,04 | 5,70 | 2,14 | 5,96 | 2,09 | 5,92 | 2,10 | 6,00 | 2,16 | 6,40 | 2,06 | 5,80 | 2,15 | 7,00 | 1,89 |
| graffiarsi | R | 4,16 | 2,21 | 3,84 | 2,01 | 4,48 | 2,37 | 3,94 | 2,29 | 3,32 | 1,96 | 4,62 | 2,45 | 4,22 | 1,78 | 4,00 | 1,81 | 4,45 | 1,81 | 4,58 | 2,41 | 5,00 | 2,00 | 4,23 | 2,74 |
| grattarsi | R | 5,64 | 2,39 | 5,55 | 2,31 | 5,74 | 2,52 | 5,42 | 2,83 | 5,44 | 2,61 | 5,40 | 3,14 | 6,09 | 2,37 | 5,73 | 2,57 | 6,45 | 2,21 | 5,50 | 1,77 | 5,53 | 1,88 | 5,44 | 1,67 |
| grattugiare | NR | 6,75 | 2,21 | 6,68 | 2,07 | 6,82 | 2,38 | 6,60 | 2,35 | 6,54 | 2,17 | 6,67 | 2,59 | 7,25 | 1,97 | 7,30 | 2,06 | 7,20 | 1,99 | 6,56 | 2,12 | 6,40 | 1,90 | 6,75 | 2,49 |
| guarire | NR | 5,75 | 2,39 | 5,55 | 2,38 | 6,00 | 2,40 | 5,63 | 3,02 | 5,63 | 3,16 | 5,63 | 2,99 | 5,67 | 1,93 | 5,15 | 1,91 | 6,27 | 1,85 | 6,00 | 1,87 | 5,80 | 1,82 | 6,30 | 2,00 |
| impaurirsi | R | 2,42 | 2,11 | 2,31 | 2,07 | 2,54 | 2,17 | 2,54 | 2,28 | 2,56 | 2,19 | 2,52 | 2,42 | 2,62 | 2,28 | 2,23 | 2,24 | 3,00 | 2,35 | 1,90 | 1,34 | 1,67 | 1,41 | 2,08 | 1,31 |
| impazzire | NR | 2,17 | 2,14 | 2,35 | 2,10 | 1,94 | 2,21 | 1,92 | 1,93 | 2,41 | 2,32 | 1,14 | 0,53 | 1,92 | 1,86 | 2,08 | 1,32 | 1,73 | 2,41 | 2,90 | 2,66 | 2,55 | 2,50 | 3,30 | 2,91 |
| impressionarsi | R | 3,70 | 2,19 | 3,51 | 2,09 | 3,88 | 2,29 | 3,56 | 2,16 | 3,42 | 1,84 | 3,71 | 2,52 | 4,32 | 2,21 | 4,50 | 2,43 | 4,15 | 2,08 | 3,11 | 2,11 | 2,25 | 1,49 | 3,80 | 2,35 |
| inchiodare | NR | 5,13 | 2,70 | 4,56 | 2,47 | 5,75 | 2,84 | 5,22 | 3,00 | 4,59 | 2,79 | 5,95 | 3,14 | 5,37 | 2,29 | 4,60 | 2,46 | 6,22 | 1,86 | 4,60 | 2,41 | 4,43 | 1,51 | 4,75 | 3,11 |
| incollarsi | R | 4,75 | 2,37 | 4,43 | 2,22 | 5,09 | 2,52 | 4,73 | 2,68 | 4,18 | 2,58 | 5,39 | 2,73 | 5,05 | 2,15 | 4,89 | 1,96 | 5,20 | 2,39 | 4,33 | 1,56 | 4,67 | 0,82 | 4,00 | 2,10 |
| indicarsi | R | 6,28 | 2,01 | 6,02 | 2,03 | 6,52 | 1,99 | 6,36 | 2,13 | 6,05 | 2,24 | 6,65 | 2,04 | 6,41 | 1,99 | 6,23 | 1,92 | 6,57 | 2,10 | 6,00 | 1,87 | 5,73 | 1,85 | 6,23 | 1,92 |
| infilare | NR | 6,84 | 2,18 | 7,21 | 2,09 | 6,46 | 2,23 | 7,24 | 2,19 | 7,75 | 2,01 | 6,70 | 2,27 | 5,88 | 2,27 | 6,29 | 2,02 | 5,30 | 2,58 | 6,91 | 1,83 | 7,00 | 2,11 | 6,85 | 1,68 |
| ingelosirsi | R | 2,86 | 2,14 | 2,75 | 1,93 | 3,00 | 2,42 | 3,24 | 2,36 | 2,95 | 1,96 | 3,69 | 2,93 | 2,50 | 1,90 | 3,08 | 2,11 | 1,80 | 1,40 | 2,60 | 1,98 | 2,00 | 1,61 | 3,33 | 2,24 |
| insultare | NR | 2,68 | 1,97 | 2,69 | 1,94 | 2,67 | 2,03 | 2,88 | 2,11 | 3,07 | 2,40 | 2,71 | 1,86 | 2,48 | 1,73 | 2,33 | 1,78 | 2,64 | 1,75 | 2,62 | 2,06 | 2,60 | 1,59 | 2,64 | 2,66 |
| intrecciarsi | R | 5,10 | 2,04 | 5,34 | 1,82 | 4,82 | 2,27 | 4,66 | 2,09 | 5,00 | 1,63 | 4,38 | 2,42 | 5,29 | 2,12 | 5,55 | 2,02 | 5,00 | 2,31 | 5,52 | 1,86 | 5,50 | 1,91 | 5,57 | 1,90 |
| intristirsi | R | 4,29 | 2,32 | 4,63 | 2,21 | 3,89 | 2,41 | 4,93 | 2,65 | 5,50 | 2,25 | 4,29 | 3,00 | 3,67 | 2,01 | 4,17 | 2,17 | 3,17 | 1,80 | 4,12 | 2,05 | 4,07 | 2,05 | 4,20 | 2,15 |
| invidiare | NR | 3,38 | 2,35 | 3,25 | 2,47 | 3,53 | 2,21 | 3,06 | 2,51 | 2,88 | 2,50 | 3,27 | 2,60 | 3,70 | 2,46 | 4,00 | 2,89 | 3,36 | 1,96 | 3,48 | 2,04 | 3,07 | 2,09 | 4,10 | 1,91 |
| irritarsi | NR | 2,54 | 1,75 | 2,39 | 1,69 | 2,77 | 1,85 | 3,00 | 1,83 | 2,79 | 1,96 | 3,40 | 1,58 | 2,33 | 1,49 | 2,62 | 1,39 | 2,00 | 1,61 | 2,14 | 1,85 | 1,50 | 1,24 | 3,00 | 2,24 |
| lanciare | NR | 5,34 | 2,40 | 5,48 | 2,64 | 5,22 | 2,20 | 4,78 | 2,34 | 4,94 | 2,54 | 4,67 | 2,24 | 5,89 | 2,26 | 6,14 | 2,44 | 5,62 | 2,10 | 5,74 | 2,60 | 5,40 | 3,10 | 6,11 | 2,03 |
| lavarsi | R | 7,59 | 2,04 | 7,68 | 1,59 | 7,50 | 2,41 | 7,86 | 1,93 | 8,00 | 1,38 | 7,73 | 2,35 | 7,67 | 2,01 | 7,50 | 1,93 | 7,83 | 2,17 | 6,89 | 2,26 | 7,22 | 1,56 | 6,60 | 2,80 |
| legare | NR | 5,29 | 2,31 | 5,30 | 2,14 | 5,28 | 2,51 | 5,64 | 2,40 | 5,56 | 2,36 | 5,72 | 2,52 | 5,43 | 2,48 | 5,67 | 2,31 | 5,18 | 2,75 | 4,70 | 1,96 | 4,75 | 1,73 | 4,64 | 2,34 |
| maledire | NR | 2,78 | 1,84 | 2,32 | 1,63 | 3,16 | 1,93 | 3,24 | 2,00 | 2,62 | 1,80 | 3,65 | 2,06 | 2,46 | 1,71 | 2,29 | 1,68 | 2,64 | 1,78 | 2,45 | 1,64 | 2,00 | 1,41 | 2,90 | 1,79 |
| martellare | NR | 4,57 | 2,41 | 4,25 | 2,28 | 4,97 | 2,53 | 4,68 | 2,37 | 4,45 | 2,42 | 5,00 | 2,35 | 3,92 | 2,38 | 3,38 | 1,89 | 4,55 | 2,81 | 5,14 | 2,43 | 4,91 | 2,34 | 5,40 | 2,63 |
| mentire | NR | 3,46 | 2,50 | 2,92 | 2,44 | 3,95 | 2,47 | 3,58 | 2,45 | 3,61 | 2,59 | 3,55 | 2,37 | 2,91 | 2,00 | 2,09 | 1,64 | 3,73 | 2,05 | 3,84 | 3,08 | 2,56 | 2,79 | 5,00 | 2,98 |
| meritare | NR | 5,57 | 2,49 | 5,90 | 2,43 | 5,23 | 2,55 | 5,15 | 2,82 | 5,76 | 2,79 | 4,47 | 2,76 | 6,62 | 2,31 | 6,90 | 2,02 | 6,36 | 2,62 | 5,30 | 1,63 | 5,20 | 1,75 | 5,40 | 1,58 |
| mescolare | NR | 7,27 | 2,06 | 7,20 | 1,83 | 7,35 | 2,29 | 7,13 | 2,20 | 7,30 | 1,75 | 6,95 | 2,63 | 7,50 | 1,98 | 7,17 | 1,99 | 7,83 | 1,99 | 7,28 | 1,93 | 7,00 | 2,00 | 7,56 | 1,94 |
| morire | NR | 2,59 | 2,52 | 2,30 | 2,35 | 2,90 | 2,69 | 3,00 | 2,93 | 2,56 | 2,58 | 3,55 | 3,30 | 1,70 | 1,45 | 1,50 | 1,27 | 1,90 | 1,66 | 2,57 | 2,25 | 2,45 | 2,54 | 2,67 | 2,06 |
| obbedire | NR | 5,60 | 2,19 | 5,70 | 2,06 | 5,47 | 2,36 | 6,40 | 2,19 | 6,29 | 2,37 | 6,57 | 1,95 | 5,13 | 2,09 | 5,33 | 1,67 | 4,92 | 2,50 | 4,81 | 1,94 | 5,00 | 1,61 | 4,60 | 2,32 |
| odiare | NR | 3,20 | 2,25 | 2,63 | 1,91 | 3,66 | 2,41 | 3,37 | 2,24 | 2,73 | 1,67 | 3,78 | 2,49 | 3,18 | 2,37 | 2,86 | 2,41 | 3,50 | 2,38 | 2,89 | 2,16 | 2,11 | 1,45 | 3,60 | 2,50 |
| odiarsi | R | 2,37 | 1,94 | 2,34 | 1,92 | 2,41 | 1,99 | 2,26 | 2,10 | 2,31 | 1,70 | 2,22 | 2,39 | 2,04 | 1,46 | 2,23 | 1,74 | 1,80 | 1,03 | 2,83 | 2,12 | 2,47 | 2,33 | 3,44 | 1,67 |
| peccare | NR | 3,30 | 2,46 | 3,30 | 2,40 | 3,29 | 2,55 | 3,34 | 2,40 | 3,30 | 2,27 | 3,38 | 2,58 | 3,00 | 2,13 | 2,69 | 1,97 | 3,36 | 2,34 | 3,52 | 2,92 | 4,10 | 3,07 | 3,08 | 2,84 |
| penare | NR | 3,58 | 2,37 | 2,97 | 2,07 | 4,15 | 2,52 | 3,48 | 2,57 | 2,80 | 1,86 | 4,13 | 3,01 | 4,28 | 2,52 | 4,00 | 2,83 | 4,50 | 2,37 | 3,00 | 1,70 | 2,33 | 1,41 | 3,75 | 1,75 |
| pennellarsi | R | 5,59 | 2,42 | 6,02 | 2,26 | 5,15 | 2,52 | 5,04 | 2,68 | 5,60 | 2,55 | 4,46 | 2,75 | 6,38 | 2,16 | 7,36 | 1,75 | 5,30 | 2,11 | 6,05 | 1,70 | 5,60 | 1,35 | 6,42 | 1,93 |
| perdere | NR | 3,53 | 2,31 | 3,40 | 2,25 | 3,67 | 2,39 | 3,36 | 2,33 | 3,59 | 2,35 | 3,13 | 2,36 | 3,24 | 2,11 | 2,85 | 1,72 | 3,67 | 2,46 | 4,09 | 2,48 | 3,75 | 2,67 | 4,45 | 2,34 |
| perdonare | NR | 5,64 | 2,46 | 5,49 | 2,45 | 5,79 | 2,48 | 5,66 | 2,66 | 5,57 | 2,62 | 5,76 | 2,80 | 5,64 | 2,44 | 5,55 | 2,42 | 5,73 | 2,57 | 5,60 | 2,16 | 5,22 | 2,33 | 5,91 | 2,07 |
| perdonarsi | R | 5,56 | 2,33 | 5,39 | 2,30 | 5,72 | 2,37 | 5,54 | 2,30 | 6,26 | 2,33 | 4,78 | 2,07 | 5,38 | 2,52 | 4,42 | 2,11 | 6,33 | 2,61 | 5,88 | 2,19 | 4,71 | 1,80 | 6,78 | 2,11 |
| perseguitare | NR | 3,41 | 2,32 | 3,29 | 2,06 | 3,56 | 2,62 | 3,96 | 2,72 | 4,15 | 2,51 | 3,79 | 2,99 | 2,84 | 1,91 | 3,00 | 1,83 | 2,67 | 2,06 | 3,39 | 2,15 | 2,80 | 1,70 | 4,50 | 2,56 |
| pettinarsi | R | 6,87 | 2,32 | 7,00 | 2,19 | 6,73 | 2,47 | 6,75 | 2,47 | 7,00 | 2,38 | 6,42 | 2,61 | 8,00 | 1,72 | 7,55 | 2,02 | 8,45 | 1,29 | 5,90 | 2,10 | 6,40 | 1,90 | 5,40 | 2,27 |
| piacere | NR | 5,78 | 2,46 | 5,77 | 2,87 | 5,78 | 2,12 | 6,11 | 2,42 | 6,07 | 2,79 | 6,13 | 2,24 | 5,27 | 2,52 | 5,77 | 3,11 | 4,77 | 1,74 | 5,82 | 2,46 | 5,25 | 2,92 | 6,33 | 2,00 |
| piacersi | R | 5,06 | 2,45 | 5,54 | 2,62 | 4,60 | 2,21 | 4,73 | 2,63 | 5,25 | 2,79 | 4,24 | 2,43 | 5,57 | 2,19 | 6,00 | 2,57 | 5,17 | 1,80 | 5,15 | 2,35 | 5,60 | 2,50 | 4,70 | 2,21 |
| piangere | NR | 2,75 | 1,84 | 2,70 | 1,59 | 2,82 | 2,13 | 2,69 | 2,05 | 2,59 | 1,62 | 2,80 | 2,51 | 2,68 | 1,60 | 2,85 | 1,52 | 2,50 | 1,73 | 2,89 | 1,85 | 2,69 | 1,70 | 3,18 | 2,09 |
| piegare | NR | 6,45 | 2,40 | 6,38 | 2,49 | 6,53 | 2,33 | 6,41 | 2,70 | 6,60 | 2,77 | 6,16 | 2,65 | 7,05 | 2,31 | 6,20 | 2,74 | 7,82 | 1,60 | 5,90 | 1,65 | 6,00 | 1,41 | 5,80 | 1,93 |
| pinzare | NR | 6,71 | 2,06 | 6,81 | 2,02 | 6,64 | 2,11 | 6,35 | 2,21 | 5,89 | 2,03 | 6,55 | 2,30 | 6,77 | 1,84 | 7,00 | 2,00 | 6,54 | 1,71 | 7,21 | 2,10 | 7,44 | 1,94 | 7,00 | 2,31 |
| pizzicarsi | R | 4,38 | 2,25 | 4,50 | 2,00 | 4,24 | 2,54 | 4,24 | 2,37 | 4,33 | 2,22 | 4,13 | 2,63 | 5,40 | 1,76 | 5,30 | 1,64 | 5,50 | 1,96 | 3,29 | 2,05 | 3,86 | 1,57 | 2,71 | 2,43 |
| posare | NR | 6,18 | 2,44 | 5,90 | 2,46 | 6,50 | 2,42 | 7,31 | 2,35 | 7,46 | 2,03 | 7,19 | 2,64 | 5,00 | 2,45 | 4,67 | 2,67 | 5,44 | 2,19 | 5,83 | 1,97 | 5,50 | 1,95 | 6,33 | 2,00 |
| premere | NR | 5,93 | 1,99 | 5,91 | 1,90 | 5,94 | 2,14 | 6,16 | 2,10 | 6,00 | 2,11 | 6,43 | 2,14 | 5,75 | 1,96 | 6,08 | 1,68 | 5,42 | 2,23 | 5,71 | 1,87 | 5,55 | 1,75 | 5,90 | 2,08 |
| prendere | NR | 6,00 | 2,11 | 6,28 | 2,03 | 5,66 | 2,18 | 6,27 | 2,32 | 6,86 | 2,06 | 5,50 | 2,48 | 5,84 | 1,52 | 5,77 | 1,30 | 5,92 | 1,78 | 5,73 | 2,35 | 5,83 | 2,48 | 5,60 | 2,32 |
| preoccuparsi | R | 2,58 | 2,01 | 2,59 | 2,16 | 2,58 | 1,89 | 2,95 | 2,36 | 3,11 | 2,35 | 2,80 | 2,42 | 2,40 | 1,91 | 2,33 | 2,46 | 2,46 | 1,33 | 2,10 | 1,17 | 1,90 | 0,99 | 2,30 | 1,34 |
| punire | NR | 3,09 | 2,08 | 2,94 | 1,84 | 3,24 | 2,32 | 2,68 | 1,76 | 2,50 | 1,61 | 2,88 | 1,93 | 3,62 | 2,38 | 3,43 | 2,24 | 3,83 | 2,62 | 3,50 | 2,30 | 3,60 | 1,65 | 3,42 | 2,81 |
| punirsi | R | 3,55 | 2,22 | 2,97 | 2,11 | 4,00 | 2,22 | 3,78 | 2,31 | 2,53 | 1,73 | 4,52 | 2,33 | 3,39 | 2,08 | 3,57 | 2,41 | 3,21 | 1,76 | 3,30 | 2,27 | 2,80 | 2,20 | 3,80 | 2,35 |
| raccogliere | NR | 6,98 | 1,99 | 7,42 | 2,10 | 6,63 | 1,84 | 6,73 | 2,17 | 7,40 | 2,32 | 6,32 | 2,01 | 7,26 | 1,75 | 7,62 | 1,89 | 6,93 | 1,59 | 7,11 | 1,94 | 7,20 | 2,20 | 7,00 | 1,73 |
| rallegrarsi | R | 6,49 | 2,64 | 6,49 | 2,77 | 6,49 | 2,56 | 6,37 | 2,81 | 6,36 | 2,53 | 6,38 | 3,04 | 7,11 | 2,58 | 7,43 | 2,95 | 6,77 | 2,17 | 5,78 | 2,29 | 5,22 | 2,54 | 6,33 | 2,00 |
| rastrellare | NR | 6,09 | 2,30 | 6,57 | 1,96 | 5,53 | 2,55 | 6,62 | 2,12 | 6,95 | 1,78 | 6,20 | 2,48 | 5,77 | 2,54 | 6,45 | 2,21 | 5,09 | 2,77 | 5,59 | 2,24 | 6,08 | 2,07 | 5,00 | 2,40 |
| rattristarsi | R | 4,44 | 2,55 | 3,98 | 2,26 | 4,95 | 2,78 | 4,27 | 2,55 | 4,33 | 2,74 | 4,20 | 2,40 | 4,79 | 2,13 | 4,08 | 1,56 | 5,50 | 2,43 | 4,30 | 3,06 | 3,20 | 1,99 | 5,40 | 3,63 |
| ridere | NR | 5,63 | 2,67 | 5,80 | 2,59 | 5,43 | 2,77 | 5,14 | 2,98 | 5,39 | 2,81 | 4,89 | 3,20 | 5,92 | 2,21 | 6,33 | 2,31 | 5,50 | 2,11 | 6,04 | 2,58 | 5,88 | 2,60 | 6,30 | 2,67 |
| rifiutare | NR | 5,52 | 2,26 | 5,63 | 2,01 | 5,41 | 2,49 | 5,63 | 2,19 | 5,64 | 1,78 | 5,62 | 2,65 | 5,42 | 2,81 | 5,88 | 3,18 | 5,09 | 2,63 | 5,36 | 1,92 | 5,40 | 1,58 | 5,33 | 2,23 |
| rilassarsi | R | 8,23 | 1,68 | 8,05 | 1,65 | 8,44 | 1,71 | 7,94 | 2,12 | 8,00 | 1,81 | 7,89 | 2,40 | 8,12 | 1,64 | 7,31 | 1,97 | 9,00 | 0,00 | 8,75 | 0,68 | 8,73 | 0,70 | 8,78 | 0,67 |
| rubare | NR | 2,60 | 2,22 | 2,15 | 1,56 | 3,09 | 2,70 | 2,76 | 2,34 | 2,23 | 1,50 | 3,35 | 2,95 | 2,73 | 2,16 | 1,92 | 1,75 | 3,89 | 2,26 | 2,10 | 2,00 | 2,22 | 1,56 | 2,00 | 2,34 |
| ruotare | NR | 5,51 | 2,31 | 5,83 | 2,21 | 5,10 | 2,40 | 5,14 | 2,42 | 5,70 | 2,40 | 4,21 | 2,22 | 6,30 | 2,20 | 6,27 | 1,68 | 6,33 | 2,83 | 5,40 | 2,03 | 5,57 | 2,51 | 5,25 | 1,67 |
| sanguinare | NR | 3,09 | 2,42 | 2,58 | 2,02 | 3,63 | 2,70 | 3,02 | 2,49 | 2,52 | 2,17 | 3,63 | 2,77 | 3,23 | 2,74 | 2,09 | 1,81 | 4,36 | 3,11 | 3,07 | 1,69 | 3,67 | 1,63 | 2,63 | 1,69 |
| sbagliare | NR | 3,81 | 2,33 | 3,36 | 1,92 | 4,35 | 2,67 | 4,08 | 2,35 | 3,40 | 1,76 | 4,94 | 2,74 | 3,25 | 2,21 | 2,92 | 2,02 | 3,64 | 2,46 | 4,00 | 2,43 | 3,82 | 2,14 | 4,20 | 2,82 |
| sbattere | NR | 3,95 | 2,55 | 3,90 | 2,38 | 4,03 | 2,78 | 4,53 | 2,76 | 4,26 | 2,63 | 4,93 | 2,99 | 3,12 | 2,24 | 3,38 | 2,22 | 2,83 | 2,33 | 3,91 | 2,35 | 3,75 | 2,09 | 4,09 | 2,70 |
| scarabocchiare | NR | 6,46 | 2,47 | 6,71 | 2,65 | 6,26 | 2,32 | 5,70 | 2,69 | 5,38 | 2,73 | 5,92 | 2,70 | 7,29 | 1,71 | 7,75 | 1,76 | 6,83 | 1,59 | 7,00 | 2,43 | 7,60 | 2,67 | 6,40 | 2,12 |
| schiacciare | NR | 5,47 | 2,24 | 5,74 | 2,35 | 5,26 | 2,16 | 5,10 | 2,46 | 4,80 | 2,40 | 5,28 | 2,53 | 6,14 | 2,10 | 7,00 | 2,32 | 5,29 | 1,49 | 5,29 | 1,85 | 5,40 | 1,58 | 5,18 | 2,14 |
| sconfiggere | NR | 4,21 | 2,33 | 3,92 | 2,20 | 4,51 | 2,44 | 4,04 | 2,48 | 3,65 | 2,38 | 4,48 | 2,57 | 4,33 | 2,29 | 4,43 | 2,41 | 4,23 | 2,24 | 4,42 | 2,10 | 3,91 | 1,38 | 4,85 | 2,54 |
| scoraggiarsi | R | 3,84 | 2,03 | 4,17 | 1,86 | 3,41 | 2,18 | 3,70 | 1,92 | 4,64 | 1,08 | 2,69 | 2,14 | 3,92 | 2,50 | 4,38 | 2,75 | 3,36 | 2,16 | 3,91 | 1,63 | 3,50 | 1,29 | 4,63 | 2,00 |
| scrivere | NR | 7,21 | 2,09 | 7,24 | 2,06 | 7,18 | 2,15 | 6,96 | 2,31 | 7,21 | 2,24 | 6,68 | 2,41 | 7,20 | 1,91 | 7,21 | 2,01 | 7,18 | 1,89 | 7,79 | 1,64 | 7,36 | 1,75 | 8,15 | 1,52 |
| scuotere | NR | 5,24 | 2,68 | 5,15 | 2,80 | 5,32 | 2,59 | 5,00 | 2,76 | 4,93 | 2,40 | 5,05 | 3,03 | 5,89 | 2,49 | 5,64 | 2,82 | 6,15 | 2,15 | 4,79 | 2,74 | 4,80 | 3,46 | 4,78 | 1,86 |
| scusare | NR | 5,42 | 2,54 | 5,19 | 2,47 | 5,61 | 2,60 | 4,94 | 2,90 | 3,73 | 2,52 | 5,81 | 2,89 | 5,65 | 2,31 | 6,00 | 2,00 | 5,36 | 2,59 | 5,95 | 2,06 | 6,40 | 1,90 | 5,55 | 2,21 |
| scusarsi | R | 4,37 | 2,36 | 4,10 | 2,41 | 4,63 | 2,31 | 4,85 | 2,28 | 4,79 | 1,99 | 4,90 | 2,57 | 4,00 | 2,38 | 3,92 | 2,75 | 4,08 | 2,07 | 3,84 | 2,43 | 3,00 | 2,49 | 4,78 | 2,11 |
| segare | NR | 4,94 | 2,28 | 4,55 | 2,21 | 5,28 | 2,31 | 4,29 | 2,42 | 3,93 | 2,09 | 4,55 | 2,65 | 5,43 | 2,12 | 5,00 | 2,22 | 5,86 | 1,99 | 5,37 | 2,09 | 4,80 | 2,39 | 6,00 | 1,58 |
| sfidare | NR | 3,07 | 1,85 | 3,15 | 2,07 | 3,00 | 1,64 | 2,80 | 1,95 | 3,13 | 2,19 | 2,45 | 1,63 | 3,79 | 1,90 | 3,56 | 2,40 | 4,00 | 1,41 | 3,00 | 1,37 | 2,75 | 1,28 | 3,20 | 1,48 |
| sganciare | NR | 5,41 | 2,08 | 5,41 | 1,79 | 5,41 | 2,41 | 5,13 | 2,29 | 5,94 | 1,85 | 4,14 | 2,44 | 5,43 | 1,90 | 5,15 | 1,52 | 5,80 | 2,35 | 5,81 | 1,97 | 4,91 | 1,92 | 6,80 | 1,55 |
| slegarsi | R | 5,41 | 2,45 | 5,53 | 2,41 | 5,30 | 2,52 | 5,29 | 2,60 | 5,86 | 2,33 | 4,70 | 2,79 | 5,42 | 2,06 | 5,08 | 2,19 | 5,75 | 1,96 | 5,73 | 2,74 | 5,29 | 3,15 | 6,13 | 2,47 |
| soffrire | NR | 2,93 | 2,49 | 3,26 | 2,67 | 2,53 | 2,21 | 3,20 | 2,75 | 3,26 | 2,77 | 3,11 | 2,81 | 2,71 | 2,00 | 3,50 | 2,24 | 1,67 | 1,00 | 2,64 | 2,44 | 3,00 | 3,10 | 2,27 | 1,62 |
| sognare | NR | 7,22 | 2,09 | 7,17 | 2,14 | 7,28 | 2,05 | 7,46 | 1,86 | 7,73 | 1,80 | 7,12 | 1,93 | 7,16 | 2,58 | 6,54 | 2,60 | 7,83 | 2,48 | 6,86 | 1,88 | 6,82 | 2,09 | 6,90 | 1,73 |
| sollevare | NR | 5,02 | 2,51 | 5,18 | 2,55 | 4,85 | 2,49 | 4,73 | 2,64 | 5,08 | 2,75 | 4,26 | 2,49 | 5,85 | 2,48 | 5,40 | 2,59 | 6,30 | 2,41 | 4,84 | 2,12 | 5,22 | 2,11 | 4,50 | 2,17 |
| sospettare | NR | 4,46 | 2,28 | 4,21 | 2,26 | 4,71 | 2,30 | 4,83 | 2,21 | 4,46 | 2,12 | 5,23 | 2,29 | 4,15 | 2,40 | 4,00 | 2,80 | 4,31 | 1,97 | 3,96 | 2,26 | 3,82 | 1,94 | 4,08 | 2,56 |
| spalmare | NR | 6,43 | 1,88 | 6,27 | 1,84 | 6,57 | 1,93 | 6,24 | 2,05 | 5,69 | 1,89 | 6,52 | 2,10 | 6,54 | 1,63 | 6,43 | 1,65 | 6,67 | 1,67 | 6,68 | 1,92 | 6,80 | 1,99 | 6,56 | 1,94 |
| spaventare | NR | 3,61 | 2,39 | 3,83 | 2,53 | 3,37 | 2,24 | 3,67 | 2,29 | 3,40 | 2,29 | 3,89 | 2,32 | 3,35 | 2,81 | 4,50 | 3,21 | 2,09 | 1,64 | 3,79 | 2,17 | 3,73 | 2,19 | 3,89 | 2,26 |
| spaventarsi | R | 2,55 | 2,28 | 2,51 | 2,25 | 2,59 | 2,34 | 3,18 | 2,80 | 2,79 | 2,74 | 3,58 | 2,87 | 2,41 | 1,71 | 2,55 | 1,86 | 2,27 | 1,62 | 1,50 | 1,10 | 1,89 | 1,45 | 1,18 | 0,60 |
| spazzolarsi | R | 6,63 | 2,11 | 6,67 | 2,16 | 6,58 | 2,08 | 6,43 | 2,28 | 6,76 | 2,17 | 6,00 | 2,42 | 6,88 | 2,01 | 6,69 | 2,29 | 7,09 | 1,70 | 6,70 | 1,98 | 6,45 | 2,21 | 7,00 | 1,73 |
| sperare | NR | 5,27 | 2,63 | 5,02 | 2,67 | 5,56 | 2,58 | 5,00 | 2,79 | 4,82 | 3,02 | 5,25 | 2,52 | 5,63 | 2,67 | 5,42 | 2,54 | 5,83 | 2,89 | 5,35 | 2,37 | 5,00 | 2,26 | 5,73 | 2,53 |
| spezzare | NR | 4,80 | 2,44 | 4,66 | 2,44 | 4,91 | 2,46 | 4,56 | 2,56 | 4,47 | 2,33 | 4,63 | 2,75 | 4,78 | 1,93 | 4,79 | 2,01 | 4,77 | 1,92 | 5,32 | 2,85 | 4,78 | 3,38 | 5,80 | 2,35 |
| spingere | NR | 4,51 | 2,12 | 4,44 | 2,05 | 4,59 | 2,21 | 3,94 | 2,15 | 3,86 | 1,70 | 4,00 | 2,50 | 4,25 | 2,19 | 4,08 | 2,40 | 4,45 | 2,02 | 5,46 | 1,70 | 5,25 | 1,88 | 5,80 | 1,40 |
| spremere | NR | 6,01 | 2,17 | 6,02 | 2,03 | 6,00 | 2,35 | 6,43 | 2,30 | 7,00 | 1,87 | 5,89 | 2,59 | 5,72 | 2,51 | 5,62 | 2,63 | 5,83 | 2,48 | 5,72 | 1,51 | 5,27 | 1,03 | 6,40 | 1,90 |
| spruzzarsi | R | 5,30 | 2,70 | 5,59 | 2,79 | 4,94 | 2,56 | 5,24 | 2,69 | 5,50 | 2,79 | 4,88 | 2,58 | 5,59 | 2,94 | 6,92 | 2,35 | 4,00 | 2,87 | 5,14 | 2,57 | 4,42 | 2,84 | 6,00 | 2,00 |
| staccare | NR | 6,00 | 2,23 | 6,40 | 2,04 | 5,58 | 2,38 | 5,84 | 2,41 | 6,40 | 2,16 | 5,22 | 2,58 | 6,04 | 2,33 | 6,33 | 2,15 | 5,73 | 2,57 | 6,29 | 1,72 | 6,50 | 1,77 | 6,11 | 1,76 |
| stringersi | R | 6,03 | 2,24 | 6,04 | 2,15 | 6,02 | 2,36 | 6,02 | 2,48 | 6,21 | 2,50 | 5,81 | 2,48 | 5,88 | 2,08 | 5,71 | 1,68 | 6,08 | 2,54 | 6,22 | 1,86 | 6,00 | 1,70 | 6,38 | 2,02 |
| strizzare | NR | 6,42 | 2,32 | 6,04 | 2,28 | 6,75 | 2,33 | 6,63 | 2,51 | 6,09 | 2,81 | 7,12 | 2,16 | 6,37 | 1,98 | 6,00 | 1,71 | 6,77 | 2,24 | 6,00 | 2,28 | 6,00 | 1,51 | 6,00 | 2,71 |
| strofinare | NR | 6,10 | 2,25 | 6,19 | 2,27 | 6,02 | 2,26 | 5,89 | 2,54 | 6,00 | 2,65 | 5,80 | 2,53 | 6,32 | 2,08 | 6,42 | 1,83 | 6,23 | 2,35 | 6,20 | 1,99 | 6,20 | 2,35 | 6,20 | 1,69 |
| suicidarsi | R | 1,67 | 1,70 | 1,08 | 0,36 | 2,15 | 2,16 | 1,89 | 1,52 | 1,21 | 0,58 | 2,30 | 1,77 | 1,50 | 1,86 | 1,00 | 0,00 | 2,00 | 2,57 | 1,50 | 1,82 | 1,00 | 0,00 | 2,00 | 2,54 |
| svitare | NR | 6,04 | 1,71 | 6,00 | 1,72 | 6,08 | 1,73 | 6,16 | 1,90 | 6,44 | 2,06 | 5,88 | 1,75 | 6,00 | 1,68 | 5,46 | 1,20 | 6,58 | 1,98 | 5,92 | 1,53 | 6,00 | 1,66 | 5,80 | 1,40 |
| temere | NR | 2,94 | 2,23 | 2,71 | 2,01 | 3,17 | 2,41 | 3,15 | 2,39 | 3,14 | 2,16 | 3,15 | 2,66 | 2,73 | 2,18 | 2,23 | 1,92 | 3,23 | 2,39 | 2,70 | 1,89 | 2,10 | 1,52 | 3,15 | 2,08 |
| terrorizzare | NR | 4,10 | 2,59 | 3,86 | 2,49 | 4,40 | 2,70 | 4,05 | 2,52 | 3,58 | 2,57 | 4,63 | 2,39 | 4,80 | 2,44 | 4,40 | 2,37 | 5,20 | 2,57 | 3,29 | 2,92 | 4,00 | 2,62 | 2,33 | 3,27 |
| terrorizzarsi | R | 1,81 | 1,77 | 1,82 | 1,85 | 1,80 | 1,71 | 1,73 | 1,62 | 1,77 | 1,81 | 1,69 | 1,41 | 1,88 | 1,88 | 2,00 | 2,18 | 1,75 | 1,54 | 1,92 | 2,04 | 1,73 | 1,62 | 2,08 | 2,40 |
| tirare | NR | 4,54 | 2,15 | 4,56 | 2,15 | 4,51 | 2,18 | 4,29 | 2,43 | 4,61 | 2,54 | 3,80 | 2,24 | 4,63 | 2,14 | 4,46 | 2,26 | 4,82 | 2,09 | 4,87 | 1,66 | 4,58 | 1,16 | 5,18 | 2,09 |
| toccare | NR | 6,40 | 2,27 | 6,28 | 2,28 | 6,51 | 2,29 | 6,30 | 2,33 | 5,88 | 2,22 | 6,58 | 2,41 | 6,46 | 2,00 | 6,71 | 1,90 | 6,17 | 2,17 | 6,53 | 2,59 | 6,30 | 2,91 | 6,78 | 2,33 |
| torturare | NR | 3,06 | 2,06 | 3,02 | 2,15 | 3,10 | 1,98 | 2,58 | 1,78 | 2,46 | 1,69 | 2,71 | 1,90 | 3,40 | 2,04 | 3,00 | 1,61 | 3,89 | 2,47 | 3,89 | 2,49 | 4,56 | 3,13 | 3,22 | 1,56 |
| tradire | NR | 3,10 | 2,54 | 2,91 | 2,57 | 3,31 | 2,53 | 3,65 | 2,71 | 3,50 | 2,85 | 3,83 | 2,58 | 2,23 | 1,97 | 2,00 | 2,32 | 2,50 | 1,51 | 2,83 | 2,51 | 2,55 | 1,75 | 3,08 | 3,07 |
| vendicarsi | R | 3,06 | 2,50 | 3,41 | 2,58 | 2,71 | 2,39 | 3,29 | 2,51 | 4,21 | 2,51 | 2,37 | 2,22 | 3,42 | 2,43 | 3,33 | 2,39 | 3,50 | 2,58 | 2,20 | 2,46 | 2,00 | 2,54 | 2,40 | 2,50 |
| vergognarsi | R | 2,48 | 1,92 | 2,25 | 1,68 | 2,70 | 2,12 | 2,44 | 1,86 | 2,58 | 2,06 | 2,32 | 1,70 | 2,65 | 2,44 | 1,73 | 1,35 | 3,50 | 2,94 | 2,37 | 1,34 | 2,20 | 1,03 | 2,56 | 1,67 |
| versare | NR | 6,54 | 2,10 | 6,71 | 2,09 | 6,32 | 2,12 | 7,05 | 2,10 | 7,17 | 2,17 | 6,87 | 2,07 | 6,20 | 1,63 | 6,54 | 1,85 | 5,83 | 1,34 | 6,05 | 2,44 | 6,00 | 2,13 | 6,10 | 2,88 |
| vestirsi | R | 6,58 | 2,21 | 6,48 | 2,38 | 6,71 | 2,01 | 6,85 | 2,31 | 7,20 | 2,21 | 6,31 | 2,43 | 6,57 | 1,73 | 6,08 | 1,68 | 7,09 | 1,70 | 6,18 | 2,54 | 5,67 | 2,99 | 6,80 | 1,81 |

**Supplementary Table 5** - showing, for each verb, the means and the standard deviations (SDs) for the index of valence. Means and SDs are reported for each age range (8-11 ALL, 12-15 ALL, 16-19 ALL), for males and females within each age range(8-11 females, 8-11 males; 12-15 females, 12-15 males; 16-19 females, 16-19 males), for males and females within the whole sample (females ALL, males ALL) and for the whole sample (ALL).
