# Supplementary material for: Age-Dependent Changes of Thinking about Verbs
Source: Front Behav Neurosci. 2017 Mar 14;11:40. doi: 10.3389/fnbeh.2017.00040 (PMC5348498; doi:10.3389/fnbeh.2017.00040)
Supplement: Supplementary file 7 [file Table7.DOCX]

**Supplementary Table 7 – Verbs’list (Italian 🡪 English)**

| **Italian** | **English** | **R -NR** |  | **Italian** | **English** | **R -NR** |  | **Italian** | **English** | **R -NR** |  | **Italian** | **English** | **R -NR** |
| --- | --- | --- | --- | --- | --- | --- | --- | --- | --- | --- | --- | --- | --- | --- |
|  |  |  |  |  |  |  |  |  |  |  |  |  |  |  |
|  |  |  |  |  |  |  |  |  |  |  |  |  |  |  |
| abbottonarsi | button up | NR |  | fotografarsi | do a selfie | R |  | perdonare | forgive | NR |  | scusarsi | apologize | R |
| afferrare | grab | NR |  | gettare | throw | NR |  | perdonarsi | forgive themselves | R |  | segare | saw | NR |
| affettare | slice | NR |  | graffiarsi | scratch themselves | R |  | perseguitare | stalk | NR |  | sfidare | challenge | NR |
| aggrapparsi | cling | R |  | grattarsi | pick at | R |  | pettinarsi | comb | R |  | sganciare | unhook | NR |
| allacciarsi | fasten | R |  | grattugiare | grate | NR |  | piacere | like | NR |  | slegarsi | untie | R |
| amare | love | NR |  | guarire | yelp | NR |  | piacersi | like themselves | R |  | soffrire | suffer | NR |
| amarsi | love themselves | R |  | impaurirsi | get scared | R |  | piangere | cry | NR |  | sognare | dream | NR |
| ammalarsi | sicken | R |  | impazzire | get creazy | NR |  | piegare | fold | NR |  | sollevare | lift | NR |
| annodare | knot | NR |  | impressionarsi | be shocked | R |  | pinzare | staple | NR |  | sospettare | suspect | NR |
| annoiarsi | be bored | R |  | inchiodare | nail | NR |  | pizzicarsi | nip themselves | R |  | spalmare | spread | NR |
| apparecchiare | lay | NR |  | incollarsi | glue themselves | R |  | posare | pose | NR |  | spaventare | scare | NR |
| appendere | hang | NR |  | indicarsi | indicate themselves | R |  | premere | press | NR |  | spaventarsi | be scared | R |
| applaudire | applaud | NR |  | infilare | tuck | NR |  | prendere | take | NR |  | spazzolarsi | brush themselves | R |
| arrabbiarsi | get angry | R |  | ingelosirsi | be jealous | R |  | preoccuparsi | worry | R |  | sperare | hope | NR |
| avvitare | screw | NR |  | insultare | insult | NR |  | punire | punish | NR |  | spezzare | break | NR |
| avvolgersi | wind | R |  | intrecciarsi | interweave themselves | R |  | punirsi | punish themselves | R |  | spingere | push | NR |
| bussare | knock | NR |  | intristirsi | become sad | R |  | raccogliere | collect | NR |  | spremere | squeeze | NR |
| calmare | calm down | NR |  | invidiare | envy | NR |  | rallegrarsi | rejoice | R |  | spruzzarsi | spray on themselves | R |
| calmarsi | simmer down | R |  | irritarsi | become irritated | NR |  | rastrellare | rake | NR |  | staccare | remove | NR |
| cancellare | erase | NR |  | lanciare | launch | NR |  | rattristarsi | become sad | R |  | stringersi | tighten | R |
| confessare | confess | NR |  | lavarsi | wash up | R |  | ridere | laugh | NR |  | strizzare | wring | NR |
| confondersi | get confused | R |  | legare | tie | NR |  | rifiutare | refuse | NR |  | strofinare | wipe, | NR |
| controllare | check | NR |  | maledire | damn | NR |  | rilassarsi | relax | R |  | suicidarsi | suicide | R |
| controllarsi | control themselves | R |  | martellare | hammer | NR |  | rubare | steal | NR |  | svitare | unscrew | NR |
| credere | believe | NR |  | mentire | lie | NR |  | ruotare | rotate | NR |  | temere | fear | NR |
| decidere | decide | NR |  | meritare | deserve | NR |  | sanguinare | bleed | NR |  | terrorizzare | terrify | NR |
| deludere | disappoint | NR |  | mescolare | stir | NR |  | sbagliare | be wrong | NR |  | terrorizzarsi | be terrified | R |
| deprimersi | get depressed | R |  | morire | die | NR |  | sbattere | beat | NR |  | tirare | pull | NR |
| desiderare | desire | NR |  | obbedire | obey | NR |  | scarabocchiare | scribble | NR |  | toccare | touch | NR |
| dimenticare | forget | NR |  | odiare | hate | NR |  | schiacciare | squash | NR |  | torturare | torture | NR |
| dipingere | paint | NR |  | odiarsi | hate themselves | R |  | sconfiggere | defeat | NR |  | tradire | betray | NR |
| disegnare | draw | NR |  | peccare | sin | NR |  | scoraggiarsi | be discouraged | R |  | vendicarsi | revenge themselves | R |
| dispiacersi | be sorry | R |  | penare | suffer | NR |  | scrivere | write | NR |  | vergognarsi | be ashamed | R |
| fallire | fail | NR |  | pennellarsi | brush themselves | R |  | scuotere | shake | NR |  | versare | pour | NR |
| fingere | pretend | NR |  | perdere | loose | NR |  | scusare | excuse | NR |  | vestirsi | dress | R |

**Supplementary Table 7** - List of verbs - translation from Italian (1st column) to English (2nd column). For each verb it is indicated if, in Italian, it is in its “reflexive” (R) or “non reflexive” (NR) form.
